# Rapid respiratory microbiological point-of-care-testing and antibiotic prescribing in primary care: Protocol for the RAPID-TEST randomised controlled trial

**DOI:** 10.1371/journal.pone.0302302

**Published:** 2024-05-20

**Authors:** Samantha Elizabeth Abbs, Lindsay Armstrong-Buisseret, Kathy Eastwood, Stephen Granier, Athene Lane, Mandy Lui, Chris Metcalfe, Paul Mitchell, Peter Muir, Matthew Ridd, Jodi Taylor, Lucy Yardley, Grace Young, Alastair D. Hay

**Affiliations:** 1 Bristol Trials Centre, Bristol Medical School, University of Bristol, Bristol, United Kingdom; 2 Patient Representative, United Kingdom; 3 Whiteladies Medical Group, Bristol, United Kingdom; 4 Bristol Medical School, University of Bristol, Bristol, United Kingdom; 5 UKHSA South West Regional Laboratory, Southmead Hospital, Bristol, United Kingdom; 6 Centre for Academic Primary Care, Bristol Medical School, University of Bristol, Bristol, United Kingdom; 7 School of Psychological Science, University of Bristol, Bristol, United Kingdom; 8 School of Psychology, University of Southampton, Southampton, United Kingdom; PLOS: Public Library of Science, UNITED STATES

## Abstract

**Background:**

Antibiotics are prescribed for over 50% of respiratory tract infections in primary care, despite good evidence of there being no benefit to the patient, and evidence of over prescribing driving microbial resistance. The high treatment rates are attributed to uncertainty regarding microbiological cause and clinical prognosis. Point-of-care-tests have been proposed as potential antibiotic stewardship tools, with some providing microbiological results in 15 minutes. However, there is little research on their impact on antibiotic use and clinical outcomes in primary care.

**Methods:**

This is a multi-centre, individually randomised controlled trial with mixed-methods investigation of microbial, behavioural and antibiotic mechanisms on outcomes in patients aged 12 months and over presenting to primary care in the UK with a suspected respiratory tract infection, where the clinician and/or patient thinks antibiotic treatment may be, or is, necessary. Once consented, all participants are asked to provide a combined nose and throat swab sample and randomised to have a rapid microbiological point-of-care-test or no point-of-care-test. For intervention patients, clinicians review the result of the test, before contacting the patient to finalise treatment. Treatment decisions are made as per usual care in control group patients. The primary outcome is whether an antibiotic is prescribed at this point. All swab samples are sent to the central laboratory for further testing. Patients are asked to complete a diary to record the severity and duration of symptoms until resolution or day 28, and questionnaires at 2 months about their beliefs and intention to consult for similar future illnesses. Primary care medical records are also reviewed at 6-months to collect further infection consultations, antibiotic prescribing and hospital admissions. The trial aims to recruit 514 patients to achieve 90% power with 5% significance to detect a 15% absolute reduction in antibiotic prescribing.

Qualitative interviews are being conducted with approximately 20 clinicians and 30 participants to understand any changes in beliefs and behaviour resulting from the point-of-care-test and generate attributes for clinician and patient discrete choice experiments.

**Discussion:**

This trial will provide evidence of efficacy, acceptability and mechanisms of action of a rapid microbiological point-of-care test on antibiotic prescribing and patient symptoms in primary care.

**Trial registration:**

ISRCTN16039192, prospectively registered on 08/11/2022.

## Introduction

Respiratory tract infections (RTIs) are the most common problem managed by health services worldwide [1]. In the UK, antibiotics are prescribed for over 50% of RTIs by general practitioners (GP) and primary care nurses [2, 3], with 50% of these prescriptions considered inappropriate [4, 5], and despite strong evidence that the majority of patients do not benefit [6–9]. The overprescribing of antibiotics results in unnecessary side effects [10], depletion of normal flora [11], increased healthcare costs [12], and fuels antimicrobial resistance (AMR) [13, 14], which is regarded as a significant public health threat [15]. High antibiotic treatment rates are attributed to clinician uncertainty regarding patients’ microbiological diagnosis and clinical prognosis [16, 17], leading to ‘just-in-case’ defensive prescribing [17].

One potential solution to address this issue is point-of care-testing (POCT) in primary care settings. Rapid microbiological POCTs (POCT^RMs^) use nucleic acid or antigen-based tests to detect viruses and bacteria from respiratory tract samples in as little as 15 minutes [18]. While multiplex POCT^RMs^ have shown promising results in reducing hospital admission times and length of antibiotic courses in secondary care [19–22], more research is needed to evaluate their impact on antibiotic use and clinical outcomes in primary care.

Observational studies [23, 24] have reported on the effects of POCT^RMs^ testing for Influenza A and B, and/or respiratory syncytial virus (RSV) in primary care. However, none of these studies evaluated the use of multiplex POCT^RMs^ on antibiotic prescribing or clinical outcomes. We conducted a mixed-methods, observational feasibility study [25] which suggested a multiplex POCT^RM^ was acceptable in primary care, and helped improve diagnostic certainty. However, none of these studies used randomised controlled trial methods, and none assessed the short- and long-term effects of POCT^RM^ testing on clinician and patient beliefs and behaviour.

To address this evidence gap, the RAPID-TEST study will conduct a RCT with the aim of comparing decision making, antibiotic prescribing and patient outcomes between individuals allocated to a POCT group and those allocated to a usual care group, at approximately 16 primary care practices in the south west of England.

## Material and methods

### Design and setting

This is a multi-centre, individually randomised controlled trial with a mixed-methods investigation of microbial, behavioural and antibiotic mechanisms, conducted in UK primary care. The trial is using eight POCT ^RM^ machines and recruiting from 16 GP (family doctor) practices across the South West of England, with eight sites recruiting participants per Winter. Sites with previous research experience and those who were confident they could deliver the relatively complex trial design were prioritised during site selection.

### Population

The trial is aiming to recruit 514 patients aged ≥12 months presenting to primary care with a suspected RTI where the Study Clinician and/or patient believes antibiotic treatment is, or may be, necessary. Patients can present to their GP practice face-to-face, via telephone or via online appointment. Study Clinicians can be any member of staff at the practice that is usually responsible for the care of patients with RTIs (GPs, nurses, paramedics and pharmacists) and who have been trained in study procedures.

Patients must meet all the following criteria to be eligible to take part:

1. Aged ≥12 months on the day of presentation
2. Presenting to primary care for the first time in this episode, and within 21 days of illness onset, with a Study Clinician suspected acute RTI. Symptoms may include one or more of:
  - Sore throat
  - Runny nose
  - Earache
  - Cough
  - Sputum
  - Wheeze
  - Shortness of breath
3. Study Clinician diagnoses of an upper or lower RTI such as:
  - Acute otitis media
  - Acute sinusitis
  - Acute pharyngitis or tonsillitis
  - Sore throat
  - Acute laryngitis
  - Acute cough
  - Acute bronchitis
  - Chest infection
  - Acute lower RTI
  - Infective exacerbation of chronic lung disease e.g. asthma, chronic obstructive pulmonary disease (COPD), emphysema or bronchiectasis
4. Study Clinician or patient/parent/carer believes antibiotic treatment is, or may be, necessary
5. Patient/parent/carer willing and able to give informed consent
6. Patient/parent/carer willing for patient to have a nasal and throat swab taken, or willing and able to collect, self-take and promptly return the swab to the site
7. Study Clinician and patient/parent/carer willing to wait for the POCT^RM^ result before an antibiotic prescribing decision is made
8. Laboratory transport pick up for samples expected <24 hours e.g. sample is expected to be ready prior to final sample collection on a Friday
9. Patient/parent/carer willing to complete Trial Diary and for outcome data to be collected from medical record

Patients with any of the following criteria are excluded:

1. Known to have cystic fibrosis
2. Require hospital admission
3. Previously taken part in the RAPID-TEST RCT
4. Currently taking part in another conflicting RTI study.

Patients already being treated with antibiotics or antivirals (for any indication) are eligible as long as the Study Clinician suspects a new (or ongoing) RTI, and the Study Clinician and/or patient/ parent/ carer believe further antibiotic treatment is, or may be, necessary.

Co-enrolment to other research studies are considered on a case-by-case basis.

### Objectives

#### Clinical objectives

The primary clinical objective is to investigate whether the use of a rapid POCT^RM^ can reduce same-day antibiotic prescribing for children and adults presenting to primary care with RTIs where the Study Clinician and/or patient believes antibiotic treatment is, or may be, necessary.

The key secondary clinical objective is to investigate whether the use of a rapid POCT^RM^ changes participant reported symptom severity on days 2 to 4.

Other clinical secondary objectives include:

a) To investigate whether the use of a rapid POCT^RM^ changes participant reported severity and duration of symptoms over 28 days including any hospital admissions for respiratory symptoms, antibiotic consumption, and time to return to normal activities.
b) To investigate whether the use of a rapid POCT^RM^ changes participant (or parent/carer if the participant is <16 years) confidence in the clinical management of the infection, and their intention to consult for future similar illnesses
c) To investigate whether the use of a rapid POCT^RM^ changes subsequent healthcare resource use in the following six months

#### Qualitative objectives

d) To explore participants’ (or parents’/carers’ if the participant is <16 years) understanding of the test and the result they were given, and their views of the implications for treatment and future consultations
e) To explore the trade-offs participants (or parents/carers if the participant is <16 years) make in choosing to visit the GP with respiratory infection symptoms and the extent POCT^RMs^ might increase or decrease help seeking behaviour
f) To describe the situations in which clinicians most and least value the new microbial knowledge, and how it may influence clinical reasoning and participant management
g) To explore the trade-offs clinicians make about whether and when to use the POCT^RM^

### Mechanistic objectives

h) To determine whether there are overall (intervention vs. control) and differential (virus detected vs. not detected) effects with respect to reducing the number of participants for whom the Study Clinician believes antibiotics are necessary
i) To describe the effect of POCT^RM^ results on Study Clinician and participant (or parent/carer if the participant is <16 years) beliefs in the necessity, and benefits, of prescribing antibiotics for the respiratory infection, and confidence in the value of the POCT^RM^ to guide the prescribing decision and explore relationships of Study Clinician and participant (or parent/carer if the participant is <16 years) beliefs, attitudes and intentions with antibiotic prescribing and consumption.

### Patient identification, ‘Appointment one’ and consent

**Fig 1:**
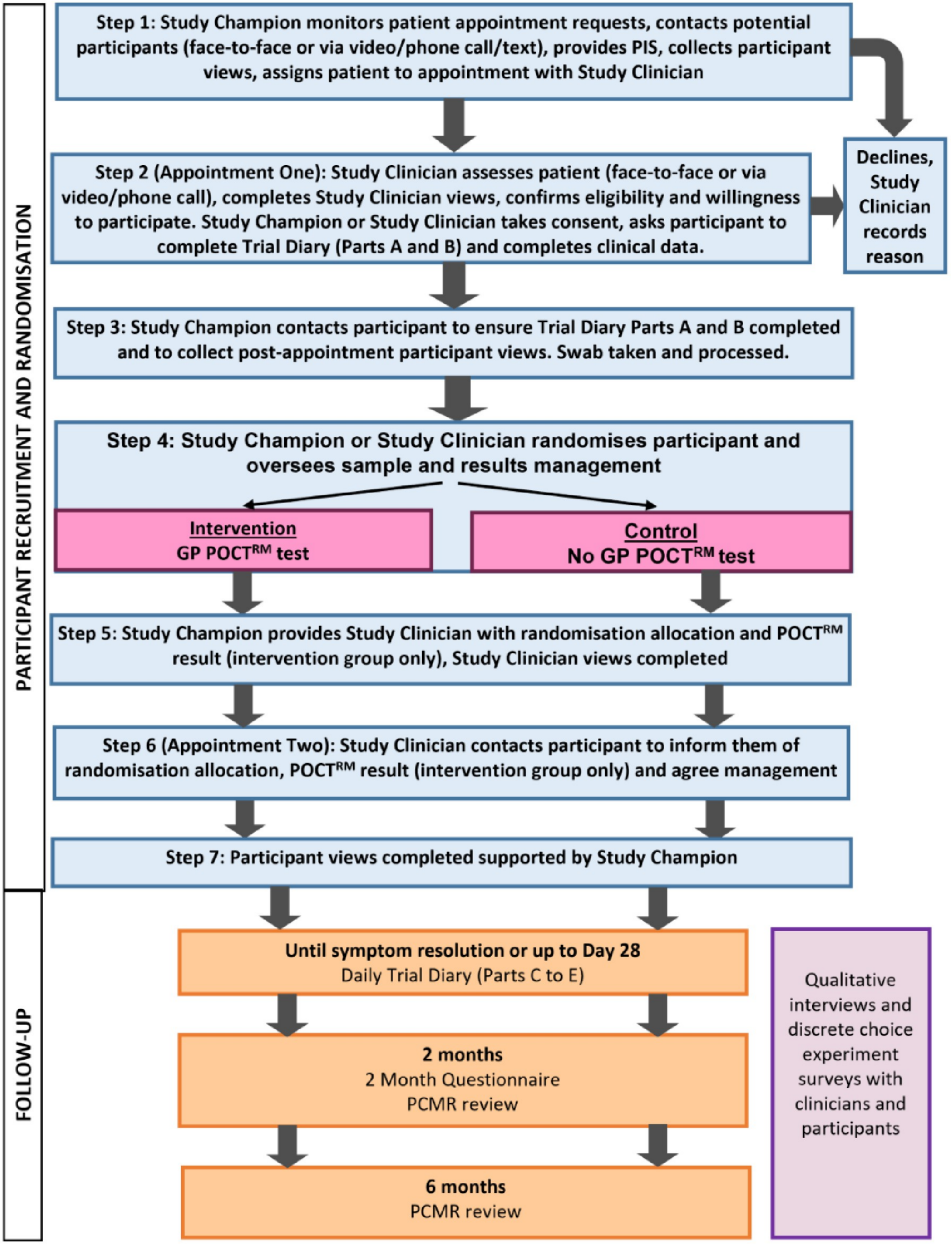
Participant flow diagram.

Fig 1 represents how participants progress throughout the trial and the different activities that take place at each stage.

Potentially eligible patients, identified from appointment request lists by a member of the GP practice, are offered a Participant Information Sheet, on paper or electronically. The ‘Study Champion’ (e.g. receptionist, healthcare assistant, medical student, pharmacist, paramedic, practice nurse or manager) explains the trial to the patient and answers any questions. Patients are asked to answer three questions regarding their views on their need for antibiotics (see Table 1. **Participant and Study Clinician views**). These are asked before the patient sees the Study Clinician at ‘Appointment One’ since this interaction could change their views.

**Table 1:** Participant and study clinician views.

Mediating variable	Measures to be used (steps according to Fig 2)	Response options
*Participant Views (or parent/carer if the participant is <16 years)*		
Expectation that antibiotics are needed	1. I believe an antibiotic is needed to treat my/my child’s illness [Steps 1, 3 and 7] 2. I believe my/my child’s illness will get better faster if I/they take an antibiotic [Steps 1, 3 and 7] 3. I believe my/my child’s illness will be less severe if I/they am/are given an antibiotic [Steps 1, 3 and 7]	Strongly disagree; Disagree; Neither agree nor disagree; Agree; Strongly agree
Confidence to manage illness without antibiotics (self-efficacy)	4. A point-of-care test would help in making the right decision about whether I/my child need/needs antibiotics [Step 3] 5. A point-of-care test would have helped/has helped in making the right decision about whether I/my child need/needs antibiotics [Step 7] 6. I am confident that I/my child will get/am getting/is getting the right treatment [Steps 3 and 7]	Strongly disagree; Disagree; Neither agree nor disagree; Agree; Strongly agree
Confidence to manage similar infection without antibiotics in the future	7. If I/my child have/has an infection in future that is like the one I/they had when I/they joined this trial then I/we will see my/our doctor to check if antibiotics are needed [2 Month Questionnaire] 8. If I/my child have/has an infection in future that is like the one I/they had when I/they joined this trial then I/we would like to have a point-of-care test to check if antibiotics are needed [2 Month Questionnaire]	Strongly disagree; Disagree; Neither agree nor disagree; Agree; Strongly agree
*Study Clinician Views*		
Expectation that antibiotics are needed	1. I believe an antibiotic is needed to treat the patient’s illness [Steps 2 and 5] 2. I believe the patient’s illness will improve faster if I prescribe an antibiotic [Steps 2 and 5] 3. I believe the patient’s illness will be less severe if I prescribe an antibiotic [Steps 2 and 5]	Strongly disagree; Disagree; Neither agree nor disagree; Agree; Strongly agree
Confidence to manage patient without antibiotics (self-efficacy)	4. The point-of-care test would help in making the right decision about whether the patient needs antibiotics [Step 2] 5. The point-of-care test would have helped/has helped in making the right decision about whether the patient needs antibiotics [Step 5] 6. I am confident that the patient will believe they are getting the right treatment [Steps 2 and 5]	Strongly disagree; Disagree; Neither agree nor disagree; Agree; Strongly agree
Confidence to manage similar infection without antibiotics in the future	7. If a patient has a similar infection in future I am likely to prescribe them antibiotics [Step 5] 8. If a patient has a similar illness in future I would like to use the POCT^RM^ [Step 5]	Strongly disagree; Disagree; Neither agree nor disagree; Agree; Strongly agree

At Appointment One, the Study Clinician assesses the patient as per usual care. They confirm eligibility and completes their ‘Study Clinician Views’ (see Table 1. **Participant and Study Clinician views**) on whether they consider the patient requires antibiotics.

If eligible and willing to take part, the patient completes a consent form, either electronically, on paper or verbally (in the presence of an independent witness). For patients aged under 16 years, the consent form is completed by their parent/carer. Patients aged 12–15 years old can also complete an assent form.

Once consent has been provided, the participant is asked to complete a second set of ‘Participant Views’ and baseline questions about their symptoms and an EQ-5D-5L questionnaire.

All participants are asked to provide a combined nose and throat swab using MWE Sigma Σ-VIROCULT^®^ swab kits provided by the central trial team. Where possible, these swabs will be collected by a member of staff at the GP practice, but self-swabbing is permitted if necessary. Swabs are placed in a tube with viral transport medium (VTM) before being tested at the practice, if randomised to the intervention, and sent to the central laboratory.

### Randomisation

Once a swab has been provided, participants are individually randomised 1:1 to intervention (GP POCT^RM^ test) or control (No GP POCT^RM^ test) using an internet-based randomisation system developed and maintained by Sealed Envelope^™^ to ensure concealment. Randomisation is stratified by age (<16 years vs. ≥16 years) and chronic lung disease, defined as asthma, chronic obstructive pulmonary disease (COPD), emphysema or bronchiectasis (present vs. absent). It is not possible to blind allocation since subsequent trial processes differ by group. Immediately following randomisation, the Study Clinician is informed of the allocation so that clinical management of control group patients can proceed (management of intervention group participants should wait for POCT^RM^ results).

### Intervention

A portion of the VTM from the swab sample from participants in the GP POCT^RM^ test group is analysed as soon as possible using the BioFire^®^ FilmArray^®^ Torch 1 in conjunction with BioFire^®^ RP2.1 *plus* reagent pouches (Biomerieux) according to the manufacturer’s instructions at the GP practice. The time for processing one swab to results being available is approximately 1 hour, assuming the Torch 1 machine is not already in use. The results indicate presence or absence of 23 upper respiratory microbes: 19 viruses (Influenza A (no subtype detected, H1, H1-2009, H3), Influenza B, Adenovirus, Coronaviruses (HKU1, NL63, 229E, OC43, Mers-CoV, SARS-CoV-2), Human Metapneumovirus, Human Rhinovirus/ Enterovirus (not possible to distinguish due to genetic similarity), Parainfluenza (types 1, 2, 3, 4) and RSV and four atypical bacteria: *Bordetella pertussis*, *Bordetella parapertussis*, *Chlamydia pneumoniae and Mycoplasma pneumonia*.

Study Clinicians are advised during trial set up and training that the POCT^RM^ result should be used as a guide to clinical decision making, with final responsibility for antibiotic prescribing residing with the Study Clinician. To help with results interpretation, Study Clinicians are provided with information describing the typical presentation of illnesses caused by the microbes tested (S1 Appendix).

If the POCT^RM^ results are reported as ‘failed’, ‘invalid’ or ‘equivocal’, the original swab sample is retested as per the manufacturer’s instructions. If it is not possible to obtain POCT^RM^ results after following the manufacturer’s instructions the participant continues in the trial and the Study Clinician makes an antibiotic prescribing decision based on the clinical evidence available at that time.

### ‘Appointment two’

Appointment two takes place on the same day as randomisation.

For participants randomised to the GP POCT^RM^ group, the Study Clinician waits to receive the POCT^RM^ test results, and then completes a second set of ‘Study Clinician Views’ (Table 1) and then contacts the participant to inform them of the randomisation outcome and discuss treatment.

If the participant has been randomised to the No GP POCT^RM^ group, the Study Clinician can proceed to complete their second set of ‘Study Clinician Views’, contact the participant, inform them of the randomisation outcome and discuss treatment.

### Control and central laboratory testing

The remaining swab sample from all (intervention and control) participants is stored at ambient temperature at the GP practice prior to transfer to the central research laboratory (UKHSA South West Regional Laboratory, Southmead Hospital, Bristo) within 24 hours of collection. Samples not sent on the same day of collection are stored in the GP practice fridge (2 to 8°C) overnight. At the central laboratory, these samples are tested for the presence of respiratory viral and bacterial pathogens using a Taqman Low Density polymerase chain reaction array card assay [26] as well as the same BioFire^®^ RP2.1 *plus* reagent pouches run on the Biofire^®^ Filmarray^®^ Torch 1 system used at the GP practices. At the end of the study any residual sample will be destroyed. All samples are handled according to the Human Tissue Act.

### Follow up

All participants (or parent/carer if the participant is <16 years) are asked to complete a validated Trial Diary [27] until symptoms resolve (defined as all symptoms being rated zero for two consecutive days) or up to day 28, whichever comes first. The central trial team contacts participants via email, text or phone to support completion of the Trial Diary.

Participants (or parent/carer if the participant is <16 years) are sent a follow-up questionnaire at 2 months to collect beliefs and intention to consult for similar future illnesses (see S1 Appendix). Up to two reminders are sent where questionnaires are not returned.

In addition to Trial Diaries and questionnaires, data on any further GP consultations for RTIs are collected from participants’ primary care medical records (PCMR) at 2 and 6 months by site staff.

### Optional qualitative interviews and Discrete Choice Experiment (DCE) surveys

During the consent process, participants (or parents/carers if the participant is <16 years) are asked whether they are interested in being contacted about taking part in an optional Participant Interview and receiving a separate optional Participant Discrete Choice Experiment (DCE) survey. Participants (or parents/carers if the participant is <16 years) can choose to be contacted about the interview and the DCE survey, just one, or neither. This does not affect their ability to take part in the main trial.

Clinicians from each site are asked whether they are interested in being contacted about taking part in an optional Clinician Interview. In addition, Study Clinicians involved in the trial and other clinicians recruited through the West of England Clinical Research Network will be invited to complete a Clinician DCE survey.

A DCE is a method commonly used in health economics to elicit stated preferences from health care stakeholders.[28] Participant and Clinician DCE surveys will be developed based on the qualitative interview findings with participants and clinicians, respectively.

### Outcome measures

The primary clinical efficacy outcome is antibiotic prescribing for a RTI at Appointment Two, as reported by Study Clinicians and/or collected from participants’ medical records.

The key secondary clinical outcome is symptom severity on days 2 to 4, as reported on Trial Diary.

Other clinical secondary outcomes include:

1. Symptom severity and duration, length of time to return to usual activities, and antibiotic and antiviral consumption within 28 days as reported by patient on Trial Diary.
2. Health-related quality of life measured by the EQ-5D-5L (age >16 years) or EQ-5D-Y (age 1–15 years)
3. Participant and clinician views (see Table 1)
4. Hospital admissions within 28 days
5. Consultations for respiratory infections within 6 months
6. Name and dose of antibiotics and antivirals prescribed within 28 days

### Data collection and management

**Fig 2:**
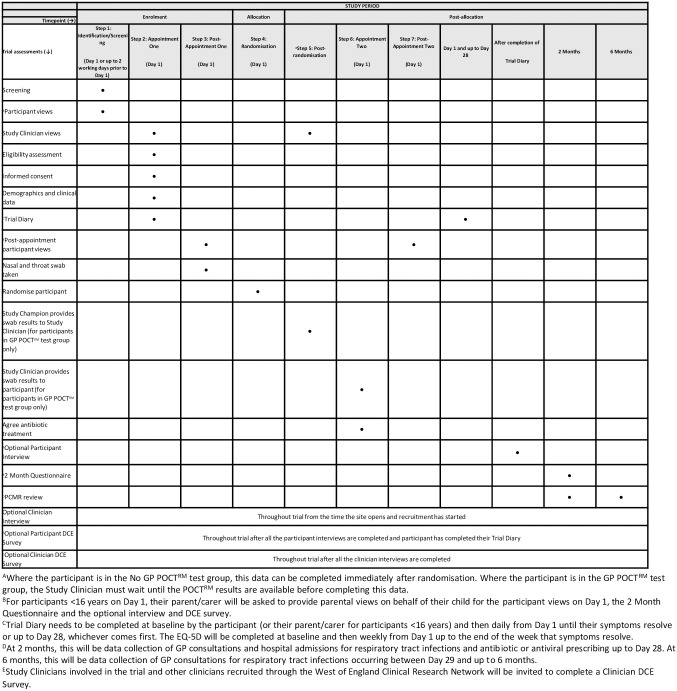
Schedule of enrolment, interventions and assessments.

The data collection schedule is outlined in Fig 2. Data is directly entered onto a bespoke REDCap database and stored on a secure server, only accessible to authorised staff. Patient reported Trial Diary and questionnaire data are also recorded and stored on the trial database All study documentation will be retained in a secure location during the study and for at least 5 years after the end of the study. For children under the age of 16 at recruitment, research data will be kept until their 25th birthday.

### Safety reporting

**Fig 3:**
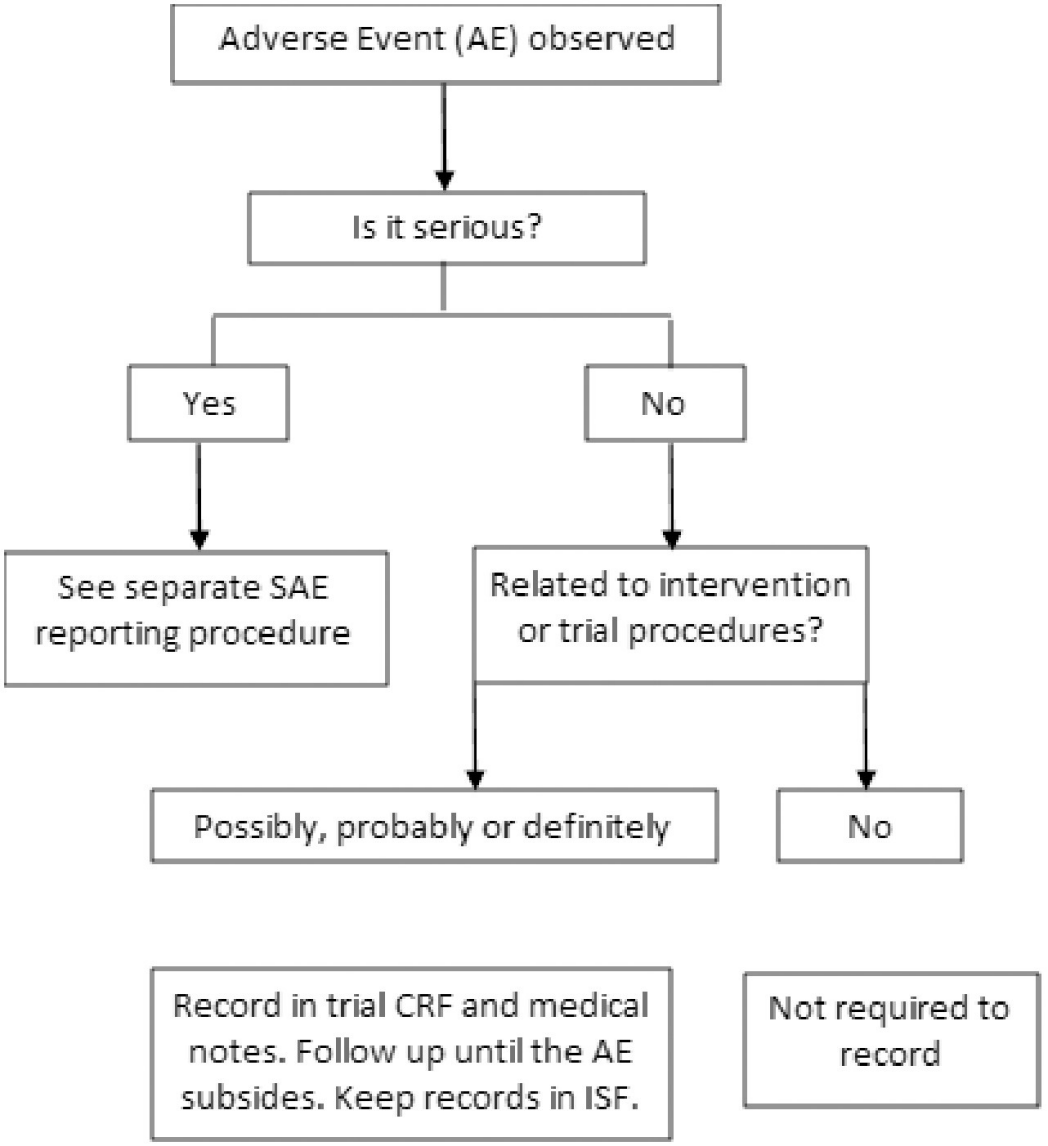
Recording framework for AEs assessed as non-serious.

Site staff are responsible for recording appropriate adverse events (AEs) for the participants during the trial. Only non-serious AEs that are assessed as being possibly, probably or definitely related to the intervention or trial procedures will be recorded in the relevant trial documentation. They should also be recorded in the participant’s medical notes by site team and the participant should be followed up until the event resolves. Non-serious AEs that are unrelated to the intervention do not need to be recorded. The reporting framework for non-serious AEs is shown in Fig 3.

**Fig 4:**
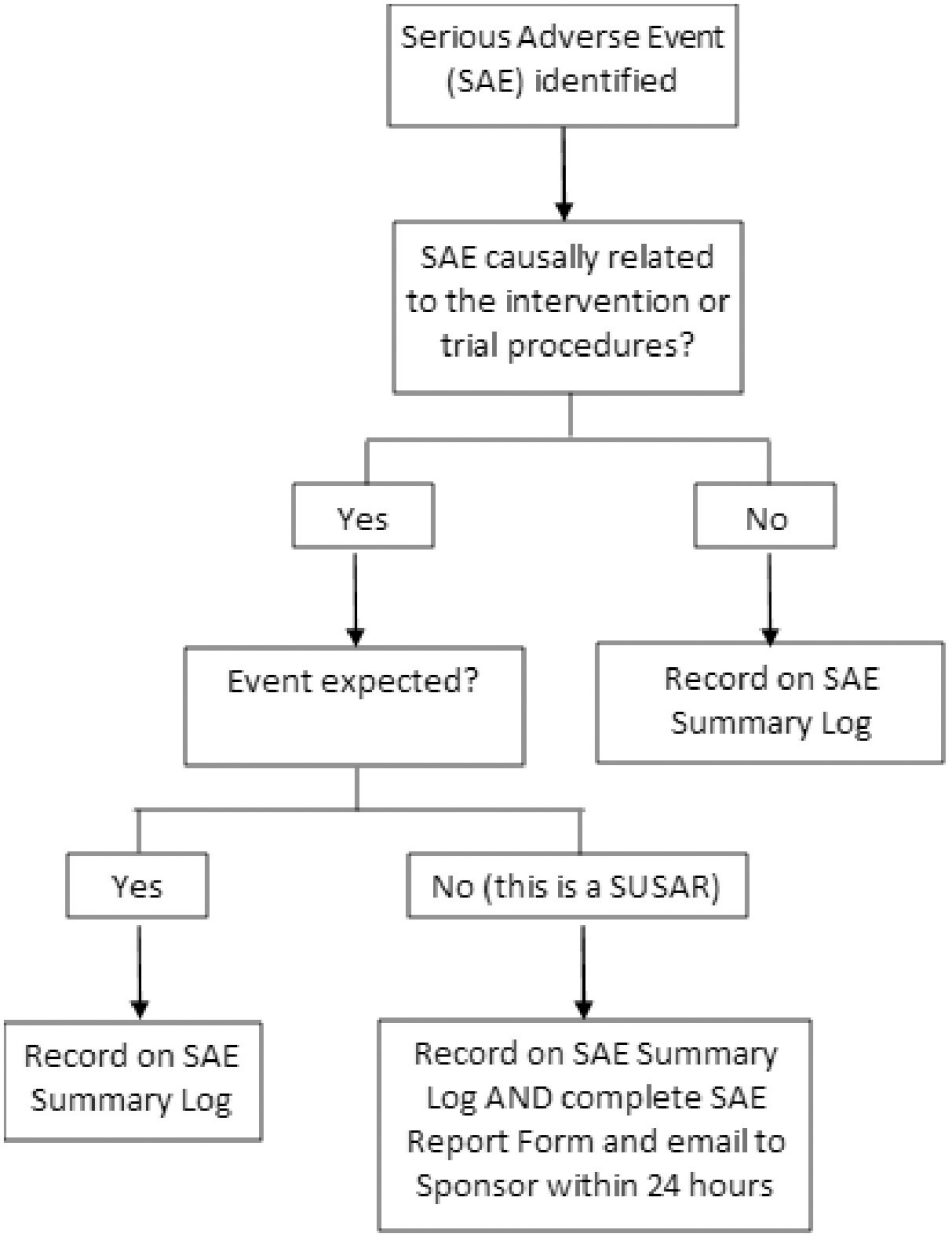
Recording framework for serious adverse events (SAEs).

Sites are also required to record all serious AEs (SAE) on the trial database. These should also be recorded in the participant’s medical notes and should be followed up by the site until the event resolves. Any SAEs which are assessed by the local Principal Investigator (PI) as being related to the trial procedures and unexpected for the procedure are classed as Suspected Unexpected Serious Adverse Reactions (SUSAR) and must be documented on the full SAE report form and submitted within 24 hours of staff becoming aware of the event. As is proportionate to the nature of the trial, only SUSARs will require expediting reporting to the Sponsor. The reporting framework for SAEs is shown in Fig 4.

All SAEs will be further reported to the DMC as part of their oversight meetings.

### Sample size

Antibiotic prescribing at the index consultation is the primary outcome measure. The largest individual patient data meta-analysis to date [3] reported a total of 65% use of antibiotics in patients in observational and experimental studies, across a wide spectrum of respiratory infections and patient groups, as we propose in RAPID-TEST.

Previous stewardship trials of POCTs have used varying minimum clinically important difference (MCID) definitions of absolute prescribing reductions, from 10% [29] to 20% [30], with actual reductions observed of 15% [29] and 22%[30]. Since POCT^RMs^ are expensive, we selected a MCID of 15%.

Assuming an antibiotic prescribing rate of 60% in the control group, 244 participants per group will allow a true reduction to 45% in the GP POCT^RM^ test group to be detected with 90% power at 5% significance. A total randomisation target of 514 will allow for 5% attrition. We have assumed a relatively large minimum clinically important difference due to the high cost of POCT^RM^.

If the POCT^RM^ results in fewer antibiotic prescriptions, we wish to demonstrate non-inferiority of the POCT^RM^ in terms of not increasing mean symptom severity at days 2 to 4 to a clinically significant extent. Assuming 80% completion of Trial Diaries (as previously achieved in adults [31] and children [32]) we will have data for symptom severity at 2 to 4 days in 206 participants per group. Data on 7,000 adults and children managed without POCT indicates a mean symptom severity at days 2 to 4 of 2.3 (standard deviation 1.5) [3]. We know this measure’s distribution is positively skewed and have used a calculation that accommodates this (assuming equal skew in both groups, quantified as a coefficient of variation of 0.7) [33]. Assuming, in truth, no difference between groups, 206 participants in each group will give 90% power for a one-sided 95% confidence interval to exclude increases in the average symptom score of 20% or more.

### Statistical analysis

A detailed statistical analysis plan will be finalised and made publicly available ahead of the completion of recruitment. In outline, the primary analysis will be of observed data and conducted according to the intention to treat principle. A logistic regression equation will estimate the causal association between the primary outcome of antibiotic prescribing and allocated intervention group as an odds ratio, presented with 95% confidence interval and p-value. Further covariates will include participant age and chronic lung disease (used to stratify randomisation). Variations between participating GPs in prescribing tendency will be accommodated as dummy variables to distinguish each GP. Sensitivity analyses will gauge the robustness of the conclusions to different assumptions about any missing data. The above approach will be adapted e.g. through the choice of a suitable regression model, to the secondary outcome variables such as symptom severity at 2 to 4 days.

Potential mechanisms of action, linking the intervention with the prescribing of an antibiotic will be investigated. A key potential mechanism is the result of the POCT^RM^ test which will be investigated in a logistic regression models with covariates including allocated group (results inform the clinical decision or not), result (virus detected or not), and the interaction between the two. The interaction term will capture any evidence that the test result is influencing the prescribing decision, rather than a non-specific effect of the POCT^RM^ which is independent of the result, and distinguish this effect from any underlying ability of GPs to prescribe to those participants who will benefit from an antibiotic.

### Study management

The study is managed by the Bristol Trials Centre and sponsored by University of Bristol. The Trial Management Group (TMG) includes members responsible for the day-to-day management of the trial, including the chief investigator, co-investigators, trial manager, statistician and patient representatives. The role of the TMG is to monitor the progress and conduct of the trial, including adherence to the trial protocol. The Trial Steering Committee (TSC) is made up of representatives from the RAPID-TEST trial team and independent members approved by the funder. The Data Monitoring Committee (DMC) consists of an independent medical statistician and medical experts in the field also approved by the funder. The TSC and DMC meet as required; at least once a year.

### Patient and public involvement (PPI)

Patients and members of the public have been involved in the design and management of this trial and will also be involved in dissemination activities. Multiple PPI meetings took place at the grant application stage and provided input into the final design on the trial.

The RAPID-TEST Trial Management Group and Independent Steering Committee both include PPI members. PPI members of these groups have provided feedback on Patient Information Sheets provided to trial participants, contributed to draft reports to funders and oversight committee meetings, and have provided input to develop strategies to improve trial diversity and inclusivity.

### Ethical considerations and dissemination

The trial was approved by the North West—Preston Research Ethics Committee on 11th October 2022 (REC reference 22/NW/0294). Any amendments to the trial protocol will be approved by the Sponsor and ethics committee before implementing at sites. Written, informed consent (or verbal consent where written is not possible) to participate will be obtained from all participants (or the parent/carer if under 16 years old).

A plan to disseminate the trial results will be developed by the TMG. The trial team plan to present findings at national and international meetings, and in peer-reviewed publications. Innovative methods of dissemination will be explored such as videos and blogs to accompany scientific papers that are accessible to the public, as well as providing a lay summary to participants.

### Trial status

The trial opened to recruitment on 28th November 2022 and is currently using protocol v3.0, 06 March 2023. The trial is recruiting well and recruitment is expected to finish within the original timelines (by 30th September 2024).

## Discussion

The ‘holy grail’ of antimicrobial stewardship is to ensure the minority of patients needing antibiotics are given the shortest course of the narrowest spectrum treatment possible, while preventing unnecessary exposure among the majority unlikely to benefit. The vast majority of stewardship interventions currently available aim to reduce overall prescribing [34], with only a handful of validated tools available to support precision prescribing in primary care [35, 36].

Given that a significant proportion of RTIs managed in primary care are considered viral [37–39], it is plausible that a POCT^RM^ providing accurate results quickly could improve prescribing. However, there is currently insufficient evidence regarding the safety, efficacy, or mechanism of action, let alone clinical and cost effectiveness, to recommend use of POCT^RMs^ in primary care. Efficacy studies are urgently needed to address these issues using randomised controlled designs, as well as qualitative methods to understand clinician and patients’ perceptions of the tests. One of the strengths of the RAPID-TEST trial is its randomised controlled trial design, which will be delivered in NHS GP practices. This RCT will show whether using multiplex POCT^RMs^ in primary care could safely reduce the unnecessary use of antibiotics, and the beliefs that sustain unnecessary use, thereby reducing the public AMR risk. The trial will also show whether POCT^RM^ use affects clinically relevant patient outcomes, antiviral prescribing, re-consultations and future consultations for RTIs. Although the use of the test in the intervention group slightly extends the initial consultation time, it is possible that this longer consultation could reduce re-consultations in the future. Using a mixed-methos approach, the trial will also provide further insight into behavioural changes, such as how the POCT^RM^ might influence clinician and patient beliefs and confidence in the treatment decision to reduce antibiotic prescribing.

The authors acknowledge the trial’s limitations. Participants are unblinded when completing the Trial Diary, which could lead to bias in patient-reported outcomes, and there is no cost-effectiveness analysis at this stage. The POCT^RM^ used in the trial does not test for typical bacteria such as S. pneumoniae, S. pyogenes, H. influenzae or M. catarrhalis, since these can be commensally carried in the upper respiratory tract, and the test is unable to determine whether any pathogens that are detected are the cause of the patient’s symptoms.

The authors are also aware of similar ongoing trials, also evaluating the use of POCTs in RTIs in primary care, such as PRUDENCE (ISRCTN13336322). However, these trials are evaluating different types of POCT.

Recruitment to RAPID-TEST opened in November 2022 and the trial has proven to be acceptable to patients and clinicians so far, with recruitment ahead of target and expected to be completed within the original budget and timelines.

## Supporting information

S1 Appendix Information provided to clinicians. (DOCX)

S1 Checklist SPIRIT checklist. (DOCX)

S1 File RAPID-TEST protocol v3.0 06Mar23. (PDF)

S2 File RAPID-TEST PIS v2.0 04Oct22. (PDF)

S3 File RAPID-TEST ICF v2.0 04Oct22. (PDF)

S4 File RAPID-TEST protocol v2.0 04Oct22 (original approved protocol). (PDF)

## Abbreviations

AE: Adverse Event
AMR: Antimicrobial resistance
DCE: Discrete Choice Experiment
GP: General practitioner
MCID: Minimally clinically important difference
PCMR: Primary care medical record
POCT: Point-of-care-test
PPI: Patient and public involvement
RCT: Randomised Controlled Trial
RSV: Respiratory Syncytial virus
RTI: Respiratory Tract Infection
SAE: Serious Adverse Event
SUSAR: Suspected Unexpected Serious Adverse Reaction
VTM: Viral Transport Medium
