# Supplementary material for: Rapid respiratory microbiological point-of-care-testing and antibiotic prescribing in primary care: Protocol for the RAPID-TEST randomised controlled trial
Source: PLoS One. 2024 May 20;19(5):e0302302. doi: 10.1371/journal.pone.0302302 (PMC11104596; doi:10.1371/journal.pone.0302302)
Supplement: S1 Appendix — (DOCX) [file pone.0302302.s001.docx]

**Appendix 1. Information provided to clinicians to interpret POCT results**

**Interpreting BioFire Results: Clinician Information Sheet**

# Background and purpose of this information sheet

1. Primary care clinicians usually make antibiotic prescribing decisions in the absence of any contemporaneous microbiological information.
2. The purpose of the RAPID-TEST trial is to investigate if use of the BioFire® Respiratory panel 2.1 *plus*(40) run on the BioFire® FilmArray® Torch system can reduce same-day antibiotic prescribing in patients aged ≥12 months attending primary care with respiratory tract infections.
3. This sheet aims to provide information to help interpret BioFire results during the RAPID-TEST trial.

# What does the BioFire system test, and not test, for?

The BioFire® FilmArray® system detects the presence/absence of the following 23 upper respiratory microbes:

1. 19 viruses
   - Adenovirus
   - Coronaviruses (229E, HKU1, NL63, OC43, MERS-CoV, SARS-CoV-2)
   - Human Metapneumovirus
   - Human Rhinovirus/Enterovirus*

* Not possible to distinguish due to genetic similarity

- - Influenza A (no subtype detected, A H1, A H3, A H1-2009)
  - Influenza B
  - Parainfluenza (types 1, 2, 3, 4)
  - Respiratory Syncytial Virus

1. Four atypical bacteria
   - *Bordetella parapertussis*
   - *Bordetella pertussis*
   - *Chlamydia pneumoniae*
   - *Mycoplasma pneumoniae*

It **does not** test for the typical respiratory bacteria *S. pneumoniae, S. pyogenes, H. influenzae* or *M. catarrhalis* since these can be commensally carried in the upper respiratory tract.

# Clinical presentation associated with microbes

1. Many viral respiratory infections have similar symptoms, commonly including fever, runny/blocked nose, cough (dry and/or productive) and sore throat.
2. **All BioFire detected microbes can cause a wide range of illnesses from mild to severe.**
3. The table below summarises the more specific syndrome presentations associated with some of the BioFire tested microbes.

**Table summarising specific syndrome presentations associated with some BioFire tested microbes**

| ***Microbe*** | ***Typical presentation*** | ***Comment*** |
| --- | --- | --- |
| *Viruses* | | |
| Influenza viruses | Influenza-like-illness | **Notifiable disease** |
| SARS-CoV-2 | COVID-19 | **Notifiable disease** |
| Respiratory Syncytial Virus | Bronchiolitis in young children, acute lower respiratory infection in others |  |
| Parainfluenza Viruses | Croup in young children |  |
| Rhinovirus | Common cold |  |
| Enterovirus | Very broad range of presentations,(41) including fever, respiratory symptoms, rash, gastroenteritis symptoms and rarely meningitis (usually in children under 3 months)* |  |
| Adenovirus | Conjunctivitis, rash, gastroenteritis, hepatitis (rarely) |  |
| MERS-CoV | Causes Middle East Respiratory Syndrome, a severe respiratory illness in travellers from endemic regions | Seek microbiological advice immediately |
| Seasonal Coronaviruses | Common cold, sore throat |  |
| Human Metapneumovirus | Common cold, sore throat |  |
| *Atypical bacteria* | | |
| *Mycoplasma pneumoniae* | Outbreaks approximately every four years, can be self-limiting/ associated with “wheezy bronchitis” and pneumonia |  |
| *Chlamydia pneumoniae* | Can be self-limiting/ associated with pneumonia |  |
| *Bordetella pertussis* | Whooping cough, prolonged cough | **Notifiable disease** |
| *Bordetella parapertussis* | Whooping cough, prolonged cough | **Notifiable disease** |

* children <12 months excluded from RAPID-TEST trial

** <https://www.gov.uk/guidance/notifiable-diseases-and-causative-organisms-how-to-report#list-of-notifiable-diseases>

# Considerations when interpreting BioFire results

| **The BioFire result should be used as a guide to clinical decision making, with final responsibility for how the patient is managed resting with the Study Clinician** |
| --- |

1. Remember, BioFire **does not** test for the main typical respiratory bacteria (*S. pneumoniae, S. pyogenes, H. influenzae* or *M. catarrhalis* since these can be commensally carried in the upper respiratory tract)
2. Where one or more microbes are detected, you may wish to consider if their presence seems consistent, or inconsistent, with the clinical presentation (see Table)
3. Remember that when a virus or atypical bacterium is identified, co-infection with an unreported typical respiratory bacterium is still possible
4. Diagnostic performance is comparable to laboratory testing with 97.4% overall sensitivity and 99.4% specificity when compared to an FDA-cleared multiplexed respiratory pathogen panel.(40)
5. Results are presented as pathogen detected or not detected. In addition, for Influenza A (including its subtypes) and MERS-CoV only, results may present as equivocal. Please see the BioFire® Respiratory Panel 2.1 *plus* (RP2.1*plus*) brochure (page 19) for more information on the definition of equivocal and the steps to follow with these results.
6. It is common for none, one or multiple (particularly in children) microbes to be detected. There is no evidence to suggest co-infection is associated with more severe illness.
7. False negatives can be due to
   1. Timing of swab sample in relation to illness onset e.g. we know from the COVID pandemic that some patients needed to be tested for SARS-CoV2 multiple times before tests became positive
   2. Poor-quality swab, meaning a microbe being missed
8. False positives can be due to
9. Commensal carriage – some microbes can reside harmlessly in the upper respiratory tract
10. Prolonged post-infection “shedding” can occur e.g. rhinovirus can persist for 28 days

Frequently asked questions

1. The BioFire machine tests for a few coronavirus subtypes (HKU1, NL63, 229E, OC43, MERS-CoV, SARS-CoV-2). What difference does this make?
   - SARS-CoV-2 is the cause of COVID-19 and is a notifiable disease.
   - MERS-CoV causes Middle East Respiratory Syndrome, a severe respiratory illness in travellers from endemic region. You should seek microbiological advice immediately.
   - The remaining coronaviruses (HKU1, NL63, 229E, OC43) cause common cold and sore throat symptoms.
2. The BioFire machine tests for Influenza B and a few Influenza A subtypes (no subtype detected, A H1, A H3, A H1-2009). What difference does this make?
   - Influenza A and B can be managed similarly and are notifiable diseases.
   - In patients with a history of avian flu contact, an Influenza A (no subtype detected) positive result *might* indicate the patient has **avian flu**. Seek microbiological advice.
3. The BioFire machine tests for a few Parainfluenza types (1, 2, 3 and 4). What difference does this make?
   - Types 1 and 2 are most common pathogens associated with croup(41)
   - Type 3 are associated with bronchiolitis and pneumonia in young children(41)
   - Type 4 are less common and not well characterised(41)

Comments/suggestions?

We welcome your comments and suggestions to improve this information sheet. Please email [rapidtest-study@bristol.ac.uk](mailto:rapidtest-study@bristol.ac.uk) with “Interpreting BioFire results” in the subject heading.

# Authored and reviewed by

Alastair Hay (GP and Professor of Primary Care); Peter Muir (Consultant Virologist); Lucy Yardley (Professor of Health Psychology); Matthew Ridd (GP and Professor of Primary Care); Karen Jordan-Coffee (bioMérieux) and Tom Newcombe (bioMérieux).
