# Supplementary material for: Rapid respiratory microbiological point-of-care-testing and antibiotic prescribing in primary care: Protocol for the RAPID-TEST randomised controlled trial
Source: PLoS One. 2024 May 20;19(5):e0302302. doi: 10.1371/journal.pone.0302302 (PMC11104596; doi:10.1371/journal.pone.0302302)
Supplement: S2 File — (PDF) [file pone.0302302.s004.pdf]

Rapid respiratory microbiological point-of-care-testing in primary care: a randomised controlled efficacy trial with internal pilot and qualitative and quantitative investigation of microbial, behavioural and antibiotic mechanisms (the RAPID-TEST RCT)

Protocol version	V3.0 (06 March 2023)
IRAS project ID	299674
REC Reference	North West – Preston (ref: 22/NW/0294)
Sponsor (University of Bristol) reference	2022 - 527
ISRCTN	ISRCTN16039192
Funder (NIHR EME) project number	NIHR131758
NIHR CRN CPMS ID	53931

This protocol has regard for the HRA guidance

Acknowledgement and Disclaimer: This project (NIHR131758) is funded by the Efficacy and Mechanism Evaluation (EME) Programme, an MRC and NIHR partnership. The views expressed in this publication are those of the author(s) and not necessarily those of the MRC, NIHR or the Department of Health and Social Care.

KEY TRIAL CONTACTS

Chief Investigator (CI)	Professor Alastair D Hay, GP and Professor of Primary Care, University of Bristol<br>Tel: 0117 331 4550<br>Email: alastair.hay@bristol.ac.uk
Co-investigators	Professor Joanna Coast, Professor in the Economics of Health and Care, University of Bristol, Email: jo.coast@bristol.ac.uk<br>Professor Chris Metcalfe, Professor of Medical Statistics, University of Bristol, Email: chris.metcalfe@bristol.ac.uk<br>Professor Athene Lane, Professor of Trials Research, University of Bristol, Email: Athene.Lane@bristol.ac.uk<br>Professor Matthew Ridd, Professor of Primary Healthcare, University of Bristol, Email: m.ridd@bristol.ac.uk<br>Dr Paul Mitchell, Senior Lecturer/Research Fellow in Health Economics and Health Policy Analysis, University of Bristol, Email: paul.mitchell@bristol.ac.uk<br>Professor Lucy Yardley, Professor of Health Psychology, University of Bristol, Email: lucy.yardley@bristol.ac.uk<br>Dr Stephen Granier, GP Partner, Whiteladies Medical Group, Email: steve.granier@nhs.net<br>Dr Peter Muir, Consultant Clinical Scientist, Public Health Laboratory Bristol, Email: peter.muir@phe.gov.uk<br>Ms Kathy Eastwood, PPI Member<br>Hayley Dash, PPI Member
Lead Statistician	Professor Chris Metcalfe, Professor of Medical Statistics, University of Bristol, Email: chris.metcalfe@bristol.ac.uk
Trial Manager	Samantha de Jesus, Email: rapidtest-study@bristol.ac.uk
Web address	https://rapidtest.blogs.bristol.ac.uk/

FUNDING AND SUPPORT IN KIND

Funder(s)	Financial and non-financial support given
National Institute for Health and Care Research (NIHR) Efficacy and Mechanism Evaluation (EME) Programme<br>NIHR Evaluation, Trials and Studies Coordinating Centre (NETSCC)<br>University of Southampton, Alpha House, Enterprise Road<br>Southampton SO16 7NS	Grant funding
Bristol Trials Centre (BTC)<br>Population Health Sciences, Bristol Medical School, University of Bristol, 1-5 Whiteladies Road, Bristol, BS8 1NU<br>Telephone: 0117 455 1230<br>Email: btc-mailbox@bristol.ac.uk	Methodological expertise
Dr Adam Taylor<br>Head of Research Governance<br>Research and Enterprise Division, University of Bristol, Second Floor, Augustines Courtyard, Orchard Lane, Bristol, BS1 5DS<br>Telephone: 0117 4556247<br>Email: research-governance@bristol.ac.uk	Sponsor
bioMérieux UK Ltd<br>Grafton Way, Basingstoke, Hampshire, RG22 6HY<br>Tel.: +44 (0) 1256 461881	Provision of advice regarding BioFire specification and use; trouble-shooting problems with BioFire testing equipment (e.g. servicing/providing replacement units); and ensuring accurate description of the BioFire platform in final reports and publications
NHS Bristol, North Somerset and South Gloucestershire ICB<br>360 Bristol, Marlborough Street, Bristol BS1 3NX	Host

Contents

KEY TRIAL CONTACTS 2

FUNDING AND SUPPORT IN KIND 3

GLOSSARY OF ABBREVIATIONS AND DEFINITIONS 7

TRIAL SUMMARY 9

1. BACKGROUND AND RATIONALE 13

1.1 Evidence for Why This Research is Needed Now 14

2. AIMS AND OBECTIVES 15

2.1 Aim 15

2.2 Objectives and Outcomes 15

3. TRIAL DESIGN 19

3.1 Use of Diagnostic Equipment 19

3.2 Internal Pilot Study 19

4. TRIAL SETTING 20

4.1 Recruitment of GP Practices 20

4.2 Trial Promotion 20

5. ELIGIBILITY CRITERIA 20

5.1 Subject Population 20

5.2 Inclusion Criteria 20

5.2.1 GP Practice Criteria 20

5.2.2 Participant Criteria 21

5.3 Exclusion Criteria 21

5.3.1 Participant Criteria 21

5.4 Prior and Concomitant Therapies 21

5.4 Co-enrolment to other research studies 22

6. TRIAL PROCEDURES 22

6.1 Overview of GP Practice Staff Roles 22

6.2 Overview of Trial Assessments 22

6.3 Identification and Screening (Day 1 or up to 2 working days prior to Day 1) 26

6.4 Appointment One (Day 1) 26

6.4.1 Consent 26

6.5 Swab Collection (Post-Appointment One, Day 1) 27

6.6 Randomisation (Day 1) 27

6.6.1 GP POCT^{RM} Test (Intervention) Group 28

6.6.2 GP POCT^{RM} Test (Intervention) Group and No GP POCT^{RM} Test (Control) Group Central Research Laboratory Analysis 28
6.6.3 Study Clinician Views 28
6.7 Appointment Two (Day 1) 29
6.8 Post-Appointment Two (Day 1) 29
6.9 Follow-Up 29
6.10 Optional Interviews and DCE Surveys 29
6.11 Blinding of Allocated Group 29
6.12 Withdrawal from the Trial 30
6.13 Participant Payments and Communication 30
6.14 End of Trial 30
6.15 Trial Stopping Rules 30
7. MIXED-METHODS EVALUATION OF MICROBIAL, BEHAVIOURAL AND ANTIBIOTIC MECHANISMS . 30
7.1 Qualitative Interviews 32
7.1.1 Consent Process for Qualitative Interviews 32
7.1.2 Data Protection and Participant Confidentiality in Relation to the Qualitative Data 32
7.2 Discrete Choice Experiments 33
7.2.1 Consent Process for the DCEs 33
7.2.2 DCE Data Analysis 34
8. DIAGNOSTIC EQUIPMENT 34
8.1 General Information 34
8.2 Distribution and storage of Biofire® FilmArray® Torch 1 Machine and Reagent Pouches ... 34
9. SAFETY 35
9.1 Operational Definitions 35
9.2 Identification of AEs 37
9.3 Classification of (S)AEs 37
9.4 Recording and Reporting Non-Serious AEs Related to the Intervention or Trial Procedures 37
9.5 Recording SAEs 38
9.5.1 Reporting of SUSARs 38
10. STATISTICS AND HEALTH ECONOMICS ANALYSIS 40
10.1 Sample Size Calculation 40
10.1.1 Clinical Sample Size 40
10.1.2 Mechanistic Sample Size 40
10.2 Statistical Analysis 42

10.3 Additional Health Economics Analysis ... 43
11. DATA MANAGEMENT ... 43
11.1 Source Data and Documents ... 43
11.2 Case Report Forms (CRFs) ... 43
11.3 Data Handling and Record Keeping ... 44
11.4 Access to Data ... 44
11.5 Archiving ... 44
11.6 Access to the Final Trial Data Set ... 44
12. TRIAL MANAGEMENT ... 44
12.1 Trial Management Group (TMG) ... 44
12.2 Trial Steering Committee (TSC) ... 44
12.3 Data Monitoring Committee (DMC) ... 45
12.4 Patient and Public Involvement (PPI) ... 45
12.5 Sponsorship, Host and Funding Organisation ... 45
13. MONITORING, AUDIT & INSPECTION ... 45
13.1 Monitoring ... 45
13.2 Protocol Compliance ... 46
13.3 Notification of Serious Breaches to GCP or the protocol ... 46
14. ETHICAL AND REGULATORY CONSIDERATIONS ... 46
14.1 Governance and Legislation ... 46
14.2 Research Ethics Committee (REC) Review and Reports ... 46
14.3 Peer Review ... 47
14.4 Regulatory Compliance ... 47
14.5 Data Quality ... 47
14.6 Financial and Other Competing Interests ... 47
14.7 Indemnity ... 47
15. DISSEMINATION POLICY ... 47
16. SIGNATURE PAGE ... 48
17. AMENDMENT HISTORY ... 49
18. APPENDIX 1 ... 50
19. REFERENCES ... 52

GLOSSARY OF ABBREVIATIONS AND DEFINITIONS

AE	Adverse Event
AMR	Antimicrobial resistance
Antibiotic	Used when referring to antibacterial drugs
Antiviral	Used when referring to antiviral drugs
BTC	Bristol Trials Centre
CI	Chief Investigator
COPD	Chronic Obstructive Pulmonary Disease
CRF	Case Report Form
CRN	Clinical Research Networks
CRP	C-reactive protein
CTIMP	Clinical Trial of an Investigational Medicinal Product
Day 1	Day of randomisation
DCE	Discrete Choice Experiments
DMC	Data Monitoring Committee
EME	Efficacy and Mechanism Evaluation
EudraCT	European Union Drug Regulating Authorities Clinical Trials
GDPR	General Data Protection Regulation
HRA	Health Research Authority
ICB	Integrated Care Board
ICF	Informed Consent Form
ICH-GCP	International Conference on Harmonisation for Good Clinical Practice
ISRCTN	International Standard Randomised Controlled Trials Number
IVD	In Vitro Diagnostic
MCID	Minimum Clinically Important Difference
MHRA	Medicines and Healthcare Products Regulatory Agency
NETSCC	NIHR Evaluation, Trials and Studies Coordinating Centre
NIHR	National Institute for Health and Care Research
NICE	National Institute for Health and Care Excellence
NPV	Negative Predictive Value
PCR	Polymerase Chain Reaction
PCMR	Primary Care Medical Record
PIS	Participant Information Sheet
POCT	Point-of-care-testing
$POCT^{RMs}$	Respiratory microbiological POCT
PPI	Patient and Public Involvement

PPV	Positive Predictive Value
RCT	Randomised Control Trial
REC	Research Ethics Committee
RP2.1 *plus*	Respiratory panel 2.1 *plus* (manufactured by BioFire®)
RSV	Respiratory Syncytial Virus
RTI	Respiratory Tract Infection
SAE	Serious Adverse Event
SARS-CoV-2	Severe Acute Respiratory Syndrome Coronavirus 2
SOP	Standard Operating Procedure
SUSAR	Suspected Unexpected Serious Adverse Reaction
TMF	Trial Master File
TMG	Trial Management Group
TSC	Trial Steering Committee
UHBW	University Hospitals Bristol and Weston
UKHSA	UK Health Security Agency

TRIAL SUMMARY

Trial Title	Rapid respiratory microbiological point-of-care-testing in primary care: a randomised controlled efficacy trial with internal pilot and qualitative and quantitative investigation of microbial, behavioural and antibiotic mechanisms
Short title	RAPID-TEST
Chief Investigator	Professor Alastair D Hay
Sponsor	University of Bristol
Funder	NIHR EME
Aim	To evaluate the use of a rapid respiratory microbiological point-of-care-test (POCT^{RM}) for suspected respiratory tract infections (RTIs) in primary care
Trial Design	Multi-centre, individually randomised controlled trial with internal pilot and mixed-methods investigation of microbial, behavioural and antibiotic mechanisms
Trial Participants	Patients aged ≥12 months presenting to primary care with a Study Clinician suspected RTI where the Study Clinician and/or patient believes antibiotic treatment is, or maybe, necessary
Sample size	514 participants
Number of trial sites	Approximately 16 in total split over 2 waves (approximately 8 sites per wave). Sites are GP practices within the UK.
Intervention	GP POCT^{RM} test
Control	No GP POCT^{RM} test
Inclusion criteria	Eligibility will be assessed by GP practice and participant.<br>**GP practices will be eligible if:**<br>• Served for routine laboratory testing by one of the following four hospitals: Southmead (North Bristol), the Bristol Royal Infirmary, Royal United Hospitals (Bath) and Weston General. GP practices out of this area are eligible if served by a hospital that can accept trial samples within 24 hours and then transfer them to the central laboratory within 24 hours, or are willing to send samples via courier to the central laboratory within 24 hours.<br>• Have a clean, well ventilated area for the Biofire® FilmArray® Torch 1 machine, not in close proximity to sources of strong electromagnetic radiation, and with the following dimensions: depth ≥77cm, width ≥49cm, height ≥30cm and at least 2.6cm between the rear panels and any other surfaces<br>**Participants will be eligible if:**<br>• Aged ≥12 months on the day of presentation to primary care<br>• Presenting to primary care for the first time in this episode, and within 21 days of illness onset, with a Study Clinician suspected acute respiratory infection. Symptoms may include one or more of: sore throat, runny nose, earache, cough, sputum, wheeze or shortness of breath.<br>• Presentation is face-to-face, via telephone or online<br>• Study Clinician diagnosis of an upper or lower RTI such as: acute otitis media, acute sinusitis, acute pharyngitis or tonsillitis, sore throat, acute laryngitis, acute cough, acute bronchitis, chest infection, acute lower RTI, infective exacerbation of chronic lung disease e.g. asthma, chronic obstructive pulmonary disease (COPD), emphysema or bronchiectasis

	• Study Clinician or patient/parent/carer believes antibiotic treatment is, or may be, necessary<br>• Patient/parent/carer willing and able to give informed consent<br>• Patient/parent/carer willing to have a nasal and throat swab taken, or willing and able to self-collect, take and promptly return the swab to the site<br>• Study Clinician and patient/parent/carer willing to wait for the POCT^{RM} result before an antibiotic prescribing decision is made<br>• Laboratory transport pick up for swab samples expected <24 hours e.g. sample is expected to be ready prior to final sample collection on a Friday<br>• Patient/parent/carer willing to complete a Trial Diary and for outcome data to be collected from medical record
Exclusion criteria	• Patient known to have cystic fibrosis<br>• Patient requires hospital admission<br>• Previous participation in the RAPID-TEST trial<br>• Participation in another study of RTI ≤6 weeks prior to randomisation
Primary clinical objective	To investigate whether the use of a rapid POCT^{RM} can reduce same-day antibiotic prescribing for children and adults presenting to primary care with respiratory infections where the Study Clinician and/or patient believes antibiotic treatment is, or may be, necessary
Primary clinical outcome (for full definition see Table 1)	Whether any antibiotic was prescribed (including delayed prescriptions) for a RTI on Day 1
Key secondary clinical objective (for full definition see Table 1)	To investigate whether the use of a rapid POCT^{RM} impacts on patient symptom severity
Summary of other secondary clinical objectives (for full definition see Table 1)	To investigate whether the use of a rapid POCT^{RM} changes:<br>1. Patient confidence in the clinical management of the infection<br>2. Patient symptom duration and severity<br>3. Number of types of subsequent consultations<br>4. The proportion admitted to hospital for respiratory infections ≤28 days<br>5. The quantity of antibiotic and antiviral prescribing ≤28 days<br>6. Antibiotic and antiviral consumption ≤28 days<br>7. Patient intention to consult for similar future illnesses at 2 months
Summary of qualitative objectives (for full definition see Table 2)	1. To explore participants' understanding of the test and the result they were given, and their views of the implications for treatment and future consultations<br>2. To describe the situations in which clinicians most and least value the new microbial knowledge, and how it influences clinical reasoning and participant management
Primary mechanistic objective (for full definition see Table 3)	To determine whether the POCT^{RM} changes Study Clinician beliefs that antibiotics are necessary

Summary of secondary mechanistic objective (for full definition see Table 3)	To describe the effect of $POCT^{RM}$ results on Study Clinician and participant beliefs in the necessity and benefits, of prescribing antibiotics, and confidence in the value of the $POCT^{RM}$
Summary of tertiary mechanistic objectives (for full definition see Table 3)	1. To investigate whether control group Study Clinician antibiotic prescribing is better than chance compared to microbiological testing<br>2. To explore the relationships between baseline symptoms and signs, $POCT^{RM}$ result, antibiotic consumption and participant reported: (i) symptom severity at days 2 to 4; and (ii) duration of moderately bad (or worse) symptoms<br>3. To compare $POCT^{RM}$ results at GP practices with $POCT^{RM}$ results at the central research laboratory (intervention group only), and between $POCT^{RM}$ results at central research laboratory with extended central research laboratory testing
Trial duration	Funding start date: 1 April 2022. Anticipated duration: 38 months (total; subject to change). Anticipated end date: 31 May 2025 (subject to change)

Figure 1: Trial Flowchart

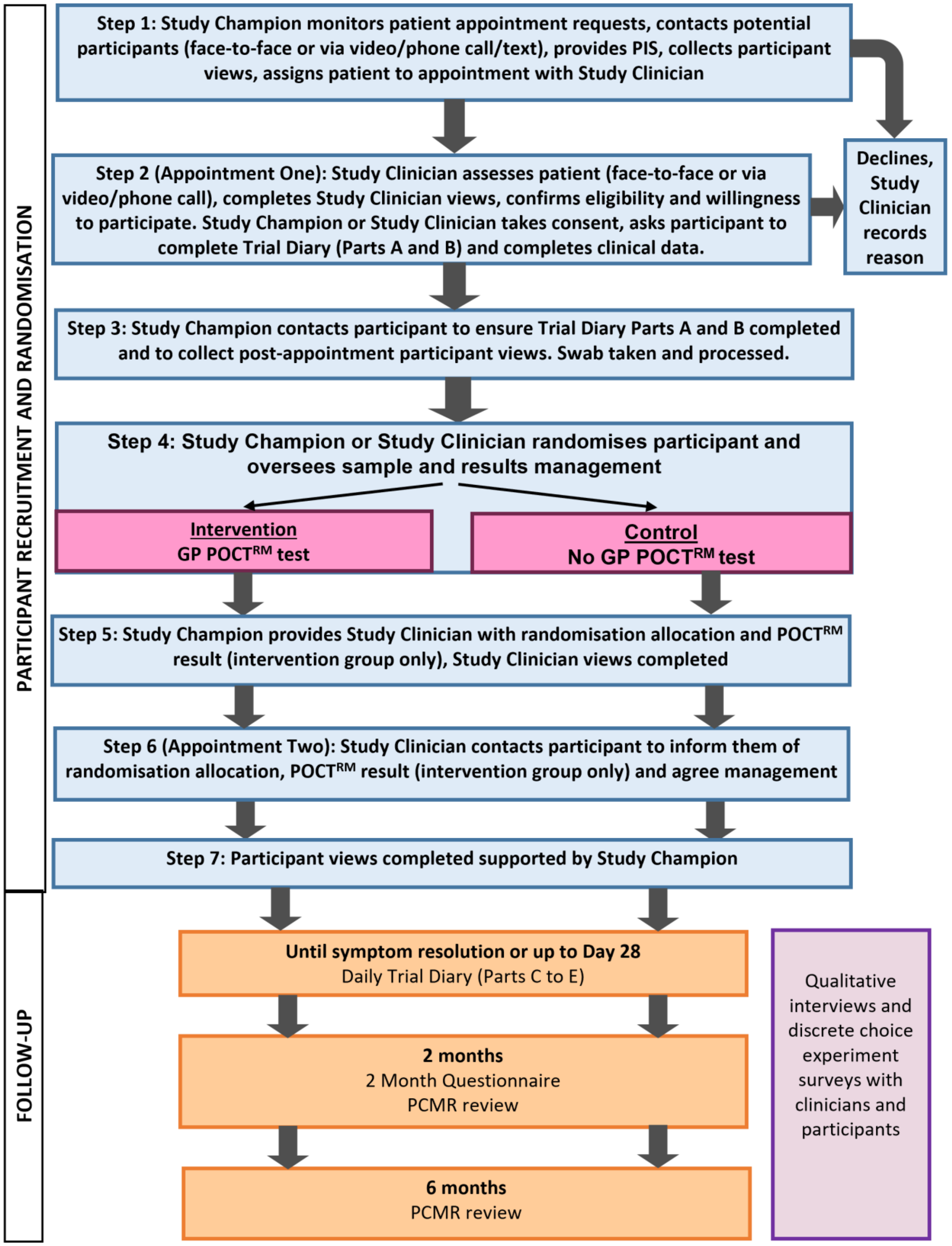

1. BACKGROUND AND RATIONALE

Respiratory tract infections (RTIs) are the most common problem managed by health services internationally.[1] In the UK, GPs and primary care nurses (from here on 'clinicians') treat over 50% of RTIs with antibiotics,[2, 3] with 50% of these considered inappropriate,[4, 5] and despite strong evidence that the majority of patients do not benefit.[6-9] Overprescribing results in unnecessary side effects,[10] depletion of normal flora,[11] encourages patients to seek help for similar future illnesses,[12] and fuels antimicrobial resistance (AMR), [13, 14] regarded as a top 10 threat to global public health.[15] High treatment rates are attributed to clinician uncertainty regarding patients' microbiological diagnosis and clinical prognosis,[16, 17] leading to 'just-in-case' defensive prescribing.[17]

One potential solution, strongly endorsed by Lord Jim O'Neil in 2016,[14] the 2019 UK Government 5-year AMR action plan,[18] and the 2020 Wellcome Trust AMR report,[19] is point-of care-testing (POCT). These 'medical tests at the time and place of patient care',[20] are attractive to primary care because laboratory distances and procedures mean results are not available in time to inform prescribing decisions: typically 24 hours for blood and up to 72 hours for microbiological tests.

The C-reactive protein (CRP) point of care blood test measures the host inflammatory response to infection. They have been shown in randomised controlled trials (RCTs) to reduce antibiotic prescribing for adults with acute lower RTIs by 15%[21] to 22%.[22] However, despite National Institute for Health and Care Excellence (NICE) recommending its use in 2015 for patients with suspected pneumonia,[23] primary care uptake remains stubbornly low. In a recent editorial[24] we speculate that in addition to the 'who pays?' question, this could be because clinicians are unclear how the test works (an elevated CRP does not mean the infection is bacterial[25]). We also observe[24] that CRP effectiveness could be due to nothing more than the low prevalence of elevated CRP in primary care (often not reported) favouring 'no-prescribing' decisions in up to 90% of consultations.

Respiratory microbiological POCTs (POCT^{RMs}) use a polymerase chain reaction (PCR) to detect viruses and bacteria from respiratory tract samples in as little as 45 minutes.[26] They can be considered to be singleplex, duplex or multiplex according to the number of microbes being tested. Until recently, most systems were single/duplex, testing for Influenza A/B and/or Respiratory Syncytial Virus (RSV), but the Severe Acute Respiratory Syndrome Coronavirus 2 (SARS-CoV-2) pandemic has accelerated investment in multiplex POCT^{RM} technology, with the latest equipment able to test for the presence of multiple viruses (including SARS-CoV-2) and bacteria.[27, 28] Importantly, the regulatory requirements for new POCT^{RMs} are significantly lower than for new drugs: manufacturers have only to demonstrate they replicate standard laboratory testing. But prior to adoption, before equipoise is lost, evidence is needed from independent clinical trials to show safety and efficacy and later clinical and cost-effectiveness.

POCT^{RMs} are being increasingly used in hospitals to assist with 'bubbling' e.g. patients with/out SARS-CoV-2 and RSV and antibiotic prescribing decisions. Multiplex POCT^{RMs} have been shown in two Emergency Department RCTs[29, 30] and two observational ward-based before/after comparisons[31, 32] to reduce hospital admission times and durations of antibiotic courses, with only one showing reduced overall antibiotic prescribing.[30] None reported patient symptom outcomes.

To ensure a complete understanding of the primary care POCT^{RM} literature we conducted a systematic review, searching Medline, Embase and Cochrane (any language) between January 2000 and December 2020. We found a systematic review (search to September 2017)[33] reporting ten studies all investigating the effects of duplex POCT^{RMs} testing for Influenza A and B, and/or RSV, but none reporting the use of multiplex POCT^{RMs}. None reported if the POCT^{RM} improved clinical outcomes, with most reporting technical aspects such as turn-around time and test accuracy.

We found two further observational studies not reported in the systematic review. The first, conducted in six UK GP practices[34] showed a singleplex Influenza POCT^{RM} was acceptable to clinicians and had potential to reduce antibiotic (and increase antiviral) prescribing. The other, conducted in a single Dutch GP practice used a multiplex POCT^{RM} (the mariPOC® Respi, providing results for nine respiratory viruses in two hours),[35] showed the test was acceptable to clinicians and found clinically useful positive and negative predictive

values for Influenza A and B, and RSV (PPV range 87% to 100%, NPV 94 to 97%). Both studies concluded RCTs are needed to demonstrate impact on antibiotic prescribing and patient outcomes.

We conducted a mixed-methods feasibility study to investigate the use and acceptability of a multiplex POCT^{RM}.[36] Twenty clinicians from four GP practices used the POCT^{RM} in 93 participants during a single winter. It was used in around one third of potentially eligible patients, 72% of swabs were processed in <4 hours, 90% in <24 hours with a median set-up time of 2.7 minutes. Tests detected ≥1 virus in 58% of samples, no pathogen in 37%, atypical bacteria in 2%, while 3% were inconclusive. Antibiotics were prescribed to 35% of patients with no pathogen detected and 25% with a virus. Post-test clinical diagnoses changed in 20%, and diagnostic certainty increased ($p=0.02$), more so when the test detected a pathogen than not ($p<0.001$). Clinicians predicted decreased antibiotic benefit post-test ($p=0.02$). The 22 semi-structured qualitative interviews with healthcare professionals showed the test was easy to use and well-liked but limited by time to result and the absence of testing for typical respiratory bacteria.

None of the studies considered or evaluated the short and long-term effects of POCT^{RM} testing on clinician and patient beliefs and behaviour, and how these changes may mediate the effect of POCT^{RM} testing on prescribing.

We believe this is the first RCT proposed in primary care to investigate: if a multiplex POCT^{RM} can reduce antibiotic prescribing; its effects on patient outcomes; and use of mixed-methods to directly examine hypothesised behaviour change mechanisms.

1.1 Evidence for Why This Research is Needed Now

The 'holy grail' of antimicrobial stewardship is to ensure the minority of patients needing treatment are given the shortest course of the narrowest spectrum antibiotic possible, while preventing unnecessary exposure among the majority unlikely to benefit, i.e. precision prescribing. The vast majority of stewardship interventions currently available aim to reduce overall prescribing,[37] with only a handful of validated tools available to support precision prescribing in primary care.[38, 39]

Given that a significant proportion of RTIs managed in primary care are viral,[40-42] it is plausible that a POCT^{RM} providing accurate results quickly could improve prescribing. Accuracy depends on both the quality of the sample and equipment test performance. Lower respiratory tract sampling is not relevant for many community-acquired RTIs and, where it is, may not be practical. Throat and nose sampling is universally available, acceptable,[42] and equivalent to nasopharyngeal samples,[43] even when self-collected by patients.[43, 44] However, the main disadvantage of throat/nose sampling is that the common bacteria causing respiratory infections (*S. pneumoniae*, *M. catarrhalis* and *H. influenzae*) also reside harmlessly in the upper respiratory tract i.e. the nose and throat are not sterile. The resulting false positive results mean manufacturers do not include these microbes in upper respiratory tract panels. Respiratory viruses may also harmlessly reside in the upper respiratory tract,[45] but less commonly. While there is currently no way to distinguish 'commensal carriage' from infection at the time of testing, patients with infection would be expected to experience worse outcomes, so measuring patients' subsequent illness trajectory and response to antibiotics is important.

There is also evidence that microbiologically informed antibiotic prescribing could improve patient outcomes. Well-conducted RCTs consistently demonstrate clinically unimportant improvements in symptom severity and duration, [6-9] suggesting there could be (as yet) unidentified sub-group/s experiencing clinically important benefits, and two studies suggest that patients from whom bacteria are isolated and given antibiotics have shorter illnesses compared to those not given antibiotics.[46, 47]

Moreover, our research shows that the detection of some upper respiratory tract microbes is associated with more prolonged illness[48] and more severe symptoms at days 2 to 4,[49 50] suggesting that detecting microbes could help manage patient expectations for subsequent illness trajectory and could help identify subgroups more (and less) likely to benefit from antibiotic (antiviral) treatment.

We considered multiplex systems would provide more clinical certainty than single or duplex POCT^{RMs} systems. We are aware of three multiplex POCT^{RMs} manufacturers: bioMérieux (Biofire® Respiratory 2.1), GenMark (ePlex new respiratory pathogen) and Qiagen (QIAstat-Dx Respiratory panel). The bioMérieux

system will be used in the RAPID-TEST trial because of its: high test accuracy, established technology (since 2011), fastest time-to-result (45 minutes compared to 1-2 hours), lowest price per test and because we used the same system in our feasibility study.[36]

There is currently insufficient evidence regarding the safety, efficacy, or mechanism of action, let alone clinical and cost effectiveness, to recommend use of POCT^{RMs} in primary care. Efficacy studies are urgently needed to address these issues using randomised controlled designs, as well as qualitative methods to understand clinician and patients' perceptions of the tests. The RAPID-TEST trial will show whether using multiplex POCT^{RMs} in primary care could safely reduce the unnecessary use of antibiotics, and the beliefs that sustain unnecessary use, thereby reducing the public AMR risk. We will also show whether POCT^{RM} use affects patient outcomes, antiviral prescribing, re-consultations and future consultations for RTIs. Results will inform the design of future pragmatic studies to investigate clinical and cost-effectiveness of POCT^{RMs} in primary care.

2. AIMS AND OBECTIVES

2.1 Aim

To evaluate the use of a rapid POCT^{RM} for suspected RTIs in primary care.

2.2 Objectives and Outcomes

All objectives and their associated outcomes are listed in Tables 1 to 3.

Table 1: Clinical Objectives and Outcome Measures

**Objective**	**Outcome Measure**	**Source and published reference (where applicable)**
Primary clinical objective		
To investigate whether the use of a rapid POCT^{RM} can reduce same-day antibiotic prescribing for children and adults presenting to primary care with respiratory infections where the Study Clinician and/or patient believes antibiotic treatment is, or may be, necessary.	Antibiotic prescribing (including delayed prescribing) for a RTI at Appointment Two (Step 6, Figure 1)	Study Clinician/Study Champion completed REDCap data
Key secondary clinical objective		
To investigate whether the use of a rapid POCT^{RM} changes participant reported symptom severity on days 2 to 4[A]	Mean symptom severity on Days 2 to 4[A]	Trial Diary[51] completed daily by participant
Other secondary clinical objectives		
To investigate whether the use of a rapid POCT^{RM} changes:		
1. Participant (or parent/carer if the participant is <16 years) confidence in the clinical management of the infection	See Appendix 1 (Participant Views question 5)	Participant views at Step 7 (Figure 1). For published references see Appendix 1.

Objective	Outcome Measure	Source and published reference (where applicable)
2. Duration of moderately bad (or worse) symptoms	Last day on which any symptom was recorded as “moderately bad” or worse	Trial Diary[51] completed daily by participant
3. Time to return to usual activities	Length of time to return to usual activities. This will be defined as per the ALICE trial[52]:<br>• Participants ≥16 years as the last day on which they mark “Unable to do usual activities” AND “Fever” as no more than “slight problem”.<br>• Children <16 year as the last day on which they mark “Child not themselves/more clingy then usual” AND “Fever” as no more than “slight problem”	Trial Diary[51] completed daily by participant
4. Overall symptom duration	Last day prior to all symptoms recorded as zero for two consecutive days	Trial Diary[51] completed daily by participant
5. The proportion with new or worsening symptoms ≤28 days	Any new symptoms or worsening of pre-existing symptoms	Trial Diary[51] completed daily by participant
6. The proportion consulting after Appointment Two and ≤28 days for RTI (with/out evidence of it being for the same illness, with/out evidence of the same illness having deteriorated)	Documentation of any RTI related consultation (in and out of normal office hours) after Appointment Two and ≤28 days, whether for the same symptoms /illness as the recruitment consultation with/out documentation that symptoms are getting worse	Retrospective collection from PCMR at least 2 months after recruitment
7. The proportion with hospital admissions for respiratory infections ≤28 days	Evidence of any hospital admission for a respiratory infection in the medical records ≤28 days	Retrospective collection from PCMR at least 2 months after recruitment
8. The quantity and timing of antibiotic and antiviral prescribing after Appointment Two and ≤28 days	Name, strength, frequency and quantity of antibiotics and antivirals prescribed after Appointment Two and ≤28 days	Retrospective collection from PCMR at least 2 months after recruitment
9. Number of days on which antibiotics and antivirals are consumed ≤28 days	Name of antibiotic and antiviral, and number of times per day the medicine was consumed	Trial Diary[51] completed daily by participant

Objective	Outcome Measure	Source and published reference (where applicable)
10. Participant (or parent/carer if the participant is <16 years) intention to consult for similar future illnesses at 2 months	See Appendix 1 (Participant views questions 7 and 8)	Questionnaire completed by participant 2 months post-randomisation
11. The number of respiratory infection consultations >28 days and ≤6 months	Documentation of any consultation (in and out of normal office hours) ≤6 months for a RTI	Retrospective collection from PCMR at 6 months post-randomisation.

[A] Day 1 is the day of randomisation

Table 2: Qualitative and Discrete Choice Experiment (DCE) Objectives and Outcomes

Objective	Outcome Measure	Source and published reference (where applicable)
1. To explore participants' (or parents'/carers' if the participant is <16 years) understanding of the test and the result they were given, and their views of the implications for treatment and future consultations	Participant responses to qualitative questions	Participant interview
2. To explore the trade-offs participants (or parents/carers if the participant is <16 years) make in choosing to visit the GP with respiratory infection symptoms and the extent POCT^{RMs} might increase or decrease help seeking behaviour	Discrete choice experiment (DCE) survey to be developed based on qualitative participant interviews	Participant DCE survey
3. To describe the situations in which clinicians most and least value the new microbial knowledge, and how it influences clinical reasoning and participant management	Clinician responses to qualitative questions	Clinician interview
4. To explore the trade-offs clinicians make about whether and when to use the POCT^{RM}	DCE survey to be developed based on qualitative clinician interviews	Clinician DCE survey

Table 3: Mechanistic Objectives and Outcomes

Objective	Outcome Measure	Source and published reference (where applicable)
Primary mechanistic objective<br>To determine whether there are overall (GP POCT^{RM} test vs. No GP POCT^{RM} test) and differential (virus detected vs. not detected) effects with respect to reducing	See Appendix 1 (Study Clinician views question 1)	Study Clinician views at Steps 2 and 5 (Figure 1) and POCT^{RM} result. For

Objective	Outcome Measure	Source and published reference (where applicable)
the number of participants for whom the Study Clinician believes antibiotics are necessary (as a mediator of the primary clinical outcome)		published references see Appendix 1.
Secondary mechanistic objective<br>To describe the effect of POCT^{RM} results on Study Clinician and participant (or parent/carer if the participant is <16 years) beliefs in the necessity, and benefits, of prescribing antibiotics for the respiratory infection, and confidence in the value of the POCT^{RM} to guide the prescribing decision and explore relationships of Study Clinician and participant (or parent/carer if the participant is <16 years) beliefs, attitudes and intentions with antibiotic prescribing and consumption	See Appendix 1 (Study Clinician views questions 2 to 7; and Participant views questions 1 to 5).	Study Clinician views at Steps 2 and 5, Participant views at Steps 1, 3 and 7 (Figure 1). For published references see Appendix 1.
Tertiary mechanistic objectives		
1. To investigate the extent to which control group Study Clinician antibiotic prescribing is better than chance with respect to 'virus detected' and 'no virus detected' results	Antibiotic prescribing (including delayed prescribing) for a RTI at Appointment Two (Step 6, Figure 1)<br>Central research laboratory POCT^{RM} result	Study Clinician/Study Champion completed REDCap data<br>Central research laboratory results
2. To explore the relationships between baseline symptoms and signs, POCT^{RM} result, antibiotic consumption and participant reported: (i) mean symptom severity at days 2 to 4[A]; (ii) duration of moderately bad (or worse) symptoms	Baseline symptoms and signs<br>POCT^{RM} result<br>Any antibiotic consumption at ≤7 days<br>Mean symptom severity at Days 2 to 4[A]<br>Duration of moderately bad (or worse) symptoms	Baseline CRF<br>POCT^{RM} result<br>Trial Diary[51] completed daily by participant
3. To assess agreement between POCT^{RM} results at GP practices vs. POCT^{RM} results at the central research laboratory (intervention group only)	POCT^{RM} results at GP practices vs. POCT^{RM} results at central research laboratory	POCT^{RM} results from GP practice and from central research laboratory
4. To assess agreement between POCT^{RM} results at central research laboratory vs. extended laboratory respiratory virus and bacteria testing	POCT^{RM} results at central research laboratory vs. extended laboratory testing	Central research laboratory results
5. To assess the relationship between weekly EQ-5D measures and symptom severity scores	EQ-5D-5L or EQ-5D-Y and mean daily symptom severity	EQ-5D-5L[53] (EQ-5D-Y for children aged ≥4 to <16 years) completed weekly

Objective	Outcome Measure	Source and published reference (where applicable)
		and Trial Diary[51] completed daily by participant

[A] Day 1 is the day of randomisation

3. TRIAL DESIGN

This is a multi-centre, individually randomised controlled trial with internal pilot and mixed-methods investigation of microbial, behavioural and antibiotic mechanisms.

The trial duration is expected to be 38 months in total from the funding start date to the final report being submitted although this is subject to change.

3.1 Use of Diagnostic Equipment

Nasal and throat swabs will be taken from participants using standard swab kits provided and the swab samples will be analysed using the BioFire® Respiratory panel 2.1 *plus* (RP2.1 *plus*) reagent pouches which will be run on the Biofire® FilmArray® Torch 1 system (see section 8).[26] The manufacturer's instructions for the BioFire RP2.1 *plus* reagent pouch and FilmArray Torch System are available separately.

As the BioFire RP2.1 *plus* reagent pouch and FilmArray Torch System are CE-marked for the purpose that is under investigation in the RAPID-TEST trial, a letter of no objection from the Medicines and Healthcare Products Regulatory Agency (MHRA) is not required.

3.2 Internal Pilot Study

Following set-up, an internal pilot study will be conducted for approximately 7 months over the Winter 1 period. Trial viability will be assessed against the criteria shown in Table 4 which aims to confirm GP and participant recruitment, collection of data for the primary outcome and whether Study Clinicians are waiting for the POCT^{RM} results before prescribing further treatment.

Table 4: Internal pilot study to main trial stop/go criteria

Progression to trial if all criteria are met	***Modify trial procedures if any criteria are met***	***Stop trial if any criteria are met***
≥6 GP practices set up and using POCT^{RM}	≥3 and <6 GP practices set up and using POCT^{RM}	<3 GP practices set up and using POCT^{RM}
≥128 (≥80% of target*) participants randomised	≥80 and <128 (≥50% and <80% of target*) participants randomised	<80 (<50% of target*) participants randomised
≥90% of randomised participants with primary outcome	≥80% and <90% of randomised participants with primary outcome	<80% of randomised participants with primary outcome
≥70% participants for whom Study Clinicians waited for POCT^{RM} result before deciding if antibiotic treatment was necessary (intervention group only)	≥50% and <70% participants for whom Study Clinicians waited for POCT^{RM} result before deciding if antibiotic treatment was necessary (intervention group only)	<50% participants for whom Study Clinicians waited for POCT^{RM} result before deciding if antibiotic treatment was necessary (intervention group only)

* of Winter 1 target = 160 participants randomised

4. TRIAL SETTING

The trial will mostly be run at GP practices served for routine laboratory testing by one of the following four hospitals: Southmead (North Bristol), the Bristol Royal Infirmary, Royal United Hospitals (Bath) and Weston General. This is because part of the swab sample will be sent to a central research laboratory (Severn Infections Sciences, Southmead Hospital) using routine laboratory transport.

GP practices out of this area will also be able to take part, if they are served by a hospital that can accept trial samples and then transfer them to the central laboratory within 24 hours of receipt or are willing to send samples via courier to the central laboratory.

It is planned to set up approximately 8 GP practices (sites) during Wave 1 which will recruit for the Winter 1 period (the internal pilot study) followed by part of a Summer 1 period. Those initial 8 sites will gradually close during the Summer 1 period and a further 8 sites (approximately) will then be set up as Wave 2 which will start part way through Summer 1 and continue for Winter 2 and Summer 2.

As the trial procedures are considered to be relatively complex, GP practices invited to take part will ideally have experience of Clinical Trials of an Investigational Medicinal Product (CTIMP) and/or on-site research capacity.

4.1 Recruitment of GP Practices

We will utilise our previous knowledge and experience of local research active GP practices and liaise with the local Clinical Research Network (West of England).

4.2 Trial Promotion

Participating GP practices will display posters in waiting rooms and may put information about the trial on practice websites.

The trial website will contain the Participant Information Sheets (PISs) and contact details. A short animation based on the PIS may be produced. A trial Twitter account may be used to raise awareness of the trial and for information purposes only.

5. ELIGIBILITY CRITERIA

5.1 Subject Population

Patients aged ≥12 months presenting to primary care with a Study Clinician suspected RTI where the Study Clinician and/or patient believes antibiotic treatment is, or may be, necessary. Patients can present to their GP Practice face-to-face, via telephone or via on online appointment.

NB: Wherever carer is referred to within the protocol or trial documents, this means the legal guardian of a participant aged under 16 years.

5.2 Inclusion Criteria

5.2.1 GP Practice Criteria

- Served for routine laboratory testing by one of the following four hospitals: Southmead (North Bristol), the Bristol Royal Infirmary, Royal United Hospitals (Bath) and Weston General. GP practices out of this area are eligible if served by a hospital that can accept trial samples and then transfer them to the central laboratory within 24 hours of receipt (participant recruitment can only take place Monday to Thursday due to the additional time involved in samples reaching the central laboratory) or are willing to send samples via courier to the central laboratory.
- A clean, well-ventilated area for the Biofire® FilmArray® Torch 1 machine, not in close proximity to sources of strong electromagnetic radiation (e.g. ultraviolet, X-Rays and Gamma rays), and with the following dimensions: depth ≥77cm, width ≥49cm, height ≥30cm and at least 2.6cm between the rear panels and any other surfaces

5.2.2 Participant Criteria

- Aged ≥12 months on the day of presentation to primary care
- Presenting to primary care for the first time in this episode, and within 21 days of illness onset, with a Study Clinician suspected acute respiratory infection. Symptoms may include one or more of:
    - Sore throat
    - Runny nose
    - Earache
    - Cough
    - Sputum
    - Wheeze
    - Shortness of breath
- Study Clinician diagnoses of an upper or lower RTI such as:
    - Acute otitis media
    - Acute sinusitis
    - Acute pharyngitis or tonsillitis
    - Sore throat
    - Acute laryngitis
    - Acute cough
    - Acute bronchitis
    - Chest infection
    - Acute lower RTI
    - Infective exacerbation of chronic lung disease e.g. asthma, chronic obstructive pulmonary disease (COPD), emphysema or bronchiectasis
- Study Clinician or patient/parent/carer believes antibiotic treatment is, or may be, necessary (either Study Clinician or patient/parent/carer must answer "strongly agree", "agree" or "neither agree nor disagree" to question 1 from the Study Clinician views and question 1 from Participants Views detailed in Appendix 1)
- Patient/parent/carer willing and able to give informed consent
- Patient/parent/carer willing to have a nasal and throat swab taken, or willing and able to collect, self-take and promptly return the swab to the site
- Study Clinician and patient/parent/carer willing to wait for the POCT^{RM} result before an antibiotic prescribing decision is made
- Laboratory transport pick up for samples expected <24 hours e.g. sample is expected to be ready prior to final sample collection on a Friday
- Patient/parent/carer willing to complete Trial Diary and for outcome data to be collected from medical record

5.3 Exclusion Criteria

5.3.1 Participant Criteria

- Patient known to have cystic fibrosis
- Patient requires hospital admission
- Previous participation in the current RAPID-TEST trial
- Participation in another study of RTI ≤6 weeks prior to randomisation

5.4 Prior and Concomitant Therapies

Patients already being treated with antibiotics or antivirals (for any indication) will be eligible as long as the Study Clinician suspects a new (or ongoing) RTI, and the Study Clinician and/or patient/parent/carer believe further antibiotic treatment is, or may be, necessary.

5.4 Co-enrolment to other research studies

Co-enrolment to other research studies will be considered on a case-by-case basis. Participants taking part in other observational studies or interventional trials not involving RTIs could be co-enrolled in RAPID-TEST as long as the intervention has finished and is not expected to affect participant reported symptoms.

6. TRIAL PROCEDURES

6.1 Overview of GP Practice Staff Roles

Each GP practice will identify at least one Study Clinician who is usually responsible for the care of patients with respiratory infections e.g. a GP, practice nurse, pharmacist, paramedic, who will recruit participants into the trial.

In addition, each GP practice will identify at least one Study Champion who is a different member of the practice team such as a receptionist, healthcare assistant, medical student, pharmacist, paramedic, practice nurse or manager. The Study Champion must work at least one session on the same day as the Study Clinician. Study Clinicians may also act as Study Champions for patients they are not clinically assessing. They will be fully trained in trial procedures and responsible for:

- Screening for potentially eligible patients (Figure 1, Step 1)
- Assigning these patients for assessment by the Study Clinician (Figure 1, Step 2)
- Ensuring Study Clinicians (Figure 1, Step 2) and participants (Figure 1, Step 3) complete swab and data collection before proceeding to Step 4 (Figure 1)
- Randomising participants and ensuring swab samples are processed accordingly (Figure 1, Step 4)
- Providing the Study Clinicians with the randomisation outcome and for those allocated to the intervention, providing the Study Clinician with the POCT^{RM} result (Figure 1, Step 5)
- Ensuring Study Clinicians and participants complete final data collection elements (Figure 1, Steps 5 and 7)

Where the Study Champion is not trained in processing the clinical swabs taken from participants for the trial, at least one further member of staff must be trained to perform this task.

The central trial team will support GP Practice staff with trial procedures.

At any step, GP staff may contact participants face-to-face, by phone, text or other approved communication methods at the GP practice e.g. approved in-house text messaging software such as AccuRX.

6.2 Overview of Trial Assessments

A total of 514 participants will be recruited via GP practices. The trial assessments and procedures scheduled at various timepoints are shown in Table 5 and each participant will be involved in the trial for 2 months with a review of their Primary Care Medical Record (PCMR) at 6 months.

To summarise, participants will undergo:

- Identification and screening on Day 1 or up to 2 working days prior to Day 1 (see section 6.3)
- Appointment One and swab collection on Day 1 (see sections 6.4 and 6.5)
- Randomisation on Day 1 (see section 6.6)
- Appointment Two and Post-Appointment Two on Day 1 (sees section 6.7 and 6.8)
- Follow-up until symptom resolution or 28 days post-randomisation (whichever comes first) and at 2 months post-randomisation (see section 6.9)

Table 5: Schedule of Assessments

See Figure 1 for information on each of the steps referred to.

Timepoint (→)	Pre-randomisation				Step 4: Randomisation (Day 1)	Post-randomisation						
**Trial assessments (↓)**	**Site set-up**	**Step 1: Identification /Screening (Day 1 or up to 2 working days prior to Day 1)**	**Step 2: Appointment One (Day 1)**	**Step 3: Post-Appointment One (Day 1)**		**[A]Step 5: Post-randomisation**	**Step 6: Appointment Two (Day 1)**	**Step 7: Post-Appointment Two (Day 1)**	**Day 1 and up to Day 28**	**After completion of Trial Diary**	**2 Months**	**6 Months**
Site and Study Clinician baseline data	●											
Screening		●										
[B]Participant views		●										
Study Clinician views			●			●						
Eligibility assessment			●									
Informed consent			●									
Demographics and clinical data			●									
[C]Trial Diary			●						●			
[B]Post-appointment participant views				●				●				
Nasal and throat swab taken				●								
Randomise participant					●							
Study Champion provides swab results to Study Clinician (for						●						

Timepoint (→)	Pre-randomisation				Step 4: Randomisation (Day 1)	Post-randomisation						
**Trial assessments (↓)**	**Site set-up**	**Step 1: Identification /Screening (Day 1 or up to 2 working days prior to Day 1)**	**Step 2: Appointment One (Day 1)**	**Step 3: Post-Appointment One (Day 1)**		**[A]Step 5: Post-randomisation**	**Step 6: Appointment Two (Day 1)**	**Step 7: Post-Appointment Two (Day 1)**	**Day 1 and up to Day 28**	**After completion of Trial Diary**	**2 Months**	**6 Months**
participants in GP POCT^{RM} test group only)												
Study Clinician provides swab results to participant (for participants in GP POCT^{RM} test group only)							●					
Agree antibiotic treatment							●					
[B]Optional Participant Interview										●		
[B]2 Month Questionnaire											●	
[D]PCMR review											●	●
Optional Clinician Interview	Throughout trial from the time the site opens and recruitment has started											
[B]Optional Participant DCE Survey	Throughout trial after all the participant interviews are completed and participant has completed their Trial Diary											
[E]Optional Clinician DCE Survey	Throughout trial after all the clinician interviews are completed											

[A]Where the participant is in the No GP POCT^{RM} test group, this data can be completed immediately after randomisation. Where the participant is in the GP POCT^{RM} test group, the Study Clinician must wait until the POCT^{RM} results are available before completing this data.

[B]For participants <16 years on Day 1, their parent/carer will be asked to provide parental views on behalf of their child for the participant views on Day 1, the 2 Month Questionnaire and the optional interview and DCE survey.
[C]Trial Diary needs to be completed at baseline by the participant (or their parent/carer for participants <16 years) and then daily from Day 1 until their symptoms resolve or up to Day 28, whichever comes first. The EQ-5D will be completed at baseline and then weekly from Day 1 up to the end of the week that symptoms resolve.
[D]At 2 months, this will be data collection of GP consultations and hospital admissions for respiratory tract infections and antibiotic or antiviral prescribing up to Day 28. At 6 months, this will be data collection of GP consultations for respiratory tract infections occurring between Day 29 and up to 6 months.
[E]Study Clinicians involved in the trial and other clinicians recruited through the West of England Clinical Research Network will be invited to complete a Clinician DCE Survey.

6.3 Identification and Screening (Day 1 or up to 2 working days prior to Day 1)

Figure 1, Step 1

The Study Champion monitors requests for appointments from potentially eligible patients and will contact them either face-to-face or remotely (via a video/phone call/text). For participants <16 years, a parent/carer will also need to be present, it will not be possible for participants <16 years old to join the trial without parent/carer consent. Patients or parents/carers will be provided a copy of the relevant Participant Information Sheet (PIS) or Booklet (see Table 6) (either as a paper copy or electronic version) and allow them sufficient time to read it. The Study Champion will explain what is involved in the trial and answer any questions. As part of screening, the Study Champion will ask the patient (or parent/carer if the patient is <16 years) if they are happy to answer three short questions giving their views on antibiotics (see Appendix 1, Participant Views questions 1, 2 and 3). It is important these questions are asked before Appointment One since interaction with the Study Clinician could change participant views. The Study Champion will then assign the patient to an appointment with a Study Clinician.

6.4 Appointment One (Day 1)

Figure 1, Step 2

Appointment One will be held either face-to-face or remotely (via a video/phone call). The Study Clinician will assess the patient as per usual clinical care up to (but not including) making the final antibiotic treatment decision which must be deferred until Step 5. The Study Clinician will complete their Study Clinician views (see Appendix 1, Study Clinician Views questions 1 to 5) and then determine eligibility and willingness to take part. The Study Champion or the Study Clinician will take consent (see below) and collect demographic and clinical data. They will also provide the participant or parent/carer with the Trial Diary, either online or in paper format according to participant preference and ask the patient to complete Part A on baseline symptoms and Part B on the EQ-5D. If the patient (or parent/carer if the patient is <16 years) declines to take part in the trial, the reason for this (if provided) will be collected along with brief non-identifiable data including age Patients who decline will be returned to standard care.

6.4.1 Consent

During Appointment One, the patient will be given the opportunity to understand the nature, significance, implications and risks of the trial in order for them to make an informed decision about whether or not to take part. If the patient wishes to proceed, informed consent will be taken using an e-consent mechanism (where e-consent means the use of electronic methods for seeking, confirming and documenting informed consent and is the preferred option) which will follow Health Research Authority (HRA) and MHRA guidance[54] or if this is not possible paper-based and verbal consent will be considered (see below). The relevant Informed Consent Form (ICF) and Assent Form if required (see Table 6) will be presented in an electronic format and a simple electronic signature will be used to record consent. A copy of the e-consent form will be made available to the participant via email or post.

If verbal consent is taken, it will be documented by the researcher taking consent on the appropriate ICF (and Assent Form if required). The researcher taking consent will record the participant's (or parent's/carer's) responses in each box on the ICF (and Assent Form if required) in the presence of a witness (any member of staff on duty who is not connected with the trial i.e. is not on the Delegation Log) who should also countersign the form. A copy of the completed ICF (and Assent Form if required) will then be sent to the participant (or parent/carer) in the post.

Four copies of the ICF (and Assent Form if required) will be needed: (1) to be provided to the participant along with a copy of the relevant Participant information Sheet; (2) to be filed along with a copy of the relevant Participant Information Sheet in the GP medical records; (3) to be filed in the ISF (4) to be filed in the central TMF.

In addition to completing the consent form, sites should record consent has been given for the trial in the participant's medical records.

To cover the broad age range of participants, a number of different PISs, ICFs, a Children's Booklet, and an Assent Form will be available as shown in Table 6 along with a guide showing which ages these are suitable for. Participants aged 12 - 15 years will need to give their assent plus their parent/carer will need to give consent for the child to be able to take part in the trial. For participants <12 years, their parent/carer will need to give consent for the child to take part. A Summary Leaflet will also be available to supplement the PISs.

Table 6: Age appropriate versions of information sheets and consent forms

**Participant Age**	**Documents to be provided**	
	**PIS/Children's Booklet**	**ICF/Assent Form**
≤12 years	RAPID-TEST Children's Booklet (for the participant)<br>RAPID-TEST Parent/Carer PIS (for the participant's parent or carer)<br>RAPID-TEST Summary Leaflet	RAPID-TEST Parent/Carer ICF (for the participant's parent or carer)
12 – 15 years	RAPID-TEST PIS 12-15 years (for the participant)<br>RAPID-TEST Parent/Carer PIS (for the participant's parent or carer)<br>RAPID-TEST Summary Leaflet	RAPID-TEST Assent Form 12 – 15 years (for the participant)<br>RAPID-TEST Parent/Carer ICF (for the participant's parent or carer)
≥16 years	RAPID-TEST PIS<br>RAPID-TEST Summary Leaflet	RAPID-TEST ICF

6.5 Swab Collection (Post-Appointment One, Day 1)

Figure 1, Step 3

The Study Champion or Study Clinician will support the participant to complete their baseline symptoms and EQ-5D in the Trial Diary (Parts A and B) and provide details of how to start filling in the rest of the Trial Diary (Parts C-E).

The Study Champion will also collect post-appointment participant views (see Appendix 1, Participant Views questions 1 to 5). If the participant is <16 years, their parent/carer will complete the post-appointment participant views. If the participant is <16 years, their parent/carer will complete the Trial Diary with, or on behalf of, the participant.

The Study Champion or Study Clinician or another trained member of staff will take a nasal and throat swab from participants who are attending the GP practice. If the participant prefers to take their own swab, or Appointment One is held remotely, the participant (or a member of their household) will be asked to visit the GP practice to collect a trial pack, containing a swab collection kit and instructions on how to take the swab. The swab can be taken and returned at the same visit. If the participants prefers to take the kit home, they will be asked to take and return the swab on the same day as the kit was collected i.e. on Day 1.

Swabs from all participants will be processed according to the Laboratory Manual and while wearing appropriate PPE in line with current guidelines.

6.6 Randomisation (Day 1)

Figure 1, Step 4

Participants will be individually randomised 1:1 to intervention (GP POCT^{RM} test) or control (No GP POCT^{RM} test) using an internet-based randomisation system developed and maintained by Sealed Envelope™.

Randomisation will be stratified by age (<16 years vs. ≥16 years) and chronic lung disease (present vs. absent). It will not be possible to blind the allocated randomisation group since the subsequent processes for each group differ.

The Study Champion or Clinician randomises participants and immediately informs (if necessary) the Study Clinician which group the participant has been allocated to.

6.6.1 GP POCT^{RM} Test (Intervention) Group

A portion of the swab sample from participants in the GP POCT^{RM} test group will be analysed immediately by the Study Champion or Study Clinician using the BioFire® FilmArray® Torch 1 in conjunction with BioFire® RP2.1 *plus* reagent pouches according to the manufacturer's instructions in the GP practice. The time for processing one swab to results being available is approximately 1 hour, assuming the Torch 1 machine is not already in use. The results will indicate presence or absence of 23 upper respiratory microbes: 19 viruses (Influenza A (no subtype detected, H1, H1-2009, H3), Influenza B, Adenovirus, Coronaviruses (HKU1, NL63, 229E, OC43, Mers-CoV, SARS-CoV-2), Human Metapneumovirus, Human Rhinovirus/Enterovirus (not possible to distinguish Human Rhinovirus from Enterovirus due to their genetic similarity), Parainfluenza (types 1, 2, 3, 4) and RSV and four atypical bacteria: Bordetella pertussis, Bordetella parapertussis, Chlamydia pneumoniae and Mycoplasma pneumonia.

The results will be filed in the participant medical notes. The POCT^{RM} does not test for the three typical respiratory bacteria *S. pneumoniae*, *H. influenzae* and *M. catarrhalis* since these are commensally carried in the upper respiratory tract. Hence, Study Clinicians will be advised that the POCT^{RM} result should be used as a guide to clinical decision making, with final responsibility for antibiotic prescribing residing with the Study Clinician. To help with results interpretation, Study Clinicians will be provided with information describing the typical presentation of illnesses caused by the microbes tested as was done in our feasibility study.[36] Further advice will also be available via standard NHS virology services.

If the POCT^{RM} results from a participant's swab sample are reported as equivocal, the original swab sample should be retested once as per the manufacturer's instructions. If the POCT^{RM} results are reported as failed or invalid, the manufacturer's instructions should be followed. If it has not been possible to obtain POCT^{RM} results after following the manufacturer's instructions, this should be recorded in the eCRF. The participant can continue in the trial and the Study Clinician should make a decision about antibiotic prescribing based on the clinical evidence available at that time.

6.6.2 GP POCT^{RM} Test (Intervention) Group and No GP POCT^{RM} Test (Control) Group Central Research Laboratory Analysis

The remaining swab sample from participants in the GP POCT^{RM} test group and the whole swab sample from participants in the No GP POCT^{RM} test group will be stored at ambient temperature prior to transport. Samples that are not sent on the same day of collection (e.g. missed last collection time), should be stored in the fridge (2-8^{0}C) overnight and sent the next morning. Samples will be transferred within 24 hours to the central research laboratory. Samples will be stored in a linked anonymised format using a combination of trial name, Participant ID, date of birth and date of sample to permit accurate linkage to trial data and the ICF. These samples will be tested for a full array of respiratory viral and bacterial pathogens using a Taqman Low Density PCR array card assay as described in our previous study.[36] The samples will also be tested using the BioFire® RP2.1 *plus* reagent pouches run on the Biofire® Filmarray® Torch 1 system. Once analysis has taken place, any residual sample will be destroyed. All samples will be handled according to the Human Tissue Act. Further information relating to the central research laboratory analysis is available in the Laboratory Manual.

6.6.3 Study Clinician Views

Figure 1, Step 5

Immediately after randomisation and before Appointment Two, the Study Champion will ensure the Study Clinician is aware which group the participant has been allocated to and will provide the POCT^{RM} result for

those in the intervention group. The Study Clinician will then complete further data on their views (see Appendix 1, Study Clinician Views questions 1 to 7). Where the participant is in the No GP POCT^{RM} test group, this can be completed immediately after randomisation. Where the participant is in the GP POCT^{RM} test group, the Study Clinician must wait until the POCT^{RM} results are available before completing data on their views.

6.7 Appointment Two (Day 1)

Figure 1, Step 6

At Appointment Two, the Study Clinician will contact the participant to inform them which randomisation group they were allocated to, what the POCT^{RM} result was (for those in the GP POCT^{RM} test group) and to discuss treatment. Ideally this will be the same Study Clinician who was in contact with the participant at Appointment One.

6.8 Post-Appointment Two (Day 1)

Figure 1, Step 7

The Study Champion ensures the participant (or parent/carer if the participant is <16 years) completes the post-appointment participant views (see Appendix 1, Participant Views questions 1 to 5). These can be collected face-to-face or remotely (e.g. by phone or text)

6.9 Follow-Up

The Trial Diary will be completed daily by the participant until their symptoms resolve (defined as all symptoms being rated zero for two days in a row) or up to day 28, whichever comes first. The central trial team will contact participants regularly via email, text or phone to support completion of the Trial Diary. Completed paper versions of the Trial Diary should be returned to BTC. Participants will be advised that if their symptoms have not resolved by Day 28, or if they have any concerns before Day 28, they should contact their GP practice for further advice.

Participants (or parent/carer if the participant is <16 years) will be followed-up with a questionnaire at 2 months post-randomisation which will collect beliefs and intention to consult for similar future illnesses (see Appendix 1, Participant Views questions 6 and 7). Up to two reminders will be sent for cases where the questionnaires are not returned. In addition, data on any further GP consultations for RTIs will be collected from their PCMR at 2- and 6- months after randomisation. For those participants randomised in the later stages of Summer 2, there will be a shorter follow-up period to ensure that all follow-up is completed 2 months after recruitment has finished.

6.10 Optional Interviews and DCE Surveys

During the consent process, participants (or parents/carers if the participant is <16 years) will be asked whether they are interested in being contacted about taking part in an optional Participant Interview and receiving a separate optional Participant DCE Survey. Participants (or parents/carers if the participant is <16 years) can choose to be contacted about the interview and the DCE survey, just one, or neither. This does not affect their ability to take part in the main trial.

Clinicians from each site will be asked whether they are interested in being contacted about taking part in an optional Clinician Interview. In addition, Study Clinicians involved in the trial and other clinicians recruited through the West of England Clinical Research Network will be invited to complete a Clinician DCE Survey.

See section 7 for further details about the interviews and DCE surveys.

6.11 Blinding of Allocated Group

Post-randomisation, the participants and the Study Clinicians at each site will be informed of the allocated randomisation group since the subsequent processes for each group differ.

The Trial Management Group (TMG) will not review unblinded primary or secondary outcome data until all follow-up and data queries have been resolved at the end of the trial. They will have unblinded reports of those post-randomisation process measures being used to monitor trial conduct. Two statisticians based at BTC will support this trial. The trial statistician will have access to unblinded data while preparing confidential reports for the Data Monitoring Committee (DMC)and will perform all disaggregated analyses according to a pre-specified SAP in addition to attending closed DMC meetings as required.

6.12 Withdrawal from the Trial

Participants can choose to withdraw for any reason at any time during their involvement in the trial, they will be able to withdraw from the entire study or just individual elements (e.g. interviews, completion of questionnaires, PCMR review). Participants will be asked about their reason for withdrawing but do not have to give one. The Chief or Principal Investigators can also decide to withdraw participants based on clinical opinion at any time during the trial. In the event of any form of withdrawal, data obtained up to this point will be retained for analysis.

Following withdrawal from the trial, patient care will be decided by their GP, according to usual practice.

6.13 Participant Payments and Communication

In recompense for their time and as a thank you, participants (or parent/carer if the participant is <16 years) will be offered a £20 voucher on completion of the 2 Month Questionnaire. A further £10 voucher will be offered to participants (or parent/carer if the participant is <16 years) on completion of the optional DCE survey and an additional £10 voucher will be offered to participants (or parent/carer if the participant is <16 years) on completion of the optional qualitative interview.

If participants (or parent/carer if the participant is <16 years) wish they will be sent a newsletter with updates about the trial progress and a summary of the results at the end of the trial.

6.14 End of Trial

Participants end their involvement with the trial when their 2 Month Questionnaire is completed (or efforts to obtain it have been unsuccessful), or they have withdrawn from the trial.

The end of the trial will be when the last participant has completed their 2 Month Questionnaire (or efforts to obtain it have been unsuccessful), all data has been extracted from the participants' PCMRs, all data queries have been resolved and the database has been locked, with subsequent data analysis completed.

6.15 Trial Stopping Rules

The trial may be prematurely discontinued by the Sponsor, CI, Regulatory Authority or Funder based on new safety information or for other reasons given by the DMC, the Trial Steering Committee (TSC), regulatory authority or ethics committee concerned.

The trial may also be prematurely discontinued due to lack of recruitment or upon advice from the TSC, who will advise on whether to continue or discontinue the trial and make a recommendation to the Funder. If the trial is prematurely discontinued, no new participants will be recruited, and a decision on data collection on active participants will be made in discussion with the TSC, DMC and Sponsor.

7. MIXED-METHODS EVALUATION OF MICROBIAL, BEHAVIOURAL AND ANTIBIOTIC MECHANISMS

Our mixed-methods feasibility study[36] showed the BioFire® FilmArray® Torch 1 system was used in around one third of eligible participants, and was acceptable and liked by the clinicians. These perceptions were consistent and complementary to those reported in the literature: that clinicians often feel pressure to

prescribe;[55] that a key advantage of POCTs is to help manage patient expectations for antibiotics;[56] and that disadvantages included uncertainties about test performance, interpreting results, detraction from clinical reasoning, costs, time and patients not wanting or demanding the tests.[57, 58]

Since our feasibility study was not resourced to investigate the participant perspective, we sought evidence from published evidence. Compared to studies of professional perceptions, there was a paucity of participant evidence.[59] Of the three studies we found, one showed participants would accept a POCT as part of routine care.[58] Another showed that participants perceived a POCT could support clinician prescribing decisions by helping them understand when an antibiotic was, and was not, needed.[60] Participants had largely positive views about the use of the POCT, with some reporting they would wait longer before consulting for a similar illness in the future.[59] Finally, POCTs have been shown to increase participant satisfaction, though only in the context of chronic disease management.[60]

Using social cognitive theory,[61] these findings were used to inform the behavioural mechanistic hypotheses and the associated logic model (Figure 2) which is a visual representation of how the POCT^{RM} might influence clinician and patient expectations (beliefs) and self-efficacy (confidence) to reduce antibiotic prescribing. The model consists of three major components: (i) motivation for intervention use which lists the psychological reasons why patients and clinicians may want to use the POCT^{RM}; (ii) the proposed mediating variables i.e. the hypothesised mechanisms by which the intervention will work to affect outcomes; and (iii) the short- and longer-term outcomes.

Figure 2: Logic model showing hypothesised causal relationships and mediators

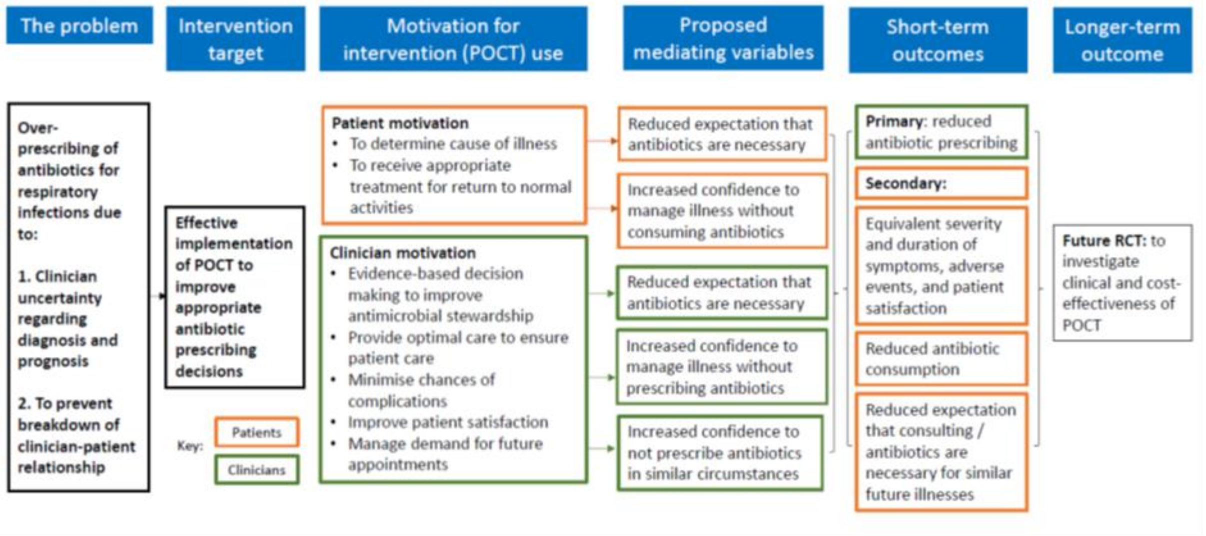

Social cognitive theory proposes that the environment plays a key role in influencing an individual's behaviour,[62] and that an individual's belief in their ability to exercise control over their environment is one of the most important mechanisms involved in successful behaviour change.[62] If an individual perceives their environment to be controllable and supportive, they will be more likely to succeed in performing the desired behaviour.[62] Therefore, we hypothesise that POCT^{RM} use will lead to changes in beliefs that will drive clinician behaviour change (reduced prescribing, and more so when a virus is detected) by:

1. Reducing clinician and patient beliefs that prescribing is necessary to avoid negative outcomes – for clinicians: complications, re-consultations, patient dissatisfaction – and for patients: increased severity and/or longer duration of illness; and

2. Increasing clinician and patient confidence to manage the illness without antibiotics.

In the context of the current illness, changing these beliefs would reduce prescribing for that illness. If these beliefs are also generalised to future similar illnesses, they could reduce future clinician prescribing and patient consulting,[63] but importantly, consulting could increase if patients believe testing is necessary to determine if treatment is needed.

We will also explore the effect of the POCT^{RM} use on participant belief that an antibiotic is necessary on antibiotic consumption. We will update and refine the logic model after completing participant and clinician interviews, in readiness for a future pragmatic cost-effectiveness trial.

7.1 Qualitative Interviews

A subset of participants (or parent/carer if the participant is <16 years) from both the intervention and control groups will be invited to take part in an optional interview. These interviews will be held in person, via telephone, or via videoconferencing >7 days after taking part in the main trial using a platform approved for this purpose by University of Bristol, Information Governance. The interviews will explore any changes in beliefs and intended behaviour resulting from POCT^{RM} use and the factors that influence whether participants (or parent/carer if the participant is <16 years) decide to visit their doctor with a respiratory infection. Similarly, a subset of clinicians will also be interviewed to explore their perceptions of the use of the POCT^{RM} and how it impacts on antibiotic prescribing.

Semi-structured interviews will be conducted with up to 30 participants (or parent/carer if the participant is <16 years) and 20 clinicians. The schedule of semi-structured questions will be used to guide data collection. The interview schedules will continue to evolve so that relevant topics important to participants are discussed and to ensure necessary data is collected. Participants will initially be purposefully selected for variation in age, sex, whether they are a patient or parent/carer, number of courses of antibiotic treatment received in past 12 months for respiratory tract infections, POCT^{RM} result, antibiotic prescribing decision, and from practices with lower and higher deprivation areas. Clinicians will be purposefully sampled to cover variation in age, gender, years in practice and practice characteristics. In both groups, sampling will become more theoretical as analysis progresses.

Participant (or parent/carer if the participant is <16 years) interviews will explore reasons for visiting the GP on this occasion, their general context in terms of visiting the GP, their experience of the POCT^{RM}, their perception of the value of the test results, their views about the eventual prescribing decision and their perceptions about what action they might take in future similar situations. Clinician interviews will explore their perceptions of the positive and negative impacts of POCT^{RMs} to increase antibiotic prescribing appropriateness, the clinical situations in which they most and least valued the new microbial knowledge, how it influenced their clinical reasoning and participant management, and how various factors e.g. cost, waiting time for results, participant characteristics, practice characteristics etc., would influence their use of the POCT^{RM} in routine clinical practice.

The interviews will be audio-recorded. All data will be fully transcribed and analysed using Framework analysis to generate more detailed understanding of behavioural mechanisms[64] and for the purpose of generating conceptual attributes and wording for the DCE survey.[65, 66] Open coding of early transcripts will be followed by the development of a hierarchical coding schedule which will be applied to all data. Analytic accounts will be generated for batches of interviews, facilitating comparison across participants (or parent/carer if the participant is <16 years) and clinicians, and the generation of mutually exclusive attributes relating to the two issues that will be pursued in the DCE surveys.

7.1.1 Consent Process for Qualitative Interviews

If selected for an interview, the qualitative researcher will contact the participant (or parent/carer if the participant is <16 years) or clinician to explain more about the interview, answer any questions and, if they agree, arrange a convenient time to conduct the interview. Participants (or parent/carer if the participant is <16 years) and clinicians will be given an information sheet about the qualitative interviews. Informed consent will be taken using an e-consent mechanism or if this is not possible paper-based consent will be considered.. Compensation for participants (or parent/carer if the participant is <16 years) for their time in completing the interviews is stated in section 6.13. Clinicians will also be offered a £40 voucher as compensation for their time in completing interviews.

7.1.2 Data Protection and Participant Confidentiality in Relation to the Qualitative Data

All audio-recordings from interviews will be made using an encrypted audio-recorder. Interview data captured on the encrypted audio-recorder will be transferred to a University of Bristol computer as soon as

possible after each interview. If interviews are conducted through a video conferencing platform, then only the audio-recording file will be transferred securely to the University of Bristol and both the audio and video files will be deleted from the video-conferencing platform. All data will be stored on password protected computers maintained by the University of Bristol.

Audio-recordings will be transcribed by University of Bristol employees or University approved transcription services. Transcripts will be labelled with a trial-assigned participant number, edited to ensure anonymity of respondents and stored securely adhering to the University's data storage policies. Audio-recordings and transcripts will be retained by the University of Bristol where anonymised quotations and parts of voice modified recordings may be used by the University for training, teaching, research and publication purposes for this and future studies. Anonymised transcripts may be made available to other researchers (including those outside of the University of Bristol) by controlled access if they secure the necessary approvals for purposes not related to this trial, subject to optional individual written informed consent from participants.

7.2 Discrete Choice Experiments

DCEs provide a method that allows for the quantification of individual preferences and stated behaviours of patients and other stakeholders in the provision and outcome of healthcare services. DCEs involve presenting participants with a range of hypothetical scenarios, consisting of characteristics of the good or service that are considered important to a choice an individual would make in practice.[67, 68] The responses provided by participants allow for the estimation of the relative importance they attach to different characteristics associated with a good or service based on the choices they make in the hypothetical scenarios. DCEs are increasingly used in healthcare to help develop a better understanding of patient and healthcare professionals' preferences for treatment and screening options for patients.[69]

Although related DCEs exist in the role of antibiotic prescribing from the clinician[70, 71] and general population[72, 73] perspectives, we are unaware of any previous attempt to directly capture the impact of the POCT^{RM} in the decision-making for patients or clinicians.

This trial will involve DCEs for two different groups. The first group is that of patients (or parent/carer if the participant is <16 years). This work will explore the role of the POCT^{RM} in the help seeking behaviour of patients, and parents/carers for those aged under 16 years old. The second group is that of clinicians. Here, the work will explore the perceived role of the POCT^{RM} in clinicians' provision of care for patients. Although the role of the POCT^{RM} will be important for both groups, the design of each DCE will capture the particular characteristics at play for patients and clinicians with the proposed introduction of the POCT^{RM} to usual care. Each DCE will provide useful information to decision-makers in understanding how the POCT^{RM} may play a role in the health seeking behaviour of patients, and how clinicians would prefer to use the POCT^{RM} in their usual care.

The identification of relevant attributes for each group will be primarily drawn from the qualitative interviews from the participant (or parent/carer if the participant is <16 years) and clinician groups respectively (see section 7.1) as recommended.[69, 74] At least two DCEs will be developed covering the separate participant and clinician groups. For the participant group, however, it is possible that the qualitative work will show that values (and therefore attributes) differ when participants are thinking about themselves as patients, compared with when they are thinking about their children as patients.

7.2.1 Consent Process for the DCEs

The DCE will be included as an optional element for participants in the trial. A summary of the DCE will be provided in the main PIS, with an optional item on the main consent form for the participant (or parent/carer if the participant is <16 years) to indicate whether they agree to receive the DCE survey. After completion of their Trial Diary, participants (or parent/carer if the participant is <16 years) who have opted in to the DCE survey will be sent the survey via email. More information about the DCE will be provided in this email. Reminder emails will be sent to participants (or parent/carer if the participant is <16 years) who have not completed the DCE survey after a week and a month of the original email being sent.

The sample for the clinician DCE will be drawn from a much broader basis than those involved in the conduct of the trial. Clinicians will be recruited through the Clinical Research Network West of England. Informed consent will be taken using an e-consent mechanism or is this is not possible, verbal consent will be considered, see section 6.4.1.

Compensation for participants (or parent/carer if the participant is <16 years) for their time in completing the DCE surveys is stated in section 6.13. Clinicians will also be offered a £40 voucher as compensation for their time in completing the DCE surveys.

7.2.2 DCE Data Analysis

As the DCEs will be developed from the findings in the qualitative interviews with participants (or parent/carer if the participant is <16 years) and clinicians (see section 7.1), we are unable to calculate a sample size for each DCE survey at this time. DCE sample size requirements are based on the number of attributes and levels required, the number of scenarios posed to each participant, as well as the statistical significance and power levels, the statistical model used in the DCE analysis and the initial belief about the parameter values.[75] Piloting of DCEs (with approximately n=10-20 participants, or parents/carers if the participant is <16 years, for each DCE survey) and use of efficient survey designs, such as D-efficiency, will help to ensure appropriate sample sizes. We anticipate a sufficient sample size will be reached through the recruitment of participants (or parent/carer if the participant is <16 years) from the trial, as well as through the Clinical Research Network West of England for clinicians.

Best practice in the design[76] and analysis[77] of DCEs will be followed. The design of the DCEs will be generated using Ngene software (www.choice-metrics.com). Data collection for both DCEs will be conducted using REDCap online surveys and databases (https://www.project-redcap.org/). Statistical analysis will consist of a multinomial logit model if there is no heterogeneity across participants, in which case, a mixed logit model will instead be applied. Statistical analysis will be undertaken in Latent Gold 6.0 statistical software (https://www.statisticalinnovations.com/latent-gold-6-0/).

8. DIAGNOSTIC EQUIPMENT

8.1 General Information

The diagnostic equipment being used in this trial are the BioFire® RP2.1 *plus* reagent pouches which will be run on the Biofire® FilmArray® Torch 1 system.[26] The BioFire® RP2.1 *plus* reagent pouches are intended for use with the BioFire® FilmArray® Torch system for the simultaneous qualitative detection and identification of multiple respiratory viral and bacterial nucleic acids in nasopharyngeal swabs obtained from individuals suspected of respiratory tract infections, including COVID-19.

The FilmArray Torch is an automated *in vitro* diagnostic (IVD) device intended for use with approved IVD FilmArray panels. The FilmArray Torch is intended for use in combination with assay specific reagent pouches to detect multiple nucleic acid targets contained in clinical specimens. The FilmArray Torch interacts with the reagent pouch to both purify nucleic acids and amplify targeted nucleic acid sequences using nested multiplex PCR in a closed system. The resulting PCR products are evaluated using DNA melting analysis. The FilmArray Torch software automatically determines the results and provides a test report.

8.2 Distribution and storage of Biofire® FilmArray® Torch 1 Machine and Reagent Pouches

All sites will receive a Biofire® FilmArray® Torch 1 machine along with BioFire® RP2.1 *plus* reagent pouches. The Central Trial team will install and maintain the Biofire® FilmArray® Torch 1 machine at each site, with support from BioMérieux. Sites will receive all necessary training on how to use the Torch 1 machine.

A third party logistics company will manage the distribution of the Torch 1 machines from BioMérieux to each site, the transfer of Torch 1 machines from site to site at the end of Wave 1, and the transfer of Torch 1 machines from sites back to the University of Bristol or other specified location at the end of the trial. BTC will manage the distribution of BioFire® RP2.1 *plus* reagent pouches to each site and to the central laboratory. At the end of the trial, any unused BioFire® RP2.1 *plus* reagent pouches must be returned to BTC.

The Biofire® FilmArray® Torch 1 machine needs to be installed in a clean, well-ventilated area that allows at least 2.6cm between the rear panels and any other surfaces (such as the wall) to allow for proper air flow. The depth of the bench-top space should be at least 77cm, the width of the bench-top space should be at least 49cm and the height of the space should be at least 30cm. The Biofire® FilmArray® Torch 1 machine must not be used in close proximity to sources of strong electromagnetic radiation (unshielded intentional radio frequency sources, for example) because these may interfere with the operation of the FilmArray Torch.

BioFire® RP2.1 *plus* reagent pouches are to be stored securely at room temperature (15 to 25°C) and must NOT be refrigerated. The pouches must not be stored near heating or cooling vents or in direct sunlight. Pouches should not be removed from their packaging until a sample is ready to be tested and once the pouch packaging has been opened, the pouch should be used as soon as possible (within approximately 30 minutes).

9. SAFETY

9.1 Operational Definitions

The following definitions and classifications will apply to all safety reporting in this trial.

Table 7: Definitions of Adverse Events

Term	Definition
**Adverse Event (AE)**	Any unfavourable and unintended sign or symptom that develops or worsens during trial participation, whether or not it is considered to be related to the trial intervention.<br><br>The following are not classed as AEs: continuous and persistent disease or symptoms, present before the trial, which fail to progress; signs or symptoms of the disease being studied (in this case respiratory infection); or treatment failure.
**Serious Adverse Event (SAE)**	A serious adverse event is any untoward occurrence that:<br>• results in death<br>• is life-threatening[A]<br>• requires inpatient hospitalisation or prolongation of existing hospitalisation[B]<br>• results in persistent or significant disability/incapacity<br>• consists of a congenital anomaly or birth defect<br><br>Other 'important events' may also be considered serious if they jeopardise the participant or require an intervention to prevent one of the above consequences.<br><br>[A]*The term "life-threatening" in the definition of "serious" refers to an event in which the participant was at risk of death at the time of the event; it does not refer to an event which hypothetically might have caused death if it were more severe.*<br><br>[B]*The definition of hospitalisation is an unplanned overnight stay. Note, however, that the patient must be formally admitted – waiting in outpatients or an Accident & Emergency Department (A&E) would not count as hospitalisation (even though this can sometimes be overnight). Prolongation of an existing hospitalisation qualifies as a SAE. Planned hospital stays would not be counted as SAEs, nor would stays in hospital for "social reasons" e.g. respite care or the fact that there is no-one at home to care for the patient. Also, if patients had a day-case operation, this would not qualify as hospitalisation. However, if a planned operation was brought forward because of worsening symptoms, this would be considered as a SAE. Hospitalisations for the purpose of the intervention are an exception to SAE reporting unless complications occur.*

Table 8: Classification of Severity

**Mild event**	An event that is easily tolerated by the participant, causing minimal discomfort and not interfering with everyday activities.
**Moderate event**	An event that is sufficiently discomforting to interfere with normal everyday activities.
**Severe event**	An event that prevents normal everyday activities.

Table 9: Classification of Relatedness

**Not related**	Temporal relationship of the onset of the event, relative to administration of the intervention, is not reasonable or another cause can by itself explain the occurrence of the event.
**Unlikely to be related**	Temporal relationship of the onset of the event, relative to administration of the intervention, is unlikely and it is likely there is another cause which can by itself explain the occurrence of the event.
**Possibly related**	Temporal relationship of the onset of the event, relative to administration of the intervention, is reasonable but the event could have been due to another, equally likely cause.
**Probably related**	Temporal relationship of the onset of the event, relative to administration of the intervention, is reasonable and the event is more likely explained by the intervention than any other cause.
**Definitely related**	Temporal relationship of the onset of the event, relative to administration of the intervention, is reasonable and there is no other cause to explain the event, or a re-challenge (if feasible) is positive.

9.2 Identification of AEs

AEs are expected to occur throughout the course of the trial. Site teams are responsible for recording appropriate AEs for their participants during the trial. The central trial team will contact the sites if additional information is required e.g. to ascertain the nature and severity of an AE. If a participant attends a routine i.e. non-trial related appointment, and an AE is reported, the site team will assess and log this according to the trial recording and reporting procedures as detailed below.

9.3 Classification of (S)AEs

The PI at each site is responsible for assessing all AEs and categorising whether they are serious, expected and related to the trial procedures.

The only event expected due to the trial procedures is temporary discomfort and/or slight nose bleed from swabbing.

As is proportionate to the nature of the trial, only Suspected Unexpected Serious Adverse Reactions (SUSARs) will require expedited reporting to the Sponsor (see section 9.5.1). All other SAEs will be recorded on the SAE Summary Log (see section 9.5).

9.4 Recording and Reporting Non-Serious AEs Related to the Intervention or Trial Procedures

A non-serious AE is an adverse event which does not satisfy the above definition of an SAE.

Only non-serious AEs that are assessed as being **possibly, probably or definitely related to the intervention or trial procedures** will be recorded in the relevant trial documentation e.g. CRF. They should also be recorded in the participant's medical notes by the site team. The participant should be followed up by the site team until the event resolves. The recording framework for non-serious AEs is shown in Figure 3.

Figure 3: Recording Framework for AEs Assessed as Non-Serious

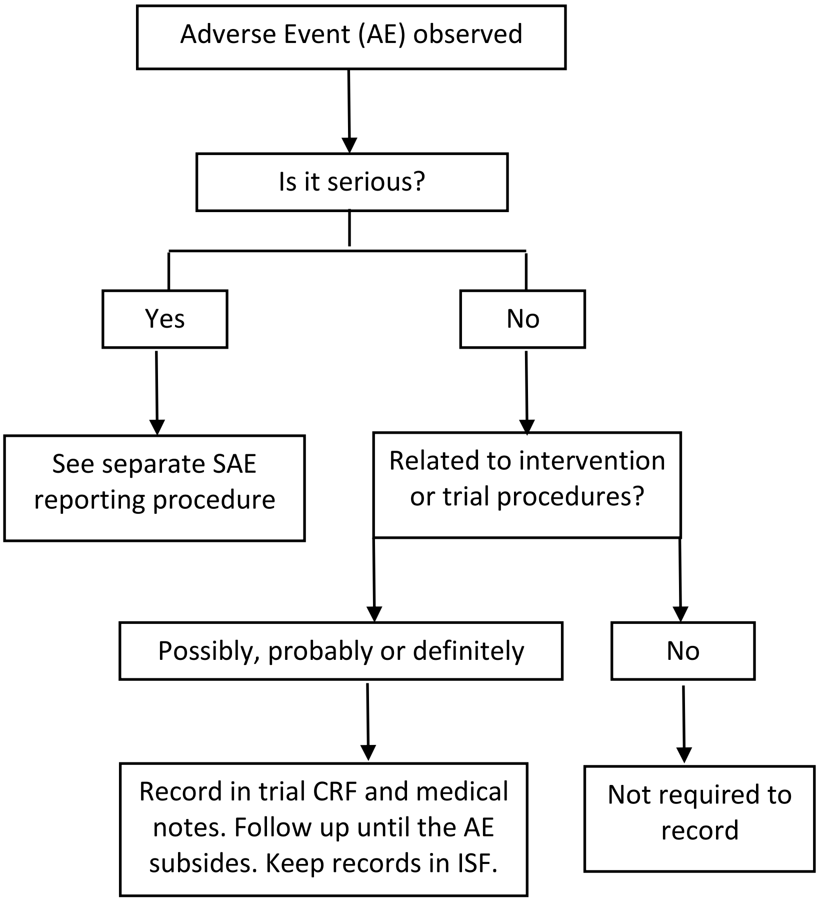

If the event is a SAE, the site team should follow reporting procedures for SAEs outlined in section 9.5.

Non-serious AEs that are unrelated to the intervention do not need to be recorded. A record of all recordable AEs (including SAEs) must be kept in the ISF.

The central trial team will prepare regular summary reports of all recorded non-serious AEs for discussion at relevant oversight meetings, including with the Sponsor.

9.5 Recording SAEs

Sites will record all SAEs in the SAE Summary Log in the eCRF. Data capture will be via the trial database, or in a suitable electronic format or via paper records with subsequent data entry, where necessary. The central trial team will review the SAE Summary Log regularly for monitoring and reporting purposes and will prepare regular summary reports of all SAEs for discussion at relevant oversight meetings, including with the DMC.

SAEs should also be recorded in the participant's medical notes by the site. The participant should be followed up by the site until the event resolves or a final outcome has been reached. The PI, or delegate, should complete the overall assessment. Information not available at the time must be forwarded once available.

As is proportionate to the nature of the trial, only SUSARs will require expedited reporting to the Sponsor.

9.5.1 Reporting of SUSARs

Any SAEs which are assessed by the PI as being related to trial procedures and unexpected for that procedure (not listed under section 9.3) are classed as SUSARs and must be documented on the full SAE Report Form, which is provided by the central trial team. An initial report may be provided orally but a written SAE Report Form must be completed and submitted within 24 hours of staff becoming aware of the

event. Each SAE must be reported on a separate SAE Report Form. The reporting framework for SAEs is shown in Figure 4.

Any change of condition or other follow up information relating to a previously reported SUSAR will be reported on a separate SAE Follow-Up Report Form.

Sites should scan and email the SAE Report Form, with high importance, to UHBW on behalf of the Sponsor (in accordance with their SAE reporting SOP), the RAPID-TEST central trial team and copy Prof Alastair Hay (CI), see contact details on pages 2-3.

The central trial team will confirm email receipt and if required, the Sponsor will forward the completed form to REC within the necessary reporting periods.

NB: *typical working hours of the central trial team (UK): Monday to Friday, 09:00-17:00 (subject to variation). In the event of University closure dates or limited availability, an out of office automatic response will notify recipients of alternative contact details/arrangements.*

All SAEs will be further reported to the DMC as part of their oversight meetings. The central trial team will be responsible for all other reports to relevant regulatory authorities and trial oversight committees.

Figure 4: Recording Framework for SAEs

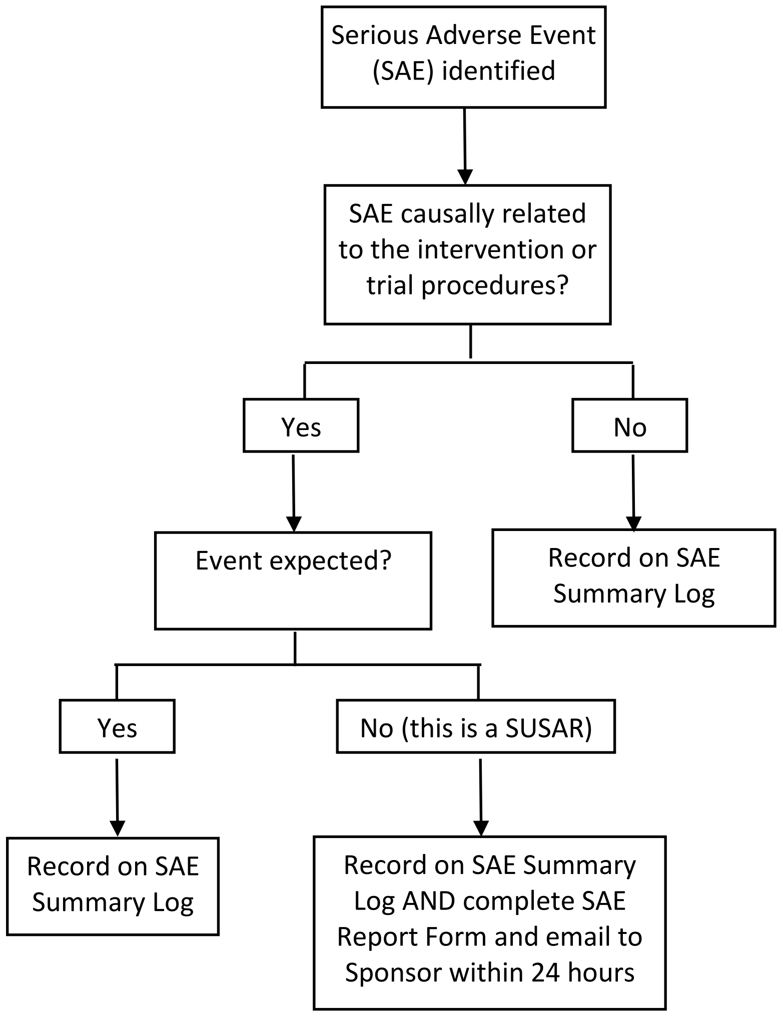

10. STATISTICS AND HEALTH ECONOMICS ANALYSIS

10.1 Sample Size Calculation

10.1.1 Clinical Sample Size

Antibiotic prescribing at the index consultation is the primary outcome measure. The largest individual patient data meta-analysis to date reported the use of antibiotics in 51,780 patients in observational and 55,682 patients in experimental studies, across a wide spectrum of respiratory infections and patient groups, similar to those we propose to recruit in RAPID-TEST. In both observational and experimental studies, immediate and delayed antibiotics were prescribed to 47% and 18% respectively (total 65%).[3]

We anticipate factors both increasing and decreasing antibiotic prescribing in the control group of the RAPID-TEST trial. Patients in whom clinicians have the most uncertainty regarding antibiotics will be selected (suggesting an average 50% prescribing rate) but it is known that antibiotics are often prescribed 'just in case'.[17]

Previous stewardship trials of POCTs have used varying minimum clinically important difference (MCID) definitions of absolute prescribing reductions, from 10%[21] to 20%,[22] with actual reductions observed of 15%[21] and 22%.[22] Since POCT^{RMs} are expensive, we selected a MCID of 15%.

Assuming an antibiotic prescribing rate of 60% in the control group, 244 participants per group will allow a true reduction to 45% in the GP POCT^{RM} test group to be detected with 90% power at 5% significance. A total randomisation target of 514 will allow for 5% attrition. This number will provide at least 90% power to detect the same absolute difference if the antibiotic prescribing rate is found to be higher or lower than 60% in the control group.

If the POCT^{RM} results in fewer antibiotic prescriptions, we wish to demonstrate non-inferiority of the POCT^{RM} in terms of not increasing mean symptom severity at days 2 to 4 to a clinically significant extent. Assuming 80% completion of Trial Diaries (as previously achieved in adults[78] and children[79]) we will have data for symptom severity at 2 to 4 days in 206 participants per group. Data on 7,000 adults and children managed without POCT indicates a mean symptom severity at days 2 to 4 of 2.3 (standard deviation 1.5).[3] We know this measure's distribution is positively skewed and have used a calculation that accommodates this (assuming equal skew in both groups, quantified as a coefficient of variation of 0.7).[80] Assuming, in truth, no difference between groups, 206 participants in each group will give 90% power for a one-sided 95% confidence interval to exclude increases in the average symptom score of 20% or more.

10.1.2 Mechanistic Sample Size

Next, we illustrate the power of a study with 244 in each group for investigating the hypothesis that a virus detected/not detected result is on the causal pathway between the POCT^{RM}, the clinician's belief that an antibiotic is necessary and prescribing behaviour. The power calculation is based on an interaction term (capturing any difference in the effect of the test result on prescribing between the randomly allocated groups) in the logistic regression model:

Log odds(prescription) = [Group] + [Virus detected vs. not detected] + [Interaction]

The null hypothesis for the interaction term is that the 'virus detected/not detected' result has the same effect on prescribing in the intervention and control groups. The statistical power to test this hypothesis was evaluated in a simulation study outlined in Table 10. While the test result will not be made available to inform the clinical decision in the control group, the simulations allow for GPs being better than chance at prescribing antibiotics in cases who will benefit (microbial testing in the control group will allow us to observe if this is the case). Similarly, the interaction term in the analysis model separates the effect of the POCT^{RM} from GPs' ability to appropriately prescribe.

Two scenarios are considered for the POCT group (Table 10). In Scenario One (Figure 5, red line) antibiotic prescribing is affected both when a virus is detected (prescribing reduced) and when a virus is not detected (prescribing increased to P1=90%). Note that this scenario is based on the prescribing changes observed in our feasibility study,[36] and gives the absolute difference in prescribing between groups used in our sample

size calculation (60% versus 45%). In Scenario Two (Figure 5, blue line) antibiotic prescribing is only affected when a virus is detected and not when no virus is detected (P1=75% as for the control group). Hence, the scenarios differ in the assumed probability of prescription P1.

Table 10: Assumed Population (true) Antibiotic Prescribing Rates in Two Scenarios Forming the Basis of Simulation Studies to Determine the Statistical Power to Identify the Test Result is on the Causal Pathway

	Percentage prescribed an antibiotic	
	**Intervention (n=244)** *Test result informs prescribing decision*	**Control (n=244)** *Test result not available for decision*
**Scenario One: 'virus detected' and 'virus not detected' affects prescribing**		
No virus detected (40%)[a]	P1 = 90%	75%
Virus detected (60%)[a]	P2 (e.g. 15%)	50%
Overall probability antibiotic prescribed	*45%*	*60%*
**Scenario Two: only 'virus detected' affects prescribing**		
No virus detected (40%)[a]	P1 = 75%	75%
Virus detected (60%)[a]	P2 (e.g. 15%)	50%
Overall probability antibiotic prescribed	*39%*	*60%*

[a] Proportions informed by feasibility study[36]

The population probability of a prescription in those for whom the POCT identified a virus (Table 10, P2) was varied within each scenario. Assuming a population probability of prescription for 50% of participants in the control group for whom a virus would be identified, the x-axis of Figure 5 presents prescribing as a relative risk P2/50%. Following the example in Table 10, P2 = 15%, the true effect of detecting a virus is a relative risk of 0.3 i.e. a 70% reduction in prescribing in this subgroup.

For each of the two scenarios, 1000 simulated datasets were generated for values of P2 from 0.20 to 1.00. For each simulation, the "observed" probability of detecting a virus, and each of the four prescription probabilities, were generated according to a binomial distribution. The above logistic regression model was fitted to each dataset, and the p-value for the interaction term calculated using a likelihood ratio test. The power (Figure 5, y-axis) for each combination of scenario and P2 was calculated as the percentage of $p<0.05$.

Figure 5 shows that, for both scenarios, 244 participants per group provides >90% power to detect an effect of the test result of at least a 60% relative reduction in prescribing among the test positives. Hence the proposed trial is large enough to identify dichotomised mechanisms on the causal pathway, with greater power expected for mechanisms measured on a scale.

Figure 5: Statistical Power Curves for Scenario One (red curve) and Scenario Two (blue curve)

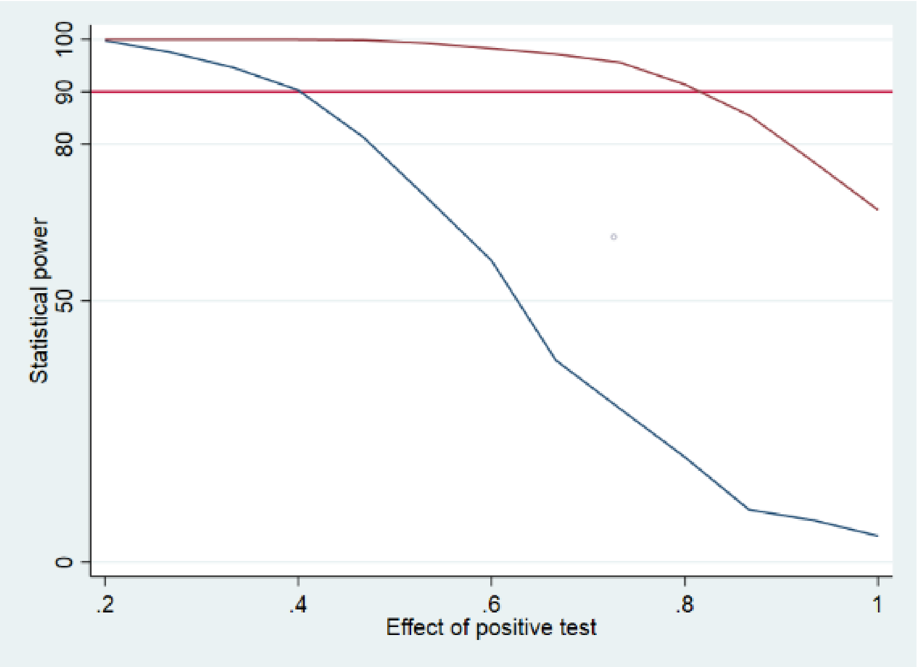

The x-axis is the relative reduction in prescribing in the test positives, comparing the POCT group (test result informs decision) and comparison group (test result does not inform decision)

10.2 Statistical Analysis

A detailed statistical analysis plan will be finalised and made publicly available ahead of the completion of recruitment. The primary analysis will be of observed data and conducted according to the intention to treat principle. A logistic regression equation will estimate the causal association between the primary outcome of antibiotic prescribing and allocated intervention group as an odds ratio, presented with 95% confidence interval and p-value. Further covariates will include participant age and chronic lung disease (used to stratify randomisation). Variations between participating GPs in prescribing tendency will be accommodated e.g. dummy variables to distinguish each GP. Sensitivity analyses will gauge the robustness of the conclusions to different assumptions about missing data. The above approach will be adapted e.g. through the choice of a suitable regression model, to the secondary outcome variables such as symptom severity at 2 to 4 days.

As described for the statistical power analysis, whether the result of the POCT^{RM} influences prescribing of an antibiotic will be investigated in a logistic regression model with covariates including allocated group (results inform the clinical decision or not), result (virus detected or not), and the interaction between the two. The interaction term will capture any evidence that the test result is influencing the prescribing decision, rather than a non-specific effect of the POCT^{RM} which is independent of the result, and distinguish this effect from any underlying ability of GPs to prescribe to those participants who will benefit from an antibiotic. Further investigations of the causal pathway will include the logistic regression model:

Log odds(confident an antibiotic will benefit) = Group + Result + Interaction

Where the outcome measure is GP belief that an antibiotic is necessary (assessed immediately after the POCT^{RM} result is made available), the interaction term will capture the evidence of impact of the test result on GP belief, allowing for the different impacts of a virus being detected and no virus detected. If there is evidence the POCT^{RM} result impacts GP beliefs and prescribing, the association between beliefs and prescribing will be examined for consistency with GP belief mediating between the test result i.e. how do GPs interpret and use the test information, and the prescription of an antibiotic. We anticipate a greater probability of an antibiotic prescription when the GP has greater confidence an antibiotic is necessary, in which case we may infer that the POCT^{RM} reduces antibiotic prescribing by identifying a potential viral

cause in the majority of cases leading to high confidence that an antibiotic will be of benefit for fewer participants than in the comparison group.

10.3 Additional Health Economics Analysis

As this is an efficacy evaluation, no cost-effectiveness analysis will be conducted in this trial. However, we consider a future effectiveness trial would need to be able to demonstrate cost-effectiveness with regards to: (i) symptomatic improvement and/or (ii) reduced antibiotic use. There are likely to be two key perspectives in assessing cost-effectiveness.

- The policy maker viewpoint, which is likely to be interested in the overall impact on health (current and future), and would involve current health gain from diagnosis/treatment and possible detriments to future health through generation of resistance.[81] This could be a future piece of work.
- The patient perspective, which is mainly about impact on current health. This has been difficult, but not impossible[82] to capture using the generic tools usually used in cost-effectiveness analysis because the impacts for most part are so short-lived. In a previous study, we showed changes in EQ-5D (associated with acute lower respiratory tract infections in adults) could be detected when measured weekly.[82] The EQ-5D-5L will be used for participants ≥16 years and the proxy EQ-5D-Y will be used for children ≥4 years and <16 years alongside the Trial Diary that will also be completed by the parent or carer.

The main health economics contribution in this trial is the qualitative interviews and application of two DCE surveys to better understand participant and clinician preferences with regards the use of POCT^{RM} tests in usual care (see Section 7 for further details).

11. DATA MANAGEMENT

11.1 Source Data and Documents

When a participant is screened they will have a unique participant identification number allocated. Personal data will only be collected after consent. It will be entered directly onto the password protected database and maintained on a SQL Server database system within the University of Bristol which is only accessible to members of the research team. Any data stored on laptops will be encrypted. Any information that is analysed or transferred outside the EEA will be anonymised. Participants will be asked to consent to their name, date of birth, and contact details being stored on the secure database with the central research team.

Data obtained by paper will also be entered onto the password protected database. Information capable of identifying individuals will be held in the database with passwords restricted to trial staff. Information capable of identifying participants will not be removed from University of Bristol or clinical centres or made available in any form to those outside the trial, for the exception of NHS digital for linkage.

Consent forms and clinical letters with personal identifiable data will be stored separately in a locked filing cabinet. Participant details will be anonymised in any publications that result from the trial.

Source data for this trial will consist of certified scanned copies and/or paper copies of the consent form, participant completed questionnaires as well as the electronic case report forms designed specifically for the trial.

11.2 Case Report Forms (CRFs)

Case report forms at trial sites will be completed using the secure trial database. Questionnaires from participants will be identifiable only by participant identification number and will be returned by the participant by post or via electronic means to the central research team. Any paper copies will be stored in a secure locked cabinet in an access-controlled area.

11.3 Data Handling and Record Keeping

Data will be collected and retained in accordance with the UK Data Protection Act 2018 and UK General Data Protection Regulation 2018 (GDPR).

For this trial, research data will be kept for at least 5 years. For children under the age of 16 at recruitment, research data will be kept until their 25th birthday. Personal data e.g. name and address, or any data from which a participant might be identified, will not be kept for longer than is required for the purpose for which it has been acquired. Documents will be reviewed by the CI before being destroyed.

11.4 Access to Data

For monitoring purposes, the CI will allow monitors from the Sponsor (or delegate), persons responsible for the audit, representatives of the REC and other Regulatory Authorities to have direct access to source data/documents.

The Trial, and Data, Manager (in collaboration with the CI) will manage access rights to the data set. Prospective new users must demonstrate compliance with legal, data protection and ethical guidelines before any data are released.

11.5 Archiving

This trial will be sponsored by the University of Bristol who are also the data custodian. All research data will be retained in a secure location during the conduct of the trial and for at least 5 years after the end of the trial, when all paper records will be destroyed by confidential means. For children under the age of 16 at recruitment, research data will be kept until their 25th birthday. An archiving plan will be developed for all trial materials in accordance with BTC SOPs.

11.6 Access to the Final Trial Data Set

Members of the TMG will develop a data sharing policy that is consistent with the University of Bristol policy. It is anticipated that anonymised trial data will be kept for future analysis and may be shared with other researchers, including those outside of the UK, EU and EEA, to enable international prospective meta-analyses.

The final trial data set will be stored as restricted data on the University of Bristol's research data repository. Data will be made available to approved bona fide researchers only after their host institution has signed a data access agreement which will be confirmed by the CI or appointed nominee. Details of how to request access are available at the University of Bristol's data repository website.

12. TRIAL MANAGEMENT

The CI will take overall responsibility for managing the various components of the trial, with the support of the Trial Manager(s) and will meet regularly (as required) with the leads for each component. The BTC will support the delivery and conduct of the trial.

A TSC and a DMC will be established in conjunction with the TMG to provide oversight of the trial on behalf of the funder.

12.1 Trial Management Group (TMG)

The TMG will have responsibility for the day-to-day management of the trial and will report to the TSC. The TMG will comprise of the CI, all Co-Investigators, Trial Manager and other key BTC trial staff. The TMG will meet on a regular basis with a core working group of staff having frequent progress meetings.

12.2 Trial Steering Committee (TSC)

Membership, responsibilities, and reporting mechanisms of the TSC will be formalised in a TSC charter. The TSC will make recommendations and key decisions during the trial to the TMG and minutes of TSC meetings will be sent to the funder. The TSC will comprise an independent Chairperson plus at least three others

covering expertise in statistics, primary care and trials at a minimum. Observers may also attend (including other members of the TMG or members of other professional bodies) at the invitation of the Chair. The TSC will meet for the first time prior to recruitment of the first participant and then at agreed intervals thereafter.

12.3 Data Monitoring Committee (DMC)

The DMC will meet once prior to recruitment of the first participant and then convene prior to each TSC meeting to review the adverse event data and any other ethical aspects that arise and report to the TSC. The DMC will comprise an independent Chairperson plus at least two others.

12.4 Patient and Public Involvement (PPI)

A PPI group has provided input from inception and contributed to the design and practical implications. A PPI representative is a Co-Investigator for the trial and was involved in developing the grant application. PPI will continue throughout the trial via a public advisory group who will co-produce public-facing materials, support recruitment, retention and qualitative research, help to trouble shoot, and co-produce trial results.

12.5 Sponsorship, Host and Funding Organisation

The Sponsor is the University of Bristol and is responsible for overall oversight of the trial.

The Host is NHS Bristol, North Somerset and South Gloucestershire ICB who will ensure NHS engagement and their Research Team will support the project. The Host will be responsible for delivering the contract and will work with the Sponsor to monitor and manage supplier contracts.

This trial (project number NIHR131758) is funded by the Efficacy and Mechanism Evaluation (EME) programme, a Medical Research Council (MRC) and National Institute for Health and Care Research (NIHR) partnership. The views expressed in this protocol are those of the authors and not necessarily those of the MRC, NIHR or the Department of Health and Social Care.

This study is supported by the Bristol Trials Centre, a UKCRC registered clinical trials unit, which is in receipt of NIHR CTU support funding.

13. MONITORING, AUDIT & INSPECTION

13.1 Monitoring

The trial will be monitored and audited in accordance with the Sponsor’s policy, which is consistent with the UK Policy Framework for Health and Social Care Research. All trial related documents will be made available on request for monitoring and audit by the Sponsor, the relevant REC and for inspection by other regulatory bodies.

Ten percent of all studies sponsored by the University of Bristol are randomly selected each year for monitoring, conducted on behalf of the Sponsor by UHBW.

The Sponsor usually delegates some of the monitoring to the central research team. Checks of the following would be typical:

- Written informed consent has been properly documented
- Data collected are consistent with adherence to the trial protocol
- CRFs are only being completed by authorised persons
- SAE recording and reporting procedures are being followed correctly
- No key data are missing
- Data are valid
- Review of recruitment rates, withdrawals and losses to follow up

The percentage of patients that meet the eligibility criteria may be monitored and the percentage of participants who consent will be reported. To assess the generalisability of the participants, the

characteristics of consenting participants and non-consenting will be compared. If requested, preliminary data on adverse event and dropout rates observed in the trial population will be reported to the DMC.

13.2 Protocol Compliance

There will be no prospective, planned deviations or waivers to the protocol. Any protocol deviations will be documented and reported to the Trial Manager, CI and Sponsor immediately. Information about protocol breaches will also be included in routine reports to the TSC and DMC. In the event of systematic protocol deviations, investigation and remedial action will be taken in liaison with the CI, DMC and the TMG.

Any potentially serious protocol breach will be reported to the Sponsor as soon as possible. The Sponsor will determine whether it constitutes a serious breach, requiring onward reporting to the REC.

13.3 Notification of Serious Breaches to GCP or the protocol

A "serious breach" is a breach which is likely to effect to a significant degree:

- The safety or physical or mental integrity of the subjects of the trial; or
- The scientific value of the trial

The Sponsor will be notified immediately of any case where the above definition applies during the trial conduct phase. They will assess the seriousness of any breach.

14. ETHICAL AND REGULATORY CONSIDERATIONS

14.1 Governance and Legislation

This trial will be conducted in accordance with:

- Good Clinical Practice
- UK Policy Framework for Health and Social Care Research
- Data Protection Act 2018
- General Data Protection Regulation

Before any site can enrol participants into the trial, the CI or designee will obtain confirmation of capacity and capability (or equivalent organisation approval) for each site in-line with HRA processes along with other documentation required for the Sponsor to grant sites with a greenlight letter.

For all amendments, the CI or designee will confirm with the Sponsor, the HRA, REC and the site that permissions are ongoing.

This research trial will take into consideration ICH-GCP. ICH GCP is an international ethical and scientific quality standard for designing, conducting, recording, and reporting studies that involve the participation of human subjects. Compliance with GCP provides public assurance that the rights, safety, and well-being of trial participants are protected, consistent with the principles that originated in the Declaration of Helsinki and that the clinical trial data are credible.

14.2 Research Ethics Committee (REC) Review and Reports

Ethics review of the protocol for the trial and other trial related participant facing documents e.g., consent form, will be carried out by a UK Research Ethics Committee (REC). Any amendments to these documents will be approved by the Sponsor before being submitted to the REC/HRA for approval prior to implementation.

All correspondence with the REC will be retained in the Trial Master File (TMF). An annual progress report will be submitted to the REC within 30 days of the anniversary date on which the favourable opinion was given, and annually until the trial is declared ended. The CI will notify the REC of the end of the trial and if the trial is ended prematurely (including the reasons for the premature termination). Within one year after the end of the trial, the CI will submit a final report with the results, including any publications/abstracts, to the REC.

GCP training will be carried out by certain staff members depending on their delegated responsibilities within the trial, the level of training required will be determined according to the NIHR Delegation and Training Decision Aid. Informed consent to participate in the trial will be sought and obtained according to GCP guidelines.

14.3 Peer Review

The proposal for this trial has been peer-reviewed through the NIHR EME process, which includes independent expert and lay reviewers.

14.4 Regulatory Compliance

The trial will comply with the necessary regulations and will not commence until a favourable REC opinion and HRA approval have been provided, and sponsorship is issued.

14.5 Data Quality

The quality of the trial data will be monitored throughout the trial and data completeness will be reported to the TSC and DMC, and any cause for concern over data quality will be highlighted and an action plan put in place.

14.6 Financial and Other Competing Interests

The research team and all PIs must disclose any ownership interests that may be related to products, services, or interventions considered for use in the trial or that may be significantly affected by the trial. Competing interests will be reported in all publications and in the final report.

14.7 Indemnity

The necessary trial insurance is provided by the Sponsor.

15. DISSEMINATION POLICY

A plan for disseminating the trial results will be developed by the TMG.

The main results of the trial will be published in a high impact peer-reviewed journal. Initial findings will be submitted to relevant national and international meetings. Innovative methods of dissemination will be explored such as videos, YouTube clips and blogs to accompany scientific papers that are accessible to participants as well as providing a lay summary.

On completion of the trial a final report will be prepared for the Funder and once approved, made publicly available on their website.

16. SIGNATURE PAGE

The undersigned confirm that the following protocol has been agreed and accepted and that the Chief Investigator agrees to conduct the trial in compliance with the approved protocol and will adhere to the principles outlined in UK Policy Framework for Health and Social Care Research, the Sponsor's SOPs, and other regulatory requirements as amended.

I agree to ensure that the confidential information contained in this document will not be used for any other purpose other than the evaluation or conduct of the clinical investigation without the prior written consent of the Sponsor.

I also confirm that I will make the findings of the trial publicly available through publication or other dissemination tools without any unnecessary delay and that an honest accurate and transparent account of the trial will be given; and that any discrepancies from the trial as planned in this protocol will be explained.

Chief Investigator:

Signature:

.... Date: ....../......./.............

Name (please print):

....

Senior Statistician:

Signature:

.... Date: ....../......./.............

Name (please print):

....

17. AMENDMENT HISTORY

Record of protocol version numbers and amendments

Version		Notes
Number	Date	
1.0	10 AUG 2022	Pre-approved protocol submitted to REC
2.0	04 OCT 2022	Included changes following REC review. First approved protocol
3.0	06 MAR 2023	Clarification of roles and study procedures, update to qualitative interview process, clarification on blinding of TMG members

18. APPENDIX 1

This table shows the questions and responses that will be used to collect mechanistic data on participant and Study Clinician views at the points indicated.

**Mediating variable**	**Measures to be used (steps according to Figure 1)[A]**	**Response options**
Participant Views (or parent/carer if the participant is <16 years)		
Expectation that antibiotics are needed	1. I believe an antibiotic is needed to treat my/my child's illness [Steps 1, 3 and 7]<br>2. I believe my/my child's illness will get better faster if I/they take an antibiotic [Steps 1, 3 and 7]<br>3. I believe my/my child's illness will be less severe if I/they am/are given an antibiotic [Steps 1, 3 and 7]	Strongly disagree; Disagree; Neither agree nor disagree; Agree; Strongly agree
Confidence to manage illness without antibiotics (self-efficacy)	4. A point-of-care test would help in making the right decision about whether I/my child need/needs antibiotics [Step 3]<br>5. A point-of-care test would have helped[B]/has helped[C] in making the right decision about whether I/my child need/needs antibiotics [Step 7]<br>6. I am confident that I/my child will get/am getting/is getting the right treatment [Steps 3 and 7]	Strongly disagree; Disagree; Neither agree nor disagree; Agree; Strongly agree
Confidence to manage similar infection without antibiotics in the future	7. If I/my child have/has an infection in future that is like the one I/they had when I/they joined this trial then I/we will see my/our doctor to check if antibiotics are needed [2 Month Questionnaire]<br>8. If I/my child have/has an infection in future that is like the one I/they had when I/they joined this trial then I/we would like to have a point-of-care test to check if antibiotics are needed [2 Month Questionnaire]	Strongly disagree; Disagree; Neither agree nor disagree; Agree; Strongly agree
Study Clinician Views		
Expectation that antibiotics are needed	1. I believe an antibiotic is needed to treat the patient's illness [Steps 2 and 5]<br>2. I believe the patient's illness will improve faster if I prescribe an antibiotic [Steps 2 and 5]<br>3. I believe the patient's illness will be less severe if I prescribe an antibiotic [Steps 2 and 5]	Strongly disagree; Disagree; Neither agree nor disagree; Agree; Strongly agree
Confidence to manage patient without antibiotics (self-efficacy)	4. The point-of-care test would help in making the right decision about whether the patient needs antibiotics [Step 2]<br>5. The point-of-care test would have helped[B]/has helped[C] in making the right decision about whether the patient needs antibiotics [Step 5]<br>6. I am confident that the patient will believe they are getting the right treatment [Steps 2 and 5]	Strongly disagree; Disagree; Neither agree nor disagree; Agree; Strongly agree

Confidence to manage similar infection without antibiotics in the future	7. If a patient has a similar infection in future I am likely to prescribe them antibiotics [Step 5]<br>8. If a patient has a similar illness in future I would like to use the POCT^{RM} [Step 5]	Strongly disagree; Disagree; Neither agree nor disagree; Agree; Strongly agree

[A]Not presented in order collected
[B]Wording for control group
[C]Wording for intervention group

The items are based broadly on social cognitive theory,[62] although the constraints and demands of the trial mean that they also need to integrate other relevant theoretical constructs (as it is not possible to have multiple items to measure each relevant psychological construct). The first item in the Study Clinicians list is our primary mechanistic outcome and can be considered to evaluate perceived necessity of antibiotics (from the Necessity-Concerns framework[83]), which is likely to be influenced by the constructs from Social Cognitive Theory of 'outcome expectancies' for the consequences of prescribing/not prescribing antibiotics (items 2 and 3) and confidence ('self-efficacy/response efficacy') that the illness can be managed appropriately by using the POCT^{RM} (items 4 and 5). The final two items (6 and 7) assess key relevant future behavioural intentions.[84]

19. REFERENCES

1. Ferkol T, Schraufnagel D. The global burden of respiratory disease. Ann Am Thorac Soc. 2014;11(3):404-6.
2. Dolk FCK, Pouwels KB, Smith DRM, Robotham JV, Smieszek T. Antibiotics in primary care in England: which antibiotics are prescribed and for which conditions? J Antimicrob Chemother. 2018;73(suppl_2):ii2-ii10.
3. Stuart B, Hounkpatin H, Becque T, Yao G, Zhu S, Alonso-Coello P, et al. Delayed antibiotic prescribing for respiratory tract infections: individual patient data meta-analysis. BMJ. 2021;373:n808-n.
4. Fleming-Dutra KE, Hersh AL, Shapiro DJ, Bartoces M, Enns EA, File TM, Jr., et al. Prevalence of Inappropriate Antibiotic Prescriptions Among US Ambulatory Care Visits, 2010-2011. Jama. 2016;315(17):1864-73.
5. Pouwels KB, Dolk FCK, Smith DRM, Robotham JV, Smieszek T. Actual versus 'ideal' antibiotic prescribing for common conditions in English primary care. J Antimicrob Chemother. 2018;73(suppl_2):19-26.
6. Smith SM, Fahey T, Smucny J, Becker LA. Antibiotics for acute bronchitis. Cochrane Database Syst Rev. 2017;6(6):CD000245-CD.
7. Arroll B. Antibiotics for upper respiratory tract infections: an overview of Cochrane reviews. Respiratory Medicine. 2005;99(3):255-61.
8. Venekamp RP, Sanders SL, Glasziou PP, Del Mar CB, Rovers MM. Antibiotics for acute otitis media in children. Cochrane Database Syst Rev. 2015;2015(6):Cd000219.
9. Spinks A, Glasziou PP, Del Mar CB. Antibiotics for sore throat. Cochrane Database Syst Rev. 2013;2013(11):Cd000023.
10. Gillies M, Ranakusuma A, Hoffmann T, Thorning S, McGuire T, Glasziou P, et al. Common harms from amoxicillin: a systematic review and meta-analysis of randomized placebo-controlled trials for any indication. Cmaj. 2015;187(1):E21-e31.
11. Elvers KT, Wilson VJ, Hammond A, Duncan L, Huntley AL, Hay AD, et al. Antibiotic-induced changes in the human gut microbiota for the most commonly prescribed antibiotics in primary care in the UK: a systematic review. BMJ Open. 2020;10(9):e035677.
12. Little P, Gould C, Williamson I, Warner G, Gantley M, Kinmonth AL. Reattendance and complications in a randomised trial of prescribing strategies for sore throat: the medicalising effect of prescribing antibiotics. BMJ. 1997;315(7104):350-2.
13. Costelloe C, Metcalfe C, Lovering A, Mant D, Hay AD. Effect of antibiotic prescribing in primary care on antimicrobial resistance in individual patients: systematic review and meta-analysis. BMJ. 2010;340:c2096.
14. O'Neill J. Tackling Drug-Resistant Infections Globally: Final Report and Recommendations 2016 [Available from: https://amr-review.org/sites/default/files/160518_Final%20paper_with%20cover.pdf.
15. WHO. Global Action Plan on Antimicrobial Resistance 2015 [Available from: https://www.who.int/publications/i/item/9789241509763.
16. Horwood J, Cabral C, Hay AD, Ingram J. Primary care clinician antibiotic prescribing decisions in consultations for children with RTIs: a qualitative interview study. Br J Gen Pract. 2016;66(644):e207-13.
17. Cabral C, Lucas PJ, Ingram J, Hay AD, Horwood J. "It's safer to …" parent consulting and clinician antibiotic prescribing decisions for children with respiratory tract infections: An analysis across four qualitative studies. Soc Sci Med. 2015;136-137:156-64.
18. Department of Health and Social Care. Contained and controlled: The UK's 20-year vision for antimicrobial resistance 2019 [Available from: https://www.gov.uk/government/publications/uk-20-year-vision-for-antimicrobial-resistance.
19. Wellcome. The Global Response to AMR: Momentum, success, and critical gaps 2020 [Available from: https://wellcome.org/sites/default/files/wellcome-global-response-amr-report.pdf.
20. Quesada-González D, Merkoçi A. Nanomaterial-based devices for point-of-care diagnostic applications. Chem Soc Rev. 2018;47(13):4697-709.

21. Little P, Stuart B, Francis N, Douglas E, Tonkin-Crine S, Anthierens S, et al. Effects of internet-based training on antibiotic prescribing rates for acute respiratory-tract infections: a multinational, cluster, randomised, factorial, controlled trial. Lancet. 2013;382(9899):1175-82.
22. Cals JW, Butler CC, Hopstaken RM, Hood K, Dinant GJ. Effect of point of care testing for C reactive protein and training in communication skills on antibiotic use in lower respiratory tract infections: cluster randomised trial. BMJ. 2009;338:b1374.
23. National Clinical Guideline Centre (UK). National Institute for Health and Care Excellence: Clinical Guidelines. Pneumonia: Diagnosis and Management of Community- and Hospital-Acquired Pneumonia in Adults. London: National Institute for Health and Care Excellence (UK)Copyright © National Clinical Guideline Centre, 2014.; 2014.
24. Thornton HV, Khalid T, Hay AD. Point-of-care testing for respiratory infections during and after COVID-19. Br J Gen Pract. 2020;70(701):574-5.
25. Minnaard MC, van de Pol AC, de Groot JA, De Wit NJ, Hopstaken RM, van Delft S, et al. The added diagnostic value of five different C-reactive protein point-of-care test devices in detecting pneumonia in primary care: A nested case-control study. Scand J Clin Lab Invest. 2015;75(4):291-5.
26. bioMérieux. BIOFIRE® Respiratory 2.1 plus Panel 2021 [Available from: https://www.biomerieux.co.uk/biofirer-respiratory-panel-21-plus-sars-cov-2-ce-marked.
27. GenMarkDx. Respiratory Pathogen Panel 2021 [Available from: https://www.genmarkdx.com/panels/eplex-panels/respiratory-pathogen-panel/.
28. QIAstat-Dx. Respiratory SARS-CoV-2 Panel 2020 [Available from: https://qiastat-dx.com/row/wp-content/uploads/sites/3/2020/03/PROM-15948-001_1121481_FLY_QIAstat-Dx-SARS-CoV-2-CE-IVD_0320_ROW.pdf.
29. Brendish NJ, Malachira AK, Armstrong L, Houghton R, Aitken S, Nyimbili E, et al. Routine molecular point-of-care testing for respiratory viruses in adults presenting to hospital with acute respiratory illness (ResPOC): a pragmatic, open-label, randomised controlled trial. Lancet Respir Med. 2017;5(5):401-11.
30. Echavarría M, Marcone DN, Querci M, Seoane A, Ypas M, Videla C, et al. Clinical impact of rapid molecular detection of respiratory pathogens in patients with acute respiratory infection. Journal of Clinical Virology. 2018;108:90-5.
31. Rogers BB, Shankar P, Jerris RC, Kotzbauer D, Anderson EJ, Watson JR, et al. Impact of a rapid respiratory panel test on patient outcomes. Arch Pathol Lab Med. 2015;139(5):636-41.
32. Rappo U, Schuetz AN, Jenkins SG, Calfee DP, Walsh TJ, Wells MT, et al. Impact of Early Detection of Respiratory Viruses by Multiplex PCR Assay on Clinical Outcomes in Adult Patients. J Clin Microbiol. 2016;54(8):2096-103.
33. Lingervelder D, Koffijberg H, Kusters R, IJzerman MJ. Point-of-care testing in primary care: A systematic review on implementation aspects addressed in test evaluations. International Journal of Clinical Practice. 2019;73(10):e13392.
34. de Lusignan S, Hoang U, Liyanage H, Tripathy M, Yonova I, Byford R, et al. Integrating molecular point-of-care testing for influenza into primary care: a mixed-methods feasibility study. Br J Gen Pract. 2020;70(697):e555-e62.
35. Bruning AHL, de Kruijf WB, van Weert H, Willems WLM, de Jong MD, Pajkrt D, et al. Diagnostic performance and clinical feasibility of a point-of-care test for respiratory viral infections in primary health care. Fam Pract. 2017;34(5):558-63.
36. Khalid TY, Duncan LJ, Thornton HV, Lasseter G, Muir P, Toney ZA, et al. Novel multi-virus rapid respiratory microbiological point-of-care testing in primary care: a mixed-methods feasibility evaluation. Family Practice. 2021;38(5):598-605.
37. National Institute for Health and Care Effectiveness. Antimicrobial stewardship: systems and processes for effective antimicrobial medicine use 2015 [Available from: https://www.nice.org.uk/guidance/ng15.
38. Hay AD, Redmond NM, Turnbull S, Christensen H, Thornton H, Little P, et al. Development and internal validation of a clinical rule to improve antibiotic use in children presenting to primary care with acute respiratory tract infection and cough: a prognostic cohort study. Lancet Respir Med. 2016;4(11):902-10.

39. Little P, Moore M, Hobbs FD, Mant D, McNulty C, Williamson I, et al. PRImary care Streptococcal Management (PRISM) study: identifying clinical variables associated with Lancefield group A β-haemolytic streptococci and Lancefield non-Group A streptococcal throat infections from two cohorts of patients presenting with an acute sore throat. BMJ Open. 2013;3(10):e003943.
40. Ieven M, Coenen S, Loens K, Lammens C, Coenjaerts F, Vanderstraeten A, et al. Aetiology of lower respiratory tract infection in adults in primary care: a prospective study in 11 European countries. Clin Microbiol Infect. 2018;24(11):1158-63.
41. Thornton HV, Blair PS, Lovering AM, Muir P, Hay AD. Clinical presentation and microbiological diagnosis in paediatric respiratory tract infection: a systematic review. Br J Gen Pract. 2015;65(631):e69-81.
42. Thornton HV, Hay AD, Redmond NM, Turnbull SL, Christensen H, Peters TJ, et al. Throat swabs in children with respiratory tract infection: associations with clinical presentation and potential targets for point-of-care testing. Fam Pract. 2017;34(4):407-15.
43. Coughtrie AL, Whittaker RN, Begum N, Anderson R, Tuck A, Faust SN, et al. Evaluation of swabbing methods for estimating the prevalence of bacterial carriage in the upper respiratory tract: a cross sectional study. BMJ Open. 2014;4(10):e005341.
44. Tu Y-P, Jennings R, Hart B, Cangelosi GA, Wood RC, Wehber K, et al. Swabs Collected by Patients or Health Care Workers for SARS-CoV-2 Testing. New England Journal of Medicine. 2020;383(5):494-6.
45. Zlateva KT, de Vries JJC, Coenjaerts FEJ, van Loon AM, Verheij T, Little P, et al. Prolonged shedding of rhinovirus and re-infection in adults with respiratory tract illness. European Respiratory Journal. 2014;44(1):169-77.
46. Kaiser L, Lew D, Hirschel B, Auckenthaler R, Morabia A, Heald A, et al. Effects of antibiotic treatment in the subset of common-cold patients who have bacteria in nasopharyngeal secretions. Lancet. 1996;347(9014):1507-10.
47. Kaiser L, Morabia A, Stalder H, Ricchetti A, Auckenthaler R, Terrier F, et al. Role of Nasopharyngeal Culture in Antibiotic Prescription for Patients with Common Cold or Acute Sinusitis. European Journal of Clinical Microbiology and Infectious Diseases. 2001;20(7):445-51.
48. Thornton HV, Turner KME, Harrison S, Hammond A, Hawcroft C, Hay AD. Assessing the potential of upper respiratory tract point-of-care testing: a systematic review of the prognostic significance of upper respiratory tract microbes. Clin Microbiol Infect. 2019;25(11):1339-46.
49. Ordóñez-Mena JM, Fanshawe TR, Butler CC, Mant D, Longhurst D, Muir P, et al. Relationship between microbiology of throat swab and clinical course among primary care patients with acute cough: a prospective cohort study. Fam Pract. 2020;37(3):332-9.
50. McGeoch LJ BP, Christensen H, Muir P, Hay AD. . The relationship between respiratory tract viruses detected on pharyngeal swabs and day 2 to 4 symptom severity among children presenting to primary care with respiratory tract infection (RTI) symptoms. PLoS ONE (accepted). 2022.
51. Watson L, Little P, Moore M, Warner G, Williamson I. Validation study of a diary for use in acute lower respiratory tract infection. Fam Pract. 2001;18(5):553-4.
52. Butler CC, van der Velden AW, Bongard E, Saville BR, Holmes J, Coenen S, et al. Oseltamivir plus usual care versus usual care for influenza-like illness in primary care: an open-label, pragmatic, randomised controlled trial. Lancet. 2020;395(10217):42-52.
53. EuroQol. EQ-5D 2022 [Available from: https://euroqol.org/eq-5d-instruments/?msclkid=d9f35ab1c22611ecbea1ff027949a0fd.
54. HRA M. Joint statement on seeking consent by electronic methods 2018 [Available from: https://s3.eu-west-2.amazonaws.com/www.hra.nhs.uk/media/documents/hra-mhra-econsent-statement-sept-18.pdf.
55. Cole A. GPs feel pressurised to prescribe unnecessary antibiotics, survey finds. BMJ : British Medical Journal. 2014;349:g5238.
56. Butler CC, Simpson S, Wood F. General practitioners' perceptions of introducing near-patient testing for common infections into routine primary care: A qualitative study. Scandinavian Journal of Primary Health Care. 2008;26(1):17-21.
57. Jones CH, Howick J, Roberts NW, Price CP, Heneghan C, Plüddemann A, et al. Primary care clinicians' attitudes towards point-of-care blood testing: a systematic review of qualitative studies. BMC Fam Pract. 2013;14:117.

58. Wood F, Brookes-Howell L, Hood K, Cooper L, Verheij T, Goossens H, et al. A multi-country qualitative study of clinicians' and patients' views on point of care tests for lower respiratory tract infection. Fam Pract. 2011;28(6):661-9.
59. Tonkin-Crine S, Anthierens S, Francis NA, Brugman C, Fernandez-Vandellos P, Krawczyk J, et al. Exploring patients' views of primary care consultations with contrasting interventions for acute cough: a six-country European qualitative study. npj Primary Care Respiratory Medicine. 2014;24(1):14026.
60. Laurence CO, Gialamas A, Bubner T, Yelland L, Willson K, Ryan P, et al. Patient satisfaction with point-of-care testing in general practice. Br J Gen Pract. 2010;60(572):e98-104.
61. Bandura A. Health Promotion by Social Cognitive Means. Health Education & Behavior. 2004;31(2):143-64.
62. Bandura A. Social cognitive theory of self-regulation. Organizational Behavior and Human Decision Processes. 1991;50(2):248-87.
63. Yardley L, Douglas E, Anthierens S, Tonkin-Crine S, O'Reilly G, Stuart B, et al. Evaluation of a web-based intervention to reduce antibiotic prescribing for LRTI in six European countries: quantitative process analysis of the GRACE/INTRO randomised controlled trial. Implement Sci. 2013;8:134.
64. Gale NK, Heath G, Cameron E, Rashid S, Redwood S. Using the framework method for the analysis of qualitative data in multi-disciplinary health research. BMC Med Res Methodol. 2013;13:117.
65. Coast J JL. Understanding primary data analysis. In Qualitative methods for health economics. . J. C, editor2017.
66. Coast J, Al-Janabi H, Sutton EJ, Horrocks SA, Vosper AJ, Swancutt DR, et al. Using qualitative methods for attribute development for discrete choice experiments: issues and recommendations. Health Economics. 2012;21(6):730-41.
67. Ryan M. Discrete choice experiments in health care. BMJ. 2004;328(7436):360-1.
68. Ryan M, Gerad, K. Amaya-Amaya, M. Using Discrete Choice Experiments to Value Health and Health Care. 3300 AA Dordrecht, The Netherlands: Springer; 2008.
69. Soekhai V, de Bekker-Grob EW, Ellis AR, Vass CM. Discrete Choice Experiments in Health Economics: Past, Present and Future. PharmacoEconomics. 2019;37(2):201-26.
70. Lum EPM, Page K, Whitty JA, Doust J, Graves N. Antibiotic prescribing in primary healthcare: Dominant factors and trade-offs in decision-making. Infection, Disease & Health. 2018;23(2):74-86.
71. Morrell L, Buchanan J, Roope LSJ, Pouwels KB, Butler CC, Hayhoe B, et al. Delayed Antibiotic Prescription by General Practitioners in the UK: A Stated-Choice Study. Antibiotics. 2020;9(9):608.
72. Ancillotti M, Eriksson S, Andersson DI, Godskesen T, Nihlén Fahlquist J, Veldwijk J. Preferences regarding antibiotic treatment and the role of antibiotic resistance: A discrete choice experiment. International Journal of Antimicrobial Agents. 2020;56(6):106198.
73. Morrell L, Buchanan J, Roope LSJ, Pouwels KB, Butler CC, Hayhoe B, et al. Public preferences for delayed or immediate antibiotic prescriptions in UK primary care: A choice experiment. PLoS Med. 2021;18(8):e1003737.
74. Vosper AJ, Coast, J., Flynn, T. Qualitative Methods in Discrete Choice Experiments: Two Case Studies. Chapter 10 In Qualitative Methods for Health Economics. J. C, editor. London: Rowman & Littlefield International; 2017.
75. de Bekker-Grob EW, Donkers B, Jonker MF, Stolk EA. Sample Size Requirements for Discrete-Choice Experiments in Healthcare: a Practical Guide. Patient. 2015;8(5):373-84.
76. Reed Johnson F, Lancsar E, Marshall D, Kilambi V, Mühlbacher A, Regier DA, et al. Constructing Experimental Designs for Discrete-Choice Experiments: Report of the ISPOR Conjoint Analysis Experimental Design Good Research Practices Task Force. Value in Health. 2013;16(1):3-13.
77. Lancsar E, Fiebig DG, Hole AR. Discrete Choice Experiments: A Guide to Model Specification, Estimation and Software. PharmacoEconomics. 2017;35(7):697-716.
78. Hay AD, Little P, Harnden A, Thompson M, Wang K, Kendrick D, et al. Effect of Oral Prednisolone on Symptom Duration and Severity in Nonasthmatic Adults With Acute Lower Respiratory Tract Infection: A Randomized Clinical Trial. Jama. 2017;318(8):721-30.
79. NHS. GP Patient Survey 2022 [Available from: https://gp-patient.co.uk/.
80. Ridd MJ, Lewis G, Peters TJ, Salisbury C. Patient-Doctor Depth-of-Relationship Scale: Development and Validation. The Annals of Family Medicine. 2011;9(6):538-45.

81. Roope LSJ, Smith RD, Pouwels KB, Buchanan J, Abel L, Eibich P, et al. The challenge of antimicrobial resistance: What economics can contribute. Science. 2019;364(6435).
82. Moure-Fernandez A, Hollinghurst S, Carroll FE, Downing H, Young G, Brookes S, et al. Economic evaluation of the OSAC randomised controlled trial: oral corticosteroids for non-asthmatic adults with acute lower respiratory tract infection in primary care. BMJ Open. 2020;10(2):e033567.
83. Horne R, Chapman SC, Parham R, Freemantle N, Forbes A, Cooper V. Understanding patients' adherence-related beliefs about medicines prescribed for long-term conditions: a meta-analytic review of the Necessity-Concerns Framework. PLoS One. 2013;8(12):e80633.
84. Fishman J, Lushin V, Mandell DS. Predicting implementation: comparing validated measures of intention and assessing the role of motivation when designing behavioral interventions. Implementation Science Communications. 2020;1(1):81.
