# Supplementary material for: Rapid respiratory microbiological point-of-care-testing and antibiotic prescribing in primary care: Protocol for the RAPID-TEST randomised controlled trial
Source: PLoS One. 2024 May 20;19(5):e0302302. doi: 10.1371/journal.pone.0302302 (PMC11104596; doi:10.1371/journal.pone.0302302)
Supplement: S3 File — (PDF) [file pone.0302302.s005.pdf]

Rapid respiratory microbiological point-of-care-testing in primary care (RAPID-TEST trial)

Participant Information Sheet

Trial Summary

- We (the Sponsor at the University of Bristol) are inviting patients **aged 12 months or older with a respiratory tract infection** to take part in the RAPID-TEST trial.
- The trial plans to see if the use of a rapid microbiological Point-of-Care-Test (POCT^{RM}) for suspected respiratory tract infections in primary care changes how your infection is treated.
- The POCT^{RM} being used in this trial is a special machine located at your GP practice that helps us look for respiratory viruses and bacteria that may be causing your symptoms.
- If you take part, you will be asked to provide a nose and throat swab and will be allocated to one of the following two groups:
  (1) for the swab to be tested that day at your GP practice using the POCT^{RM} and for you and your GP to receive the results. Your swab will also be tested at a later date by a central laboratory.
  (2) for the swab to be tested at a later date by a central laboratory using the POCT^{RM}. Neither you nor your GP will receive the results.
- If you are allocated to have your swab tested that day at the GP, the results from the POCT^{RM} may be used to help decide your treatment.
- In both groups, you will be asked to complete a daily Trial Diary with details of your symptoms, medications and general health until your symptoms have resolved or for up to 28 days (whichever is sooner). You will also be asked to complete a short questionnaire at 2 months.
- If you consent to take part in the trial, you can leave at any time without giving a reason.

How to take part

Before you decide to take part, **please take time to read this information sheet**. It is important that you understand what the trial is about, why it is being done, and what it would involve for you.

Please ask if there are any parts of this information sheet that you do not understand or if you would like further information.

Contact details

If you have any questions about this trial at any time please contact your local research team at:

<Local GP practice contact details>

You may also wish to contact the central research team at Bristol Trials Centre: rapidtest-study@bristol.ac.uk

Thank you

Thank you for taking the time to read this information sheet and for considering your participation in the RAPID-TEST trial.

What is the purpose of the trial?

The main purpose of the RAPID-TEST trial is to investigate whether the use of a rapid microbiological Point-of-Care-Test (POCT^{RM}) can reduce same-day antibiotic prescribing for patients presenting to their GP with a respiratory tract infection. To take part, the GP or the patient should believe that antibiotic treatment is, or may be, necessary to treat the infection.

We are interested in whether the use of a rapid POCT^{RM} changes the following:

- Patient confidence in the management of their respiratory tract infection
- How long symptoms last and the severity of symptoms
- How often participants consult their GP for a respiratory tract infection, whether they receive

antibiotic or antiviral treatment or are admitted to hospital in the next 28 days
- Participant views on consulting their GP for similar future illnesses within 2 months
- The number of times participants contact their GP about a respiratory tract infection within 6 months

Finally, we are interested in participant views of the POCT^{RM} and their views about taking antibiotics now and in the future.

Why is the trial being done?

Every year, millions of people seek help for respiratory tract infections including coughs, colds, chest infections, sore throats and earache. GPs treat over half of these patients with antibiotics when this may not be appropriate. Antibiotics can have side effects and overuse means they will be less effective in the future.

Most respiratory infections are caused by viruses but antibiotics only work if the infection is caused by bacteria. If GPs knew whether a respiratory infection was caused by bacteria or a virus, they could prescribe antibiotics in a better way. The POCT^{RM} may help GPs to do this but at the moment this is unclear, which is why we are doing the RAPID-TEST trial.

This trial will also help us understand if using a POCT^{RM} can help patients feel better more quickly. The results from this study may then be used in future research to look into whether using a POCT^{RM} is cost-effective.

We need the help of 514 patients who present to their GP practice with a suspected respiratory tract infection. In addition, the GP or the patient should believe that antibiotic treatment is, or may be, necessary to treat the infection.

This trial has been designed by expert researchers and doctors, with the help of patients and members of the public.

Why have I been invited to take part?

You have been invited to take part in this trial because you have a suspected respiratory tract infection.

To be eligible to take part you need to:

- Be aged 12 months or older
- Your GP must suspect you have a respiratory tract infection
- Present to your GP practice for the first time in this illness and your illness must have started in the last 21 days. Symptoms may include one or more of: sore throat, runny nose, earache, cough, sputum, wheeze or shortness of breath.
- Have a diagnosis from a GP involved in the trial of an upper or lower respiratory tract infection such as: acute otitis media, acute sinusitis, acute pharyngitis or tonsillitis, sore throat, acute laryngitis, acute cough, acute bronchitis, chest infection, acute lower respiratory tract infection, infective exacerbation of chronic lung disease e.g. asthma, chronic obstructive pulmonary disease (COPD), emphysema or bronchiectasis
- Believe that antibiotic treatment is, or may be, necessary (or your GP needs to believe this is necessary).
- Be willing and able to give informed consent
- Be willing and able to have a nose and throat swab taken, or willing and able to collect, self-take and promptly return a swab to the GP practice. The swab must be at the GP practice that day in time for it to be transported to the hospital laboratory.
- Be willing to wait for the POCT^{RM} result (the GP must also be willing to wait) before an antibiotic prescribing decision is made
- Be willing to complete a Trial Diary and for data to be collected from your medical record

What is involved if I take part?

You will be asked to provide a nose and throat swab. You will then be allocated to either having the swab tested that day at your GP practice using the POCT^{RM} and receiving the results, or for the swab to be tested at a later date by a central laboratory using the POCT^{RM} and you will not receive any results.

In both groups, during the first day you present to your GP, you will be asked questions about your beliefs on use of antibiotics to treat your illness, your views on the POCT^{RM} and have consultations with your GP.

You will be asked to complete a Trial Diary about your symptoms, any antibiotic or antiviral medicines that you take and information about your health. The Trial Diary will need to be completed every day from when you present to your GP (Day 1) until your symptoms resolve or up to Day 28 (whichever is sooner). The trial research team will contact you regularly to support completion of the Trial Diary.

Two months after you present to your GP for this illness you will be asked to complete a short questionnaire about your views on use of antibiotics and the POCT^{RM} for any future similar illnesses. Around this point we will also collect information from your GP medical record on any GP appointments or hospital admissions for respiratory tract infections and whether antibiotic or antiviral treatment was prescribed up to Day 28. We will also look at your medical records again approximately six months after you started the trial to see if you have had any further GP appointments for respiratory tract infections.

You will be offered a £20 voucher on completion of the questionnaire at 2 months to thank you for your time.

Optional parts of the trial:

- If you agree, a member of the trial research team may contact you to discuss whether you would like to take part in an optional interview. This interview will be to explore your views and understanding of the trial and will take approximately 30 minutes. You will be offered a further £10 voucher on completion of the interview as a thank you for your time.

  Note: Once a sufficient number of participants have been interviewed, this optional part of the trial will close and further participants will not be contacted.

- If you agree, you will be sent an optional survey about your preferences and views related to the trial. This survey will take around 30 minutes to complete. You will be offered an additional £10 voucher on completion of the optional survey as a thank you for your time.

If you do not want to do either of these things you can still take part in the main trial.

Deciding whether to take part

Please remember:

- **Participation is voluntary**, it is your choice whether you take part in this trial, or not. Your usual care will not be affected either way.
- **If you do not understand anything** or have any questions, **please ask**. Contact details are at the top of page 1.
- If you do decide to take part, **you are free to leave the trial at any time,** without giving a reason.

What happens if I would like to go ahead?

A member of the trial research team/staff at the GP practice will contact you to answer any questions you may have, and ensure you understand what you are signing-up for. If you agree to take part:

- You will be assigned to an appointment with a GP involved in the trial to discuss whether you are eligible
- You will complete a consent form confirming that you understand the trial and are willing to take part
- Information on your illness will be collected so that we know how things are for you at the start of the trial
- You will provide a nose and throat swab sample
- You will be allocated by a computer to one of the following two groups:
  (1) for the swab to be tested that day at your GP practice using the POCT^{RM} and for you and your GP to receive the results. The swab will also be tested at a later date by a central laboratory.
  (2) for the swab to be tested at a later date by a central laboratory using the POCT^{RM}. Neither you nor your GP will receive the results.
  You will have a 50:50 chance of being in either group and neither you nor the GP can decide which group you will be in.

If you have **any concerns at any time about your symptoms,** please contact your GP for further advice. Taking part in the trial does not mean your health is monitored by the trial research team.

What will happen to the samples I provide?

You will be asked to provide a nose and throat swab as part of the trial. The sample will be stored securely and analysed at the GP practice and/or the central laboratory. A unique ID number and your full date of birth will be written on the sample to ensure it is identified correctly. Once the sample has been analysed, any remaining sample will be destroyed.

What are the possible benefits and downsides of taking part?

Possible benefits from taking part are:

- If you are in the group allocated to receiving the POCT^{RM} results, those results may help the GP decide whether or not an antibiotic is required to treat your respiratory tract infection
- Some people find taking part in research rewarding and may benefit from the extra contact from being part of the trial
- Even if you do not directly benefit from taking part in this trial, your involvement may help to inform future treatment recommendations for patients with respiratory tract infections

Possible downsides from taking part are:

- Participating in this trial will mean taking time out of your normal activities to allow the trial research team to collect information from you about your respiratory tract infection. The trial research team will also need to collect information about your views at three separate points during the day that you present to your GP (Day 1).
- You may need to wait longer than usual for the GP to make a decision about whether any antibiotic or antiviral treatment will be prescribed.
- You will also need to spend a couple of minutes every day completing the Trial Diary for up to 28 days and take time to complete the 2 Month Questionnaire.

If I take part, can I change my mind and leave the trial?

Yes. If you decide to take part, **you are free to leave the trial (withdraw) at any time**. You can do this by telling the trial research team (our contact details are on the first page).

It is very important that we try and get Trial Diary results from everyone who takes part in the trial, but if you decide to stop completing the Trial Diary, you can still remain in the trial by completing the 2 Month Questionnaire and allowing us to access your medical records for up to 6 months and you will still be helping.

If you want, you can withdraw from the trial completely. You would not have to give a reason for withdrawing, and your medical care and legal rights would not be affected. If this happened:

- We would use any information we have already collected about you in our analysis of the trial results.
- We may ask if you would be willing to discuss your reasons for withdrawing from the trial with a member of the trial research team.

What if new information becomes available during the trial?

Sometimes we get new information about the condition being studied. If this happens, the trial research team will tell you and discuss whether you should continue in the trial. If you decide not to continue, you would be withdrawn from the trial. If you did continue in the trial, you could be asked to sign an updated consent form.

How long does the trial last and what will happen to the results?

The trial is expected to run from Winter 2022 through to 2024.

Once the trial is completed, the overall results will be published in medical journals and presented at conferences attended by healthcare professionals and researchers.

The results will also be shared with the wider public using accessible summaries through our website and social media.

If you wish, we will also send you a newsletter with the results of the trial when it has finished.

No one will be able to identify you from any of the trial reports or publications.

Who funded this trial, who is the Sponsor, and who is managing this trial?

The trial is being funded by the research arm of the National Health Service (NHS), the National Institute for Health Research Efficacy and Mechanism Evaluation programme (NIHR EME reference 131758).

The research is led by a team of experienced doctors and researchers and is overseen (sponsored) by the University of Bristol.

The Bristol Trials Centre is responsible for running the trial.

Will my data be used in future research?

Other researchers may request to access **anonymised data** (i.e. data that you cannot be identified from) collected in this trial in the future, for example to combine the results of our trial with others similar studies. These researchers may be outside of the UK, EU and EEA.

If you take part in this trial, **anonymised data** collected in this trial may be used in future ethically approved studies; this will never include names or contact details, and it will not be possible to identify individual participants.

For more detailed information about how we will process, store and share any information that you give us, please see the blue pages at the end of this sheet.

Who has reviewed the trial?

All research in the NHS is looked at by an independent group of people, called a Research Ethics Committee (REC) to protect your safety, rights, wellbeing and dignity.

This trial has been reviewed and approved by North West - Preston Research Ethics Committee and the Health Research Authority.

Additionally, independent Data Monitoring and Trial Steering Committees will monitor the trial throughout its duration to ensure it is conducted according to good research practice.

What if there is a problem?

If you have a concern regarding your care as a patient, please discuss this with your GP or specialist.

We do not expect taking part to affect any private medical insurance, but if relevant, please check with your insurers before agreeing to take part in the trial.

If something goes wrong and you are harmed during the trial and this is due to someone's negligence then you may have grounds for legal action, but you may have to pay your legal costs. The normal NHS complaints mechanisms will still be available to you. The University of Bristol holds insurance to cover harm to participants as a result of this research. Please see details on how to file a complaint below.

What if I have a concern or complaint?

If you have any questions or concerns about any aspect of this trial, or your treatment or health whilst on the trial, please speak to a member of the trial research team using the details on page 1.

If you remain unhappy with any aspect of the trial, please email the sponsor (research-governance@bristol.ac.uk).

If you are still concerned and wish to complain formally about your healthcare or any aspect of this trial, you can do this through the NHS Complaints Procedure, either by post, telephone, or email.

Post: NHS England, PO Box 16738, Redditch, B97 9PT. **Telephone:** 0300 311 22 33. **Email:** england.contactus@nhs.net *(Please state: 'For the attention of the complaints team' in the subject line).*

You can visit their website for further information: https://www.england.nhs.uk/contact-us/complaint%20/complaining-to-nhse/

ABOUT MY DATA

If you are interested, these pages tell you more about how we process, store and share any information that you give us.

How will the information I provide be kept confidential?

We are committed to handling the information (data) used in the RAPID-TEST trial securely and confidentially. Your data will be stored and used in compliance with the relevant, current data protection laws; Data Protection Act 2018 and General Data Protection Regulation 2018 (GDPR).

We will use information from you and/or your medical records to undertake this trial and the University of Bristol will act as the data controller.

- This means that we are responsible for looking after your information and using it properly.
- Personal information such as your name, date of birth, email address, and phone number will be stored on a secure database managed by the central research team (at the University of Bristol).

The University of Bristol will securely keep identifiable information about you for at least five years after the trial has finished; this is considered good practice for clinical trials.

It is a requirement that your records in this research, together with any relevant medical records, can be looked at by authorised staff working for the Sponsor or the Regulatory Authorities. Their job is to check that research is properly conducted and the interests of those taking part are adequately protected.

With your permission, we may tell your doctor/GP if we have concerns about your health or well-being. However, if there is a risk of harm to you or others, we may share such information with your doctor without your consent.

How will we use information about you?

We will need to use information from you and your medical records for this research project.

This information will include your initials, NHS number, name and contact details. People will use this information to do the research or to check your records to make sure that the research is being done properly.

People who do not need to know who you are will not be able to see your name or contact details. Your data will have a code number instead.

We will keep all information about you safe and secure.

Once we have finished the trial, we will keep some of the data so we can check the results. We will write our reports in a way that no-one can work out that you took part in the trial.

You will have the option on the consent form to be contacted and informed about taking part in other future research. There is no obligation to take part and you would just be informed of what the future research will involve.

If you consent to recording of interviews, then the recordings will be securely transferred and transcribed in part or full by University of Bristol employees or their authorised representatives.

Transcripts will be anonymised so that you cannot be identified from them.

Audio recordings will be transcribed by an employee of the University of Bristol or an approved transcription company. Data will be transferred by means of secure filestores and transcribers will be bound by a confidentiality agreement.

The University of Bristol will securely retain audio-recorded data and may use anonymised quotations and parts of voice modified audio-recordings for training, teaching, research, and publication purposes

for this and future studies, but we will ensure that you cannot be identified.

With your permission, anonymised transcripts of audio-recordings may be made available by controlled access to other researchers outside of the RAPID-TEST trial who secure the necessary approvals. Again, we will ensure you cannot be identified.

Data from the anonymised transcripts may be used for purposes not related to this trial, but it will not be possible to identify you from them.

If you agree to take part in the RAPID-TEST trial, around 6 months after you joined the trial we will **collect information from your GP** about your health for the time you were taking part in the trial. This would not take up any of your time and would be done directly and securely with the GP practice.

Who will you share information about me with?

Sealed Envelope™ are the company who provide the randomisation software which helps to enable the process of allocation. We will provide Sealed Envelope with relevant information about you (age, presence or absence of chronic lung disease) to enable their system to allocate you to a group i.e. for the swab to be tested that day at your GP practice using the POCT^{RM} and to receive the results, or for the swab to be tested at a later date by a central laboratory and you will not receive the results. The information provided to them will be kept securely and they will not be given patient contact details.

University Hospitals Bristol and Weston NHS Foundation Trust (UHBW) carry out monitoring and safety reporting for the University of Bristol. To carry out these activities, UHBW may need to have access to your medical records where it is relevant to you taking part in the research.

If you sign up to receive the trial newsletter, which will give regular updates on the progress of the trial and news relating to the research, your email address will be stored by an **online newsletter provider** (such as MailChimp® or other similar provider), but cannot be used by them for any other purpose. You will be able to unsubscribe from the mailing list at any point, if you wish.

What are your choices about how your information is used?

- You can stop being part of the trial at any time, without giving a reason, but we will keep information about you that we already have.
- If you choose to stop taking part in the trial, we would like to continue collecting information about your health from your GP. If you do not want this to happen, tell us and we will stop.
- We need to manage your records in specific ways for the research to be reliable. This means that we won't be able to let you see or change the data we hold about you.

Where can you find out more about how your information is used?

You can find out more about how we use your information:

- At www.hra.nhs.uk/information-about-patients Our leaflet available from www.hra.nhs.uk/patientdataandresearch or www.bristol.ac.uk/secretary/data-protection/policy/research-participant-fair-processing-notice
- By asking one of the research team
- By sending an email to rapidtest-study@bristol.ac.uk

Acknowledgement and Disclaimer: This project (NIHR131758) is funded by the Efficacy and Mechanism Evaluation (EME) Programme, an MRC and NIHR partnership. The views expressed in this publication are those of the author(s) and not necessarily those of the MRC, NIHR or the Department of Health and Social Care.

JOINTLY FUNDED BY UKRI Medical Research Council NIHR National Institute for Health and Care Research
