# Supplementary material for: Rapid respiratory microbiological point-of-care-testing and antibiotic prescribing in primary care: Protocol for the RAPID-TEST randomised controlled trial
Source: PLoS One. 2024 May 20;19(5):e0302302. doi: 10.1371/journal.pone.0302302 (PMC11104596; doi:10.1371/journal.pone.0302302)
Supplement: S4 File — (PDF) [file pone.0302302.s006.pdf]

Rapid respiratory microbiological point-of-care-testing in primary care (RAPID-TEST trial)

Informed Consent Form

Name of Principal Investigator: <insert name>

Participant ID: | | | | | |

**IF THE PARTICIPANT PROVIDED INFORMED CONSENT VERBALLY:** please tick this box. In this case, the researcher should provide the participant's responses in each of the statement boxes in the presence of a witness (any member of staff on duty who is not connected with the trial) who should also countersign this form (see final page). ☐

**Mandatory statements**<br>*These are mandatory and you can only take part in the trial if you answer "Yes" to all statements*		**Please tick**	
		**Yes**	**No**
**1.**	I confirm that I have read and understand the Participant Information Sheet, Version <insert current PIS version number and date> for the above trial. I have had the opportunity to consider the information, ask questions and have had these answered satisfactorily.		
**2.**	I understand that my participation in this trial is voluntary and that I am free to withdraw at any time, without giving any reason, and without my medical care or legal rights being affected. I understand that should I withdraw, then the information collected so far cannot be deleted and that this information may still be used in the trial analysis.		
**3.**	I understand that relevant sections of my medical notes and data collected during the trial may be looked at by individuals from the Bristol Trials Centre, the Sponsor (University of Bristol), NHS bodies and regulatory authorities where it is relevant to my taking part in this trial. I give permission for these individuals to have access to my medical records.		
**4.**	I understand that information about me will be shared with Sealed Envelope™ the company who provide the software that helps to enable the process of randomisation.		
**5.**	I understand I need to provide a nose and throat swab for the purpose of this trial and research associated with this trial. This swab sample will be transferred to a central research laboratory for testing. I agree to the collection, storage and analysis of this swab sample. All samples will be anonymised and I will not be identified in any way.		
**6.**	I understand that the Bristol Trials Centre and the trial research team will be provided with my personal details to contact me for follow-up. I give my permission for this information to be kept and for these individuals to contact me.		
**7.**	I give permission for the Bristol Trials Centre, the Sponsor and the trial research team to collect, store, analyse and publish information obtained from my participation in this trial. I understand that my personal details will be kept confidential.		

Participant ID: | | | | | |

**Mandatory statements**<br>*These are mandatory and you can only take part in the trial if you answer "Yes" to all statements*		**Please tick**<br>**Yes**	 **No**
**8.**	I understand that my personal data will be kept for at least 5 years after the end of the trial and held confidentially and securely by the research team.		
**9.**	I understand that the research data collected about me may be used anonymously to support other research in the future and may be shared anonymously with other researchers. These researchers may be outside of the UK, EU and EEA.		
**10.**	I agree to take part in the above trial.		

**Optional statements**<br>*These are optional and you can still take part in the trial if you answer "No"*		**Please tick**<br>**Yes**	 **No**
**11.**	I agree to be contacted by a member of the trial research team to discuss whether I would like to take part in the optional interview. The interview will be to explore my views and understanding of the trial.		
**12.**	I agree to be sent an optional survey about my preferences and views related to the trial.		
**13.**	I am happy to be added to the RAPID-TEST trial mailing list to receive trial newsletters and updates detailing the progress of the trial. These will be sent using Mailchimp or similar newsletter software who will store my email address. I will be able to opt out at any time.		
**14.**	I am willing to be contacted and informed about taking part in other future research. I understand that there is no obligation and I will just be informed of what the future research will involve.		

Your name in block capitals *(Participant)*

Your signature (where feasible)

__ __ / __ __ / 2 0 __ __

Today's Date (dd/mm/yyyy)

Researcher name in block capitals *(Person taking consent)*

Researcher signature

__ __ / __ __ / 2 0 __ __

Today's Date (dd/mm/yyyy)

Participant ID: | | | | | |

ONLY IF PARTICIPANT GAVE VERBAL INFORMED CONSENT (I.E. UNABLE TO USE E-CONSENT):
The participant is unable to sign. As a witness, I confirm that all the information about the trial was given and suitably discussed and the participant gave their informed consent to take part.

Witness name in block capitals

Witness signature

__ __ / __ __ / 2 0 __ __

Today's Date (dd/mm/yyyy)

INFORMATION FOR RESEARCHER:

When completed: 1 original for Site File, 1 original for participant. Electronic copies also to be provided to GP and Bristol Trials Centre.

Acknowledgement and Disclaimer: This project (NIHR131758) is funded by the Efficacy and Mechanism Evaluation (EME) Programme, an MRC and NIHR partnership. The views expressed in this publication are those of the author(s) and not necessarily those of the MRC, NIHR or the Department of Health and Social Care.

JOINTLY FUNDED BY Medical Research Council NIHR National Institute for Health and Care Research
